# Epigenetic Regulation of *F2RL3* Associates With Myocardial Infarction and Platelet Function

**DOI:** 10.1161/CIRCRESAHA.121.318836

**Published:** 2022-01-06

**Authors:** Laura J. Corbin, Stephen J. White, Amy E. Taylor, Christopher M. Williams, Kurt Taylor, Marion T. van den Bosch, Jack E. Teasdale, Matthew Jones, Mark Bond, Matthew T. Harper, Louise Falk, Alix Groom, Georgina G.J. Hazell, Lavinia Paternoster, Marcus R. Munafò, Børge G. Nordestgaard, Anne Tybjærg-Hansen, Stig E. Bojesen, Caroline Relton, Josine L. Min, George Davey Smith, Andrew D. Mumford, Alastair W. Poole, Nicholas J. Timpson

**Affiliations:** MRC Integrative Epidemiology Unit at University of Bristol, United Kingdom (L.J.C., L.F., A.G., L.P., M.R.M., C.R., J.L.M., G.D.S., N.J.T.).; Population Health Sciences, Bristol Medical School (L.J.C., A.E.T., K.T., L.F., A.G., L.P., C.R., J.L.M., G.D.S., N.J.T.), University of Bristol, United Kingdom.; School of Physiology, Pharmacology and Neuroscience (C.M.W., M.T.v.d.B., A.W.P.), University of Bristol, United Kingdom.; Translational Health Sciences, Bristol Medical School (J.E.T., M.J., M.B.), University of Bristol, United Kingdom.; UK Centre for Tobacco and Alcohol Studies and School of Experimental Psychology (M.R.M.), University of Bristol, United Kingdom.; School of Cellular and Molecular Medicine (A.D.M.), University of Bristol, United Kingdom.; Department of Life Sciences, Manchester Metropolitan University, United Kingdom (S.J.W.).; NIHR Biomedical Research Centre at the University Hospitals Bristol NHS Foundation Trust and the University of Bristol, United Kingdom (A.E.T.).; Department of Pharmacology, University of Cambridge, Tennis Court Road (M.T.H., G.G.J.H.).; Department of Clinical Biochemistry, Herlev and Gentofte Hospital (B.G.N., S.E.B.), Copenhagen University Hospital, Denmark.; The Copenhagen City Heart Study, Frederiksberg Hospital (B.G.N., A.T.-H., S.E.B.), Copenhagen University Hospital, Denmark.; Department of Clinical Biochemistry, Rigshospitalet (A.T.-H.), Copenhagen University Hospital, Denmark.; Faculty of Health and Medical Sciences, University of Copenhagen, Denmark (B.G.N., A.T.-H., S.E.B.).

**Keywords:** blood platelets, DNA methylation, epigenomics, myocardial infarction, smoking, thrombin, tobacco

## Abstract

DNA hypomethylation at the *F2RL3* (F2R like thrombin or trypsin receptor 3) locus has been associated with both smoking and atherosclerotic cardiovascular disease; whether these smoking-related associations form a pathway to disease is unknown. *F2RL3* encodes protease-activated receptor 4, a potent thrombin receptor expressed on platelets. Given the role of thrombin in platelet activation and the role of thrombus formation in myocardial infarction, alterations to this biological pathway could be important for ischemic cardiovascular disease.

**Methods:**

We conducted multiple independent experiments to assess whether DNA hypomethylation at *F2RL3* in response to smoking is associated with risk of myocardial infarction via changes to platelet reactivity. Using cohort data (N=3205), we explored the relationship between smoking, DNA hypomethylation at *F2RL3*, and myocardial infarction. We compared platelet reactivity in individuals with low versus high DNA methylation at *F2RL3* (N=41). We used an in vitro model to explore the biological response of *F2RL3* to cigarette smoke extract. Finally, a series of reporter constructs were used to investigate how differential methylation could impact *F2RL3* gene expression.

**Results:**

Observationally, DNA methylation at *F2RL3* mediated an estimated 34% of the smoking effect on increased risk of myocardial infarction. An association between methylation group (low/high) and platelet reactivity was observed in response to PAR4 (protease-activated receptor 4) stimulation. In cells, cigarette smoke extract exposure was associated with a 4.9% to 9.3% reduction in DNA methylation at *F2RL3* and a corresponding 1.7-(95% CI, 1.2–2.4, *P*=0.04) fold increase in *F2RL3* mRNA. Results from reporter assays suggest the exon 2 region of *F2RL3* may help control gene expression.

**Conclusions:**

Smoking-induced epigenetic DNA hypomethylation at *F2RL3* appears to increase PAR4 expression with potential downstream consequences for platelet reactivity. Combined evidence here not only identifies *F2RL3* DNA methylation as a possible contributory pathway from smoking to cardiovascular disease risk but from any feature potentially influencing *F2RL3* regulation in a similar manner.

## Abstract 2

Supplemental Digital Content is available in the text.

Epigenetic regulation through DNA methylation is a process that changes the properties of DNA molecules, enabling the regulation of gene expression without changing the DNA sequence itself. The use of array-based DNA methylation measurement technologies has revealed smoking-related differential DNA methylation patterns in DNA extracted from peripheral blood.^1–5^ Cigarette smoking is associated with a broad pattern of differential methylation, with the largest studies flagging several thousand cytosine–phosphate–guanine (CpG) sites.^4^ These relationships have been used to suggest methylation as a potential mechanism by which tobacco exposure predisposes to adverse health outcomes. Indeed, robust associations between smoking-methylation signatures and smoking-related pathology such as lung cancer^6^ have been demonstrated. Despite increasing evidence of the impact of environmental and genetic exposures on health via DNA methylation, there is a notable lack of studies in the literature that track pathways from environmental exposure (eg, smoking) to change in methylation and on to disease end points. To do this requires studies of a composite nature that can survey observational relationships at a population scale, verify functional change using careful study design and test appropriate mechanisms within a basic science setting.

The existing literature provides strong justification for a focused investigation of the role of epigenetic modification of *F2RL3* (F2R like thrombin or trypsin receptor 3) in mediating the increased risk of thrombotic disease in smokers. cg03636183 in *F2RL3* was one of the first CpG sites to be robustly associated with smoking and appears to show a dose-response relationship.^7^ *F2RL3* codes for PAR4 (protease-activated receptor 4), a G-protein coupled receptor expressed on the surface of a number of cell types including platelets.^8^ Together with PAR1 (protease-activated receptor 1), PAR4 activates platelets in response to thrombin generated at the site of tissue injury. An absence of PAR4 in mouse models results in impaired hemostasis and a protection against pulmonary embolism,^9^ and a small number of missense coding variants in *F2RL3* that alter platelet aggregation and function have been described.^10–12^ In particular, the Thr120 variant of the common single nucleotide polymorphism (rs773902) has been highlighted as functionally relevant^13–15^ providing a link between PAR4 and the heritable interindividual variation in platelet reactivity. However, little is known about the functional consequences of epigenetic modifications (delivered either by environmental exposure or genetic variation) at the *F2RL3* locus and how regulatory shifts in the complex events controlling platelet function may manifest in health outcomes. Preliminary evidence exists suggesting that changes in DNA methylation can alter platelet activity with pathological consequences^16,17^ and that DNA hypomethylation at *F2RL3* is associated with mortality from cardiovascular disease.^18^ The primary objective of this work was to assess whether the smoking-related associations seen at this locus form a pathway to disease and if so, through what biological mechanisms.

Specifically, we hypothesized that smoking-induced epigenetic DNA hypomethylation at *F2RL3* could increase the risk of myocardial infarction through increased platelet reactivity. To test this, we aimed to triangulate^19^ evidence from a series of experiments, each designed to explore one or more steps in the proposed biological pathway. We extended previous epidemiological work^18^ by focusing specifically on thrombosis-driven myocardial infarction where platelet aggregation plays a key role. We explored DNA methylation-dependent regulation of platelet function in healthy individuals (in the absence of smoking). We used cell lines to understand the potential functional consequences of smoking-related changes in DNA methylation in vitro and followed this up with a gene reporter-based investigation of how changes in methylation could impact on gene function.

## Methods

### Data Availability

**Figure 1.**
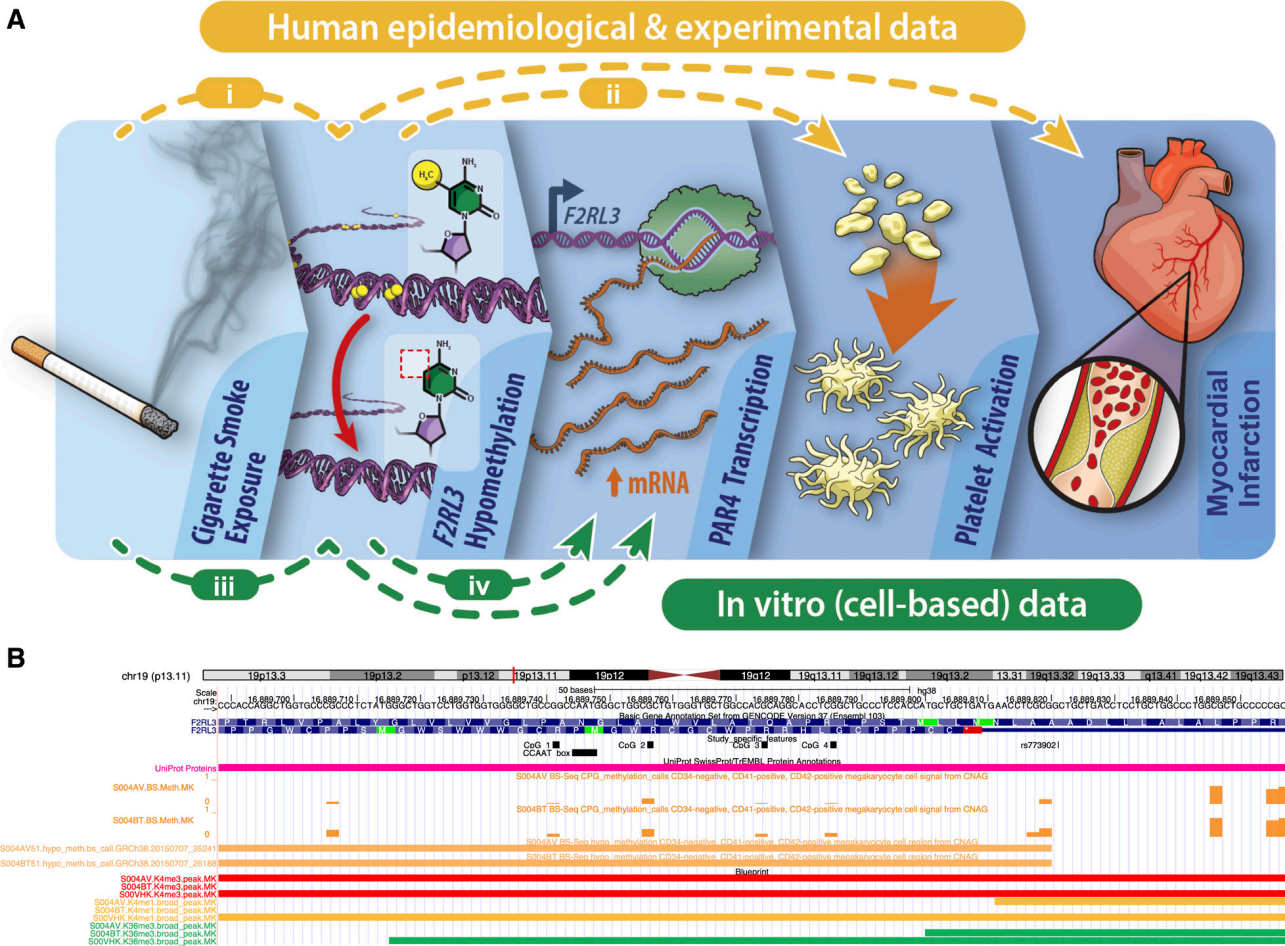
**Study overview.** **A**, Infographic to describe the 4 components of the study (i) in a population-based analysis (n=3205), the observational association between smoking, DNA hypomethylation of *F2RL3* and increased risk of myocardial infarction was quantified, focusing specifically on myocardial infarction. (ii) The impact of differential DNA methylation at *F2RL3* on platelet function and reactivity in humans was tested in a recall study. (iii) The effect of exposure to cigarette smoke extract on *F2RL3* DNA methylation, mRNA expression and protein abundance in acute megakaryocytic leukemia (CMK) cells was tested experimentally. (iv) An exploration of the functional regulation of *F2RL3* was conducted using a series of reporter constructs. **B**, UCSC browser view of the *F2RL3* exon 2 region containing the 4 cytosine–phosphate–guanine (CpG) sites assayed (chr19: 16 889 744-16 889 857 bp). Custom track study-specific features (black) shows the 4 DNA methylation sites CpG_1 to CpG_4 that were assessed by pyrosequencing, rs773902 and a CCAAT binding factor recognition sequence. CpG_3 corresponds to the CpG labeled cg03636183 on the Illumina Infinium Human Methylation450 BeadChip (450 K) array. Selected epigenetic annotations integrated from BLUEPRINT based on data from a megakaryocyte cell lineage. Figure produced using UCSC Genome Browser^58^ based on Genome Reference Consortium Human Build 38 (GRCh38/hg38). For a full gene view and additional epigenetic annotations see Figure S10A.

Because of the sensitive nature of the data used in this study, requests to access the data presented in sections Smoking, DNA hypomethylation and myocardial infarction and Association between DNA hypomethylation and platelet function must be made directly to the relevant cohorts. Data from ALSPAC (Avon Longitudinal Study of Parents and Children) will be made available on request to the ALSPAC executive committee (alspac-exec@bristol.ac.uk); the ALSPAC data management plan (available here: http://www.bristol.ac.uk/alspac/researchers/data-access/) describes in detail the policy regarding data sharing, which is through a system of managed open access. Data from Copenhagen City Heart Study is made available to researchers upon application to the Steering Committee. Source Data and corresponding statistical analysis results described in sections In vitro association between smoking and F2RL3 DNA hypomethylation and expression and Functional regulation of F2RL3 have been made publicly available at the University of Bristol data repository, data.bris, and can be accessed at: https://doi.org/10.5523/bris.35olm77woaku72652wg0g0j6nf. The R and Stata code that support the findings of this study are available from the corresponding author upon reasonable request. An overview of the study and how each of 4 experiments conducted relate to the hypothesized pathway is presented in Figure 1A. In experiments (1) to (3), DNA methylation was quantified by pyrosequencing of 4 CpG sites located in exon 2 of *F2RL3* at 16 889 741-2bp (CpG_1), 16 889 756-7bp (CpG_2), 16 889 774-5bp (CpG_3), and 16 889 785-6bp (CpG_4; GRCh38/hg38; Figure 1B). CpG_3 is also present on the Illumina Infinium Human Methylation 450K BeadChip array (designated cg03636183).

Further methodological details can be found under headings corresponding to those below in Methods in the Supplemental Material and in the corresponding Table. No correction was made for multiple testing, and all *P* values presented in the results are unadjusted.

#### Smoking, DNA Hypomethylation, and Myocardial Infarction

Using individual participant data from the Copenhagen City Heart Study, we explored the relationship between smoking, DNA hypomethylation at *F2RL3* and myocardial infarction morbidity and mortality in an observational framework. The Copenhagen City Heart Study is a prospective longitudinal cohort study of the Danish general population with the first examination having taken place from 1976 through to 1978. DNA methylation extent was measured in samples collected at the third examination which took place from 1991 through 1994^20^ and is referred to herein as baseline. Of the 9234 participants who attended this clinic, 4292 participants were selected for inclusion in our study, including all those who had experienced a myocardial infarction based on records up to April 2013 (N=1125). Up to 4 control samples were selected per case based on a matching scheme involving age, sex, and smoking status (defined as never, former, or current). The objective of this matching scheme was to ensure the distribution of these variables in the control arm of the study would approximately match that in the case group. Pyrosequencing was carried out at the 4 CpG sites (CpG_1-4) in exon 2 of *F2RL3* (Figure 1B). Before statistical analyses, participant case status for the primary outcome of (incident) myocardial infarction status was updated based on patient records up to 10 November, 2014. For the secondary outcome of mortality following (incident) myocardial infarction, individuals were censored upon death or 14 November, 2014 if they were alive on this date.

##### Statistics

To characterize the previously published association between *F2RL3* methylation and smoking within our sample, associations between both self-reported smoking behaviour (expressed as smoking status—never, former, or current, doubling of pack years smoked or number of cigarettes smoked per day) and DNA methylation at the aryl hydrocarbon receptor repressor (*AHRR*) as exposures, and percentage DNA methylation at *F2RL3* at the 4 CpG sites as outcomes were assessed using linear regression. *AHRR* methylation extent was included in analyses as an independent (from *F2RL3*) locus at which methylation is reliably associated with smoking status and which can act as a more objective measure (or biomarker) of smoking exposure than self-report.^21^ All models included sex and age at baseline as covariates. Data on time since quitting in former smokers was used to evaluate the time taken for smoking-related changes in methylation extent to be recovered using linear regression. In a model fitted using data from former smokers with information on time since cessation only, methylation (at each of the CpG sites in turn) was the outcome and time since cessation (years) the exposure variable. The model was run with and without sex and age at baseline fitted as covariates and an estimate of the variation in methylation explained by time since cessation extracted from the unadjusted model.

To evaluate the potential role of *F2RL3* methylation in myocardial infarction, associations between *F2RL3* DNA methylation (per SD decrease at each of the 4 CpG sites) as an exposure and incident myocardial infarction as the primary outcome were assessed within smoking status groups. Case individuals who were diagnosed with myocardial infarction before baseline were excluded from all analyses. A series of Cox regression models were implemented, each with an increasing number of covariates, as detailed in Methods (i) in the Supplemental Material. Individuals entered the analysis at baseline with age accounted for by setting date of birth as the origin. Covariates fitted in the full model were sex, percentage DNA methylation at *AHRR* (to adjust for exposure to cigarette smoke), diabetes status (case/control), systolic blood pressure (continuously per 10 mm HG above 120 mm HG), total cholesterol (continuously per mmol/L above 5 mmol/L) and exposure to passive smoking (yes/no). Covariates were selected based on their role as risk factors for myocardial infarction according to the European Society of Cardiology.^22^ The association between *F2RL3* DNA methylation and the secondary outcome of mortality following incident myocardial infarction was assessed using a similar set of Cox regressions. Here, the time variable was the time between the myocardial event and death (or censoring) with age at myocardial infarction event fitted as a covariate in the model. We conducted sensitivity analyses to assess the potential for collider bias (induced by conditioning on myocardial infarction case status) to affect our results (Methods (i) and Results (i) in the Supplemental Material).

The extent to which DNA methylation at *F2RL3* mediates the association between smoking exposure (expressed as a doubling of pack years) and risk of myocardial infarction or death after myocardial infarction was estimated in ever smokers (both current and former smokers) by the product of coefficients method.^23^ Cox regression models were fitted to reflect the full model described above with the addition of smoking status (former/current) as a covariate and without adjustment for percentage DNA methylation at *AHRR*, since smoking is the exposure we aimed to capture (see Methods (i) in the Supplemental Material for full details). We also performed the mediation analysis with *AHRR* as the mediator to evaluate the extent to which mediation is specific to the *F2RL3* locus.

In all models, individuals with missing exposure, outcome or covariate data were excluded. All analyses were conducted in Stata (version 16.1).^24^

#### Association Between DNA Hypomethylation and Platelet Function

Given the role of PAR4 in platelet activation, we wanted to test whether smoking-induced hypomethylation could impact on thrombus formation by altering platelet reactivity. In this experiment, we attempted to isolate the impact of DNA methylation at *F2RL3* from the broader genome-wide methylation signature of smoking, as well as any potential confounding factors. A phenotype-based recall experiment was undertaken in 41 healthy nonsmokers recruited from the ARIES (Accessible Resource for Integrated Epigenomic Studies) sub-study^25^ of the ALSPAC.^26–28^ To maximize power for a given sample size, participants were recruited from a pool of 200 individuals selected for invitation based on (previously measured) methylation extent at the *F2RL3* CpG site cg03636183 (CpG_3), with those at the extreme ends of the distribution being chosen. Participants were therefore recruited to one of two study arms - the low methylation group (N=19) or the high methylation group (N=22); these groups formed the basis of all subsequent by-group analyses. Full details of the selection and recruitment procedure are provided in Methods (ii) in the Supplemental Material. All those eligible for invitation were of European ancestry, as determined by comparison of genome-wide genetic data to HapMap II reference populations during genotyping quality control (for more details, see Cohort information in the Supplemental Material).

Recruited individuals provided fresh blood samples that were immediately analyzed for platelet reactivity, as assessed by stimulating platelet-rich plasma with different concentrations of AYPGKF peptide, a specific agonist of PAR4. Platelet responses were measured using flow cytometry to detect the open conformation of the platelet α_IIb_β_3_ integrin and platelet surface exposure of P-selectin, both markers of platelet activation during hemostasis. To assess the specificity of any differences observed, the same measurements were made after platelet-rich plasma was treated with SFLLRN, a PAR1 specific agonist. In addition, standard hematologic parameters were assessed and platelet surface receptor expression was measured. Finally, DNA was extracted from leukocytes and targeted pyrosequencing of *F2RL3* performed to capture the 4 CpG sites described previously (Figure 1B). Full details of the laboratory procedures are described in Methods (ii) in the Supplemental Material.

Previous studies have shown that the single nucleotide polymorphism, rs773902 (Figure 1B), is associated with regulation of platelet function.^10,12^ Specifically, the A-allele at the locus which encodes threonine (Thr) at residue 120 in transmembrane domain 2 (as opposed to alanine [Ala]) was associated with greater PAR4-induced human platelet aggregation and a higher level of Ca^+^ flux.^10^ We used full ARIES data together with existing genetic data in the ALSPAC cohort (details of the genotyping procedure conducted in the cohort are included in Cohort information in the Supplemental Material) to evaluate the relationship between rs773902 (A/G) genotype and DNA methylation at CpG_3 (cg03636183; N=731). In a series of post hoc exploratory analyses, we examined the properties of this genetic variant specifically within this recall sample (N=41).

##### Statistics

The by-group (high versus low methylation group) comparisons of continuous traits described below were conducted by 2-sample, 2-sided *t* test assuming equal variances (assumption of equal variances tested using a variance ratio test) unless the trait distribution did not approximate a normal distribution (based on a Shapiro-Wilk W-statistic <0.90) in which case, a 2-sample Wilcoxon rank-sum (Mann-Whitney) test was conducted. By-group comparisons of categorical traits were based on a Pearson χ^2^ test.

A by-group comparison of DNA methylation (measured by pyrosequencing) at each of the 4 CpG sites measured was done by *t* test. For assessing α_IIb_β_3_ integrin activation and P-selectin exposure, dose response curves of activation responses versus PAR4 (PAR1) agonist concentration were obtained from nonlinear regression of log[agonist concentration] versus response performed using GraphPad Prism (version 7.00 for Windows, GraphPad Software, La Jolla California USA, www.graphpad.com). By-group comparisons of dose response curves were then performed by 2-way ANOVA. By-group comparisons of α_IIb_β_3_ integrin activation and P-selectin exposure half maximum effective concentrations (EC_50_) and maximum response values after PAR4 (PAR1) activation and measured platelet surface receptors were compared by *t* test after the removal of outliers identified on the basis of a robust regression of outcome on DNA methylation group (high/low). Details of the analysis of potential confounders and exploratory analysis relating to rs773902 are provided in Methods (ii) in the Supplemental Material.

#### In Vitro Association Between Smoking and F2RL3 DNA Hypomethylation and Expression

To explore the biological response of *F2RL3* to cigarette smoke exposure at the cellular level, we used a previously established in vitro model.^29,30^ The model was applied to a human megakaryocytic cell lineage (the acute megakaryocytic leukemia cell line [CMK]),^31^ chosen because of the role of megakaryocytes as precursors to platelets and as the source of platelet mRNA and translational machinery.^32,33^ CMK cells were exposed to 4 doses of cigarette smoke extract (CSE) over the course of 4 days, with cells then harvested on day 5 and *F2RL3* DNA methylation, mRNA expression and PAR4 protein expression measured. mRNA expression was also measured in the endogenous control, RPLP0 (ribosomal protein lateral stalk subunit P0), both in the presence and absence of CSE exposure. Given challenges around PAR4 protein quantification using Western blotting-based approaches, relative protein abundance in CSE treated versus untreated CMK cells was assessed by tandem mass tag mass spectrometry, a technique that delivers global protein expression data. Details of quality control procedures applied in all experiments described are provided in Methods (iii) in the Supplemental Material.

As a comparator, the same model was also applied to human coronary artery endothelial cells^34^ (see Methods [iii] in the Supplemental Material). In this cell line, the impact of global demethylation (by 5-Aza application) on mRNA expression was also assessed.

##### Statistics

Analyses of methylation and mRNA expression data were conducted in Stata (version 16.1).^24^ Average *F2RL3* DNA methylation levels (%) at each of the 4 sites in CSE-exposed cells (n=4) were compared with levels in unexposed controls (n=4) using 2 sample 2-sided *t* tests assuming equal variances. Ratios of *F2RL3/RPLP0* mRNA levels in CSE-exposed cells (n=5) compared with unexposed controls (n=5) were natural log transformed and an average taken. The average log ratio for CSE-exposed cells was compared with the value in unexposed controls (ie, baseline) using a 2-sample 2-sided *t* test assuming equal variances. For plotting, fold change was calculated as the value in treated cells divided by the value in untreated cells. Statistical analyses of tandem mass tag-derived protein abundance data were focused on our target protein, PAR4, and our control protein, RPLP0; however, data for all proteins are included in Source Data (see Data Access Statement). Mean protein abundance following CSE exposure (n=3) as compared with control (n=3) was compared by 2-sample 2-sided *t* test assuming equal variances. For plotting, fold change was calculated as the abundance in treated cells divided by the abundance in untreated cells. In all the above analyses, the assumption of equal variances was tested using a variance ratio test.

#### Functional Regulation of F2RL3

To investigate the potential mechanism by which differential methylation could impact on *F2RL3* gene expression, a series of reporter constructs containing different fragments of *F2RL3* to drive expression of luciferase and a chromatin immunoprecipitation-based experiment were used. This analysis is described in full in Methods (iv) in the Supplemental Material (including statistics). In addition, data from the BLUEPRINT Consortium^35^ were used to explore the likely specificity of *F2RL3* regulatory control to megakaryocytes. Specifically, data on gene and transcript expression, hyper and hypo methylated regions, chromatin accessibility (DNAseI-Seq) and histone marks binding activity for *F2RL3* were extracted for cord blood-derived (untreated) megakaryocytes (CD34-negative, CD41-positive, CD42-positive megakaryocyte cell, CL:0002026) and qualitatively compared with results from other cell types.

### Study Approval

Details of the ethics approvals relevant to the ALSPAC cohort in general can be found on the study website (http://www.bristol.ac.uk/alspac/researchers/research-ethics/). Ethical approval for the ALSPAC study was obtained from the ALSPAC Ethics and Law Committee and the NHS South West—Frenchay Research Ethics Committee (REC reference 14/SW/1099) and all participants gave written informed consent. The Copenhagen City Heart Study was conducted according to the Declaration of Helsinki, all individuals gave written informed consent, and it was approved by a Danish ethical committee (KF100.2039/91).

## Results

### Smoking, DNA Hypomethylation, and Myocardial Infarction

We used observational data from the Copenhagen City Heart Study to evaluate the relationship between smoking, *F2RL3* DNA methylation and cardiovascular diseases specifically involving platelet-dependent arterial thrombosis, that is, myocardial infarction. Participants selected for inclusion in this study (N=4292) were broadly representative of the full baseline cohort (N=9234) with the expected enrichment for myocardial infarction events and corresponding shift in associated covariates (Table S1). After quality control procedures were implemented, complete methylation data (ie, a measure at all 4 CpG sites assayed) were available for 3205 participants and subsequent analyses were conducted using these data. The average age (SD) of participants at the time of sample collection was 65.5 (11.0) years and 45% were male; the majority were current (50%) or former (33%) smokers. Percentage *F2RL3* DNA methylation at all 4 sites was approximately normally distributed (Shapiro-Wilk W-statistics: CpG_1=0.95, CpG_2=0.87, CpG_3=0.94, CpG_4=0.95). DNA methylation was highly correlated in current smokers (pairwise correlations all *r*>0.77) and former smokers (*r*, 0.58–0.76) and was moderately correlated in those who had never smoked (r, 0.28–0.56; Figure S1).

**Figure 2.**
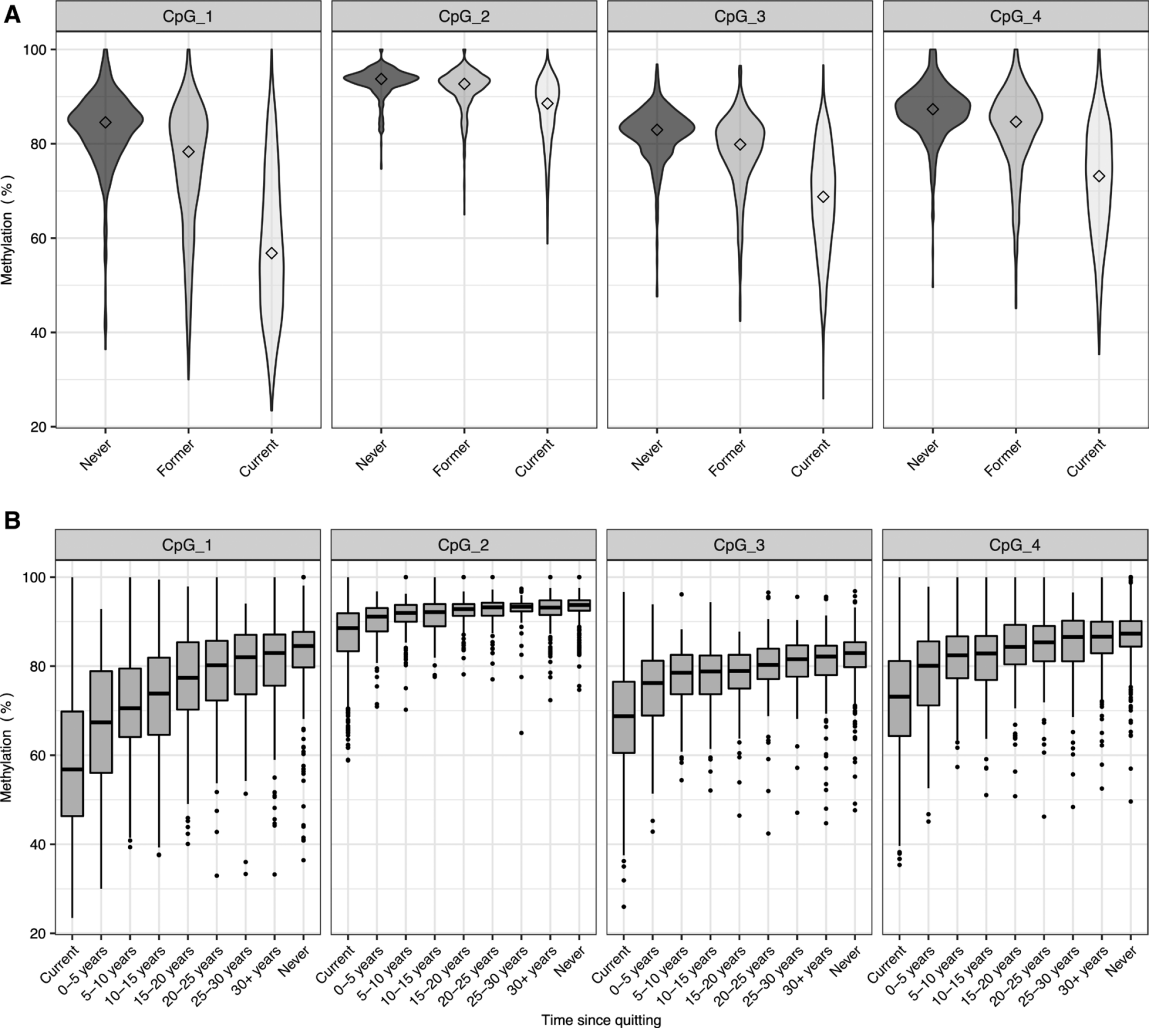
**DNA methylation extent (%) at F2RL3 by smoking status in selected participants from the Copenhagen City Heart Study (N=3205).** **A**, Violin plot of DNA methylation extent (%) at each cytosine–phosphate–guanine (CpG) of *F2RL3* according to smoking status at the time of sampling. Median values are marked by diamonds. N for each group is as follows: never=548; former=1068; current=1589. Results from a linear regression of DNA methylation on smoking group, adjusted for age and sex, are given in Table S2A; in all cases, *P*<6.0×10^−05^. **B**, Boxplot of DNA methylation extent (%) at each CpG of *F2RL3* by time since quitting smoking in former smokers. DNA methylation extent is also presented for current smokers and never smokers. N for each group is as follows: Current: 1589, 0–5 y: 135, 5–10 y: 127, 10–15 y: 127: 15–20 y: 109, 20–25 y: 99, 25–30 y: 71, 30+ years: 203, never: 548. Upper and lower hinges of boxplots correspond to the first and third quartiles with the centre line indicating the median and whiskers extending from the hinge to the largest (smallest) value no further than 1.5×interquartile range from the hinge. In a linear regression model fitted on data restricted to former smokers, the estimated change in *F2RL3* DNA methylation per additional year since smoking cessation (adjusting for age and sex) was 0.42 (95% CI, 0.35–0.48), 0.08 (95% CI, 0.06–0.10), 0.18 (95% CI, 0.14–0.22), and 0.23 (95% CI, 0.19–0.27), for CpG sites 1 to 4, respectively (Table S2B).

Observationally, smoking status was associated with DNA hypomethylation of *F2RL3* across all 4 CpG sites with the strongest association being seen at CpG_1 at which the mean difference in percentage DNA methylation in former and current smokers as compared with never smokers after adjusting for age and sex was −7.15% (95% CI, −8.58 to −5.71; *P*=4.1×10^−22^) and −23.98% (95% CI, −25.33 to −22.62; *P*<1.0×10^−100^), respectively. At this site, together with age and sex, smoking status explained 36% of the variation in DNA methylation (Figure 2A, Table S2A). Among smokers, there was a 5.53% (95% CI, 5.07–5.99; *P*<1.0×10^−100^) reduction in methylation at CpG_1 per doubling of pack years (Table S2A). DNA methylation at *AHRR* captured the most variation in *F2RL3* DNA methylation making it a useful proxy for cigarette smoke exposure in this sample. The categorization of former smokers according to time since giving up revealed a gradual recovery of methylation levels to those seen in people who reported never having smoked (Figure 2B). In former smokers, time since cessation explained an estimated 4% (CpG_2) to 13% (CpG_1) of the variance in *F2RL3* DNA methylation with an increase in methylation at CpG_1 of 0.42% (95% CI, 0.35–0.48; *P*=6.1×10^−36^) for each additional year since cessation after adjustment for sex and age (Table S2B).

Having characterized the relationship between smoking and *F2RL3* DNA methylation, 207 prevalent myocardial infarction cases (ie, those whose event occurred before samples were collected) were excluded, leaving 2998 individuals (648 cases and 2350 controls) for subsequent analyses (Table 1) with an estimated mean (SD) time from baseline to myocardial infarction event of 9.0 (5.5) years. We estimated association of the extent of *F2RL3* DNA methylation at CpG sites CpG_1 to CpG_4, with risk of incident myocardial infarction in a stratified analysis within smoking status groups (current, former, and never smokers). The strongest association was seen at CpG_1 where in a sex- and age-adjusted model, the estimated hazard ratio of incident myocardial infarction in current and former smokers was 1.29 (95% CI, 1.15–1.44; *P*=2.1×10^−5^) and 1.38 (95% CI, 1.15–1.65; *P*=5.5×10^−4^), respectively, per SD decrease in *F2RL3* DNA methylation (Table 2). These associations persisted both after adjustment for exposure to cigarette smoke, as proxied by *AHRR* DNA methylation, and after additional adjustment for other known correlates of myocardial infarction incidence (Table 2). No strong evidence for an association between *F2RL3* DNA methylation and myocardial infarction was seen in the never smoking group. Association results for the fully adjusted model across all CpG sites are in Table S3.

**Table 1.**
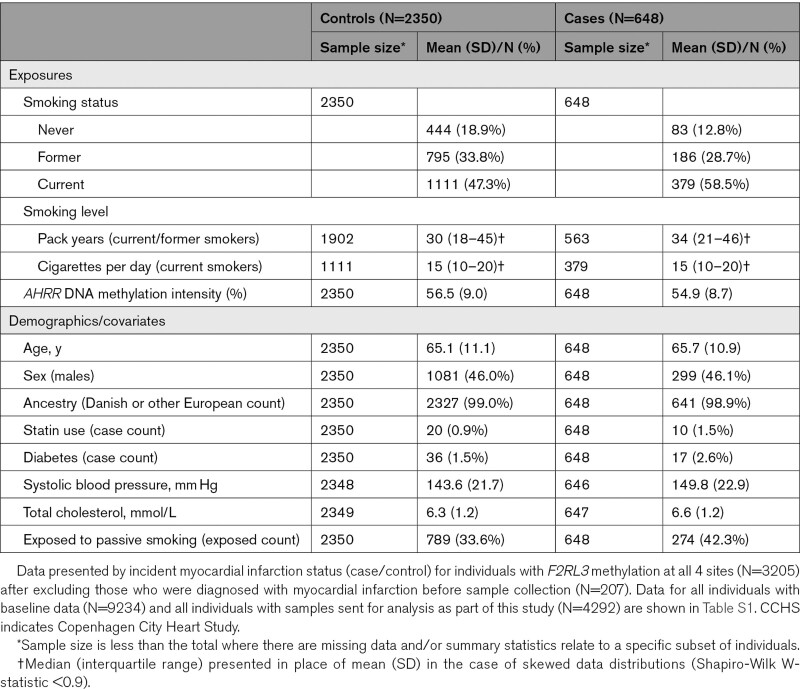
Characteristics of CCHS Population (N=2998)

**Table 2.**
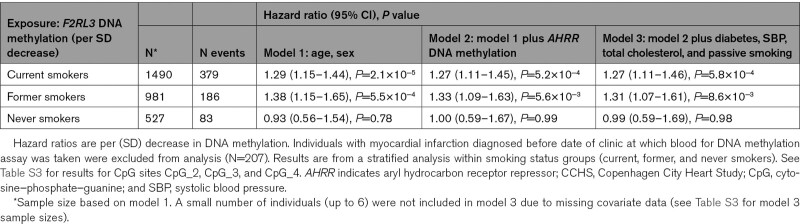
Associations Between *F2RL3* DNA Methylation Extent at CpG_1 and Myocardial Infarction in the CCHS (N=2998)

Similar patterns were observed for all-cause mortality in myocardial infarction cases. A HR of 1.31 (95% CI, 1.10–1.56, *P*=0.003) per SD decrease in *F2RL3* DNA methylation at CpG_1 in current smokers (from the fully adjusted model) suggests that observationally, *F2RL3* DNA methylation is associated with outcome following a myocardial infarction in this subgroup (Table S4A). Associations were in a consistent direction but less strong for other subgroups (former and never smokers) based on the same fully adjusted model (Table S4A) and across the other CpG sites (Table S4B). We found no strong evidence of collider bias of any appreciable magnitude affecting our results (Results (i) in the Supplemental Material and Figure S2).

Finally, mediation analyses performed in ever (current and former) smokers suggested that in our sample, DNA methylation at *F2RL3* mediated an estimated 34% and 39% of the smoking effect on increased risk of myocardial infarction and death after myocardial infarction (after having adjusted for covariates), respectively (Table S5). This analysis was only performed for CpG_1, the CpG with the strongest association with both smoking and the primary outcome, myocardial infarction. By way of comparison, DNA methylation at *AHRR* mediated an estimated 16% and 18% of the smoking effect on increased risk of myocardial infarction and death after myocardial infarction, respectively (Table S5).

### Association Between DNA Hypomethylation and Platelet Function

Two hundred participants from ALSPAC were selected for recall based on previously measured *F2RL3* DNA methylation, 100 from the top end of the distribution of DNA methylation values (high methylation) and 100 from the bottom end (low methylation). Comparisons of age, sex and a number of other variables relevant to cardiovascular health showed that while those in the high methylation group were slightly older (23.2 years compared with 23.0 years) and less likely to carry the minor allele (A) at rs773902, all other measures were similar in the two groups (Table S6). Finally, 22 individuals were recruited from the top end of the distribution of DNA methylation values and 19 from the bottom end of the distribution. There was no strong evidence that any selection bias had occurred (Table S7).

**Figure 3.**
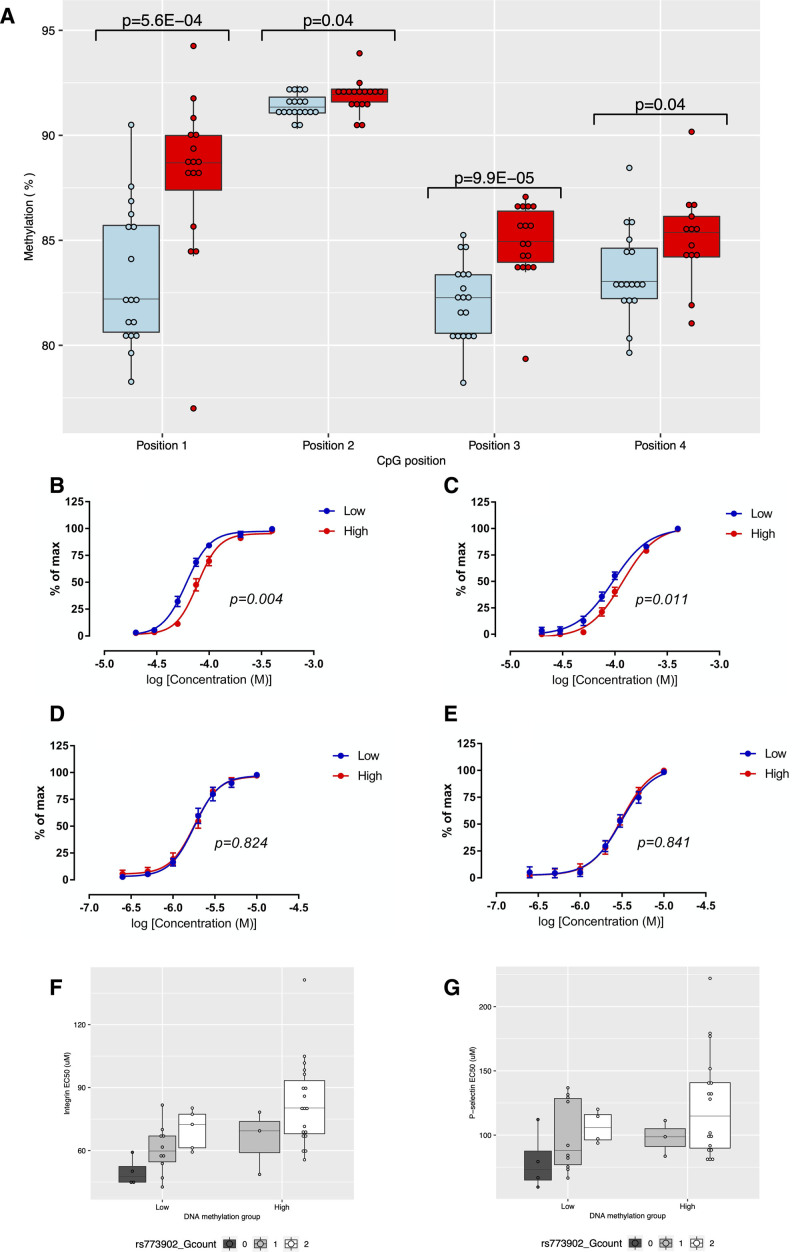
**Differential platelet reactivity between groups of Avon Longitudinal Study of Parents and Children (ALSPAC) participants selected based on low (blue, n=19) or high (red, n=22) DNA methylation during childhood and adolescence.** **A**, Boxplot showing the between group difference in DNA methylation extent (%) at the time of platelet reactivity assessment at 4 cytosine–phosphate–guanine (CpG) positions in *F2RL3* as assessed by 2-sample 2-sided *t* test (assuming equal variances). **B** and **C**, Levels of α_IIb_β_3_ integrin (**B**) and α-granule P-selectin exposure (**C**) following platelet stimulation with the PAR4 (protease-activated receptor 4)-specific agonist peptide, AYPGKF. *P* values derived from a by-group comparison of dose response curves performed by 2-way ANOVA. Points represent group means with 95% CIs; (**D** and **E**) Levels of α_IIb_β_3_ integrin (**D**) and α-granule P-selectin exposure (**E**) following platelet stimulation with the PAR1-specific agonist peptide, SFLLRN. *P* values derived from a by-group comparison of dose response curves performed by 2-way ANOVA. Points represent group means with 95% CIs; (**F** and **G**) Boxplot showing the between group difference in α_IIb_β_3_ integrin EC_50_ (**F**) and α-granule P-selectin EC_50_ (**G**) in response to PAR4-specific agonist peptide AYPGKF, by rs773902 genotype. In boxplots, upper and lower hinges of boxplots correspond to the first and third quartiles with the centre line indicating the median and whiskers extending from the hinge to the largest (smallest) value no further than 1.5×interquartile range from the hinge.

Analysis of the contemporary (pyrosequencing-based) methylation showed that the selection and recruitment process had successfully delivered 2 groups with a mean difference in DNA methylation extent at *F2RL3.* The correlation across positions ranged from 0.41 to 0.80 (Table S8). Of the 4 targeted *F2RL3* CpG sites, the largest mean difference (4.8%) in DNA methylation was observed at CpG_1 (Figure 3A; Table S9). The difference in methylation between the groups relative to the difference between smokers and nonsmokers can be seen in Figure S3. Dose response curves following stimulation by a PAR4-specific agonist differed for integrin activation (*P*=0.004) and P-selectin exposure (*P*=0.011; Figure 3B and 3C). Similarly, mean EC_50_ values for both measures were lower in the low DNA methylation group (Table S9). These results correspond to an increase in responsiveness with DNA hypomethylation. For example, at 75 µmol/L AYPGKF, the response in high methylation status individuals was 47.6% compared with 68.6% in the low methylation status individuals, for integrin activation. For P-selectin exposure, the equivalent responses were 21.1% versus 35.6%. No between-group differences were observed after the stimulation of platelets via a PAR1-specific agonist (Figure 3D and 3E; Table S9). We found no strong evidence of a difference in the expression of the individual components of integrin, CD41 (*P*=0.86) and CD61 (*P*=0.85), in basal (nonstimulated) control samples (Table S9).

There were no between-group differences in hematological measures or in other potential biological confounders of platelet function that could explain the difference seen in platelet reactivity (Table S10A). While there was some evidence for a difference in 2 technical confounders (time to assay and technician), when fitted in a linear model alongside methylation group, neither were associated with the outcomes of interest (ie, those platelet properties previously found to be associated with methylation group; Table S10B). Maternal education, expressed as a binary trait indicating those participants whose mothers had A level or degree level qualifications, was also weakly associated with methylation group (*P*=0.05; Table S10A). The minor allele (A) frequency of rs773902 was higher in the low methylation group (47.4%) as compared with the high methylation group (6.8%; Table S10A). While maternal education was associated with both integrin activation and P-selectin exposure after PAR4-specific stimulation, fitting maternal education alongside methylation group did not attenuate the methylation group effect in either case, suggesting an independent contribution (Table S10B). Fitting rs773902 genotype alongside methylation group in a linear model attenuated but did not extinguish the methylation group effect on both integrin activation EC_50_ and P-selectin exposure EC_50_ (after PAR4-specific stimulation; Table S10B). These results suggest that the effects of methylation and rs773902 on platelet activation via PAR4 are not independent, prompting further exploratory analyses.

The difference in minor allele frequency between the 2 study groups indicated a correlation between rs773902 genotype and *F2RL3* DNA methylation in ALSPAC. This was confirmed using data from the ARIES cohort (N=731), where the A-allele at rs773902 was associated with a 1.2% decrease (95% CI, 0.5–1.8, *P*=4.4×10^−4^) in methylation extent at CpG_3 (cg03636183; as measured between the ages of 15 and 18 years). The effect of genotype appears consistent in the 2 arms of the study (Figure 3F and 3G) and specific to PAR4 (Figure S4). Results of further exploratory analyses relating to rs773902 are described in Results (ii) in the Supplemental Material.

### In Vitro Association Between Smoking and *F2RL3* DNA Hypomethylation and Expression

**Figure 4.**
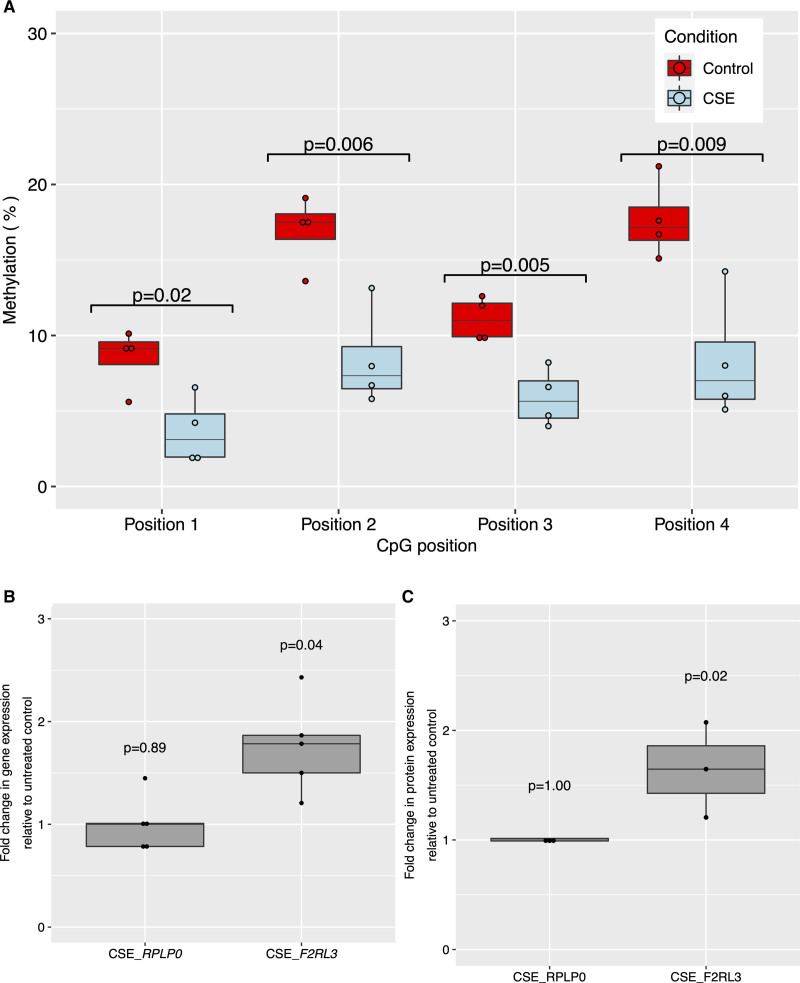
**Effect of cigarette smoke exposure (CSE) on *F2RL3* DNA methylation, mRNA expression and protein abundance in acute megakaryocytic leukemia (CMK) cells.** **A**, Boxplot showing between treatment difference in methylation %. Control=no treatment (n=4); CSE=exposure of cells to cigarette smoke extract (n=4). Test results are for a 2-sample 2-sided *t* test for a difference of means. **B**, Boxplot showing effect on gene expression of exposure of CMKs to 96 h of CSE compared with untreated control cells for *F2RL3* (n=5) and the endogenous control *RPLP0* (n=5). Test results are for a 2-sample 2-sided *t* test for a difference of means between CSE treated cells and untreated controls in each case. **C**, Boxplot showing effect on protein expression of exposure of CMKs to 96 h of CSE (n=3) compared with untreated control cells (n=3) for F2RL3/PAR4 and the endogenous control RPLP0. Test results are for a 2-sample 2-sided *t* test for a difference of means between CSE treated cells and untreated controls in each case. In boxplots, upper and lower hinges correspond to the first and third quartiles with the centre line indicating the median and whiskers extending from the hinge to the largest (smallest) value no further than 1.5×interquartile range from the hinge. Source data are provided as a Source Data file (see Data Access Statement).

Exposure of cells to CSE reduced *F2RL3* DNA methylation in CMK cells across all CpG sites (Figure 4A). This DNA hypomethylation was accompanied by a 1.7-(95% CI, 1.2–2.4, *P*=0.04) fold increase in *F2RL3* mRNA levels relative to untreated controls while expression of the endogenous control, *RPLP0*, was unchanged (Figure 4B). Corresponding results for the human coronary artery endothelial cells-based experiment are presented in Results (iii) in the Supplemental Material (including Figure S5) and show similar patterns of association. Relative protein quantification using tandem mass tag mass spectrometry applied to cell lysates showed a corresponding increase in PAR4 (1.6-fold increase in treated as compared with untreated cells based on three independent replicates, *P*=0.02 from 2-sample *t* test assuming equal variance) and no change in the previously defined RPLP0 (control) protein (Figure 4C). While our focus was on *F2RL3*/PAR4, this analysis provided data on over 6000 proteins and demonstrated a broad protein expression signature associated with cell exposure to CSE (Figure S6).

### Functional Regulation of *F2RL3*

Results of a series of reporter assays and a chromatin immunoprecipitation-based experiment indicate occupancy at a CEBP (CCAAT/enhancer binding protein) recognition sequence in exon 2 (see Figure 1B) as a possible mechanism by which methylation could impact on gene expression. Combining both the *F2RL3* promoter and the *F2RL3* exon 2 fragment in the pCpGL reporter vector (pCpGL_*F2RL3*pro_exon2) resulted in increased luciferase activity relative to the promotor only construct (Figure S7), suggesting that the exon 2 region has enhancer activity that acts with the endogenous *F2RL3* promoter. Mutation of the CEBP recognition sequence in exon 2 (pCpGL_*F2RL3*pro_exon2 CCAAT deletion) attenuated luciferase reporter gene activity (Figure S7). Subsequent chromatin immunoprecipitation-based experiments in human coronary artery endothelial cells cells showed increased occupancy of the *F2RL3* exon 2 CEBP recognition site with CEBP-β (a prototypical isoform abundantly expressed in hemopoietic tissue and endothelium^36^) following global demethylation with 5-Azacytidine. These results are described in more detail in Results (iv) in the Supplemental Material.

Data from BLUEPRINT suggests that *F2RL3* expression is enriched in megakaryocytes (Figure S8); expression of the full-length transcript (ENST00000248076.3) dominates over an alternate truncated transcript (ENST00000599210.1; Figure S9). Using megakaryocyte epigenetic data from BLUEPRINT, we see the 4 CpG sites we pyrosequenced sit in a hypomethylated region at the start of exon 2 with the remainder of this exon being designated hypermethylated. The region is characterized by histone H3 lysine 4 trimethylation (H3K4me3) marks, suggestive of a promotor region, and is designated a DNase hypersensitivity peak site (Figure 1B, Figure S10A) with comparisons across cell types suggesting greater chromatin accessibility in megakaryocytes as compared with other lineages (Figure S10B).

## Discussion

Multiple studies have observed associations between differential DNA methylation at CpG sites across the genome and exposure to cigarette smoke.^1–5^ However, little has been done to establish biological mechanisms that could link cigarette smoking to epigenetic regulation and ultimately to risk of myocardial infarction morbidity and mortality. Understanding the mechanisms behind these events is important when attempting to improve risk stratification and for therapy development. With a focus on DNA hypomethylation at *F2RL3*, we have triangulated evidence from independent sources and scientific disciplines to test the hypothesis that epigenetic DNA hypomethylation at *F2RL3* associated with smoking increases risk of pathological thrombosis via a PAR4-induced increase in platelet reactivity.

Under the assumption that differential DNA methylation at *F2RL3* can influence platelet reactivity via modulation of PAR4, we might expect to see an association between DNA methylation and the incidence of platelet-dependent arterial thrombosis leading to myocardial infarction in the population. In selected individuals from a cohort study, we were able to confirm the known relationship between smoking and methylation at this locus. We showed that in current smokers DNA hypomethylation at *F2RL3* was associated with a 27% increase in risk of myocardial infarction and a 38% increase in the risk of death after a myocardial infarction. Furthermore, mediation analyses suggested the specific contribution of *F2RL3* DNA methylation to the association between smoking and myocardial infarction to be in the region of 34%. However, due to potential confounding, reverse causation and other biases, these observational associations cannot necessarily be assumed to be causal and the extent to which the risk estimates generated in this selected group of individuals can be extrapolated to a random, unselected population sample is not clear.

Building on this, we considered whether altered platelet function could serve as a mechanism by which *F2RL3* hypomethylation is related to thrombotic disease. Individuals were selected for invite based on having low or high DNA methylation at *F2RL3* according to previously measured (by Illumina array) methylation. Although the pyrosequencing assay used to assess contemporary methylation (ie, methylation at the time of the platelet experiment) returned methylation values higher than those recorded at the same site by the Illumina Infinium assay, we were able to verify that there was a mean difference in methylation extent (in the expected direction) in those subsequently recruited to the 2 arms of the study. Evidence from the literature suggests that while it is not uncommon for there to be between-platform differences of this magnitude,^37^ bisulfite pyrosequencing tends to perform well when compared with alternative approaches.^38^ Blood sample collection in a group of healthy nonsmoking young adults selected in this way enabled us to evaluate the biological consequences of variation in DNA methylation at *F2RL3* independent of the broader effects of smoking and its correlates. In this context, we observed an association between methylation group and platelet reactivity in response to PAR4 stimulation. The lack of response following activation of the highly related PAR1 receptor suggests a high degree of selectivity in the underlying mechanism. The isolation of variation in *F2RL3* DNA methylation from other pathways modulated by smoking as done here is important given the global nature of the effect of smoking on the methylome,^4^ transcriptome,^39^ and proteome (as evidenced by the tandem mass tag analysis presented in our in vitro work).

In vitro experiments using a cardiovascular disease-relevant cell line (CMK cells) gave evidence of differential DNA methylation, mRNA expression and protein abundance at *F2RL3* following exposure to aqueous CSE. While limitations to the study design meant that we were unable to directly evaluate the role of *F2RL3* methylation as a mediator of the treatment effect on mRNA expression and protein abundance, both epigenetic annotations in the region (based on public data sets) and supplementary functional analysis that we conducted suggest the presence of relevant genetic machinery within *F2RL3* exon 2. Specifically, a potentially methylation-sensitive CCAAT binding site was identified, deletion of which resulted in attenuated luciferase reporter gene activity. CEBP binding to an identical CCAAT recognition site at a different locus (*MLH1*), is known to be reduced by DNA methylation of a CpG residue in an identical relative position to the CCCAT recognition sequence to that observed with CpG_1.^40^ Furthermore, global demethylation increased occupancy of the CEBP recognition site with CEBP-β (a prototypical isoform abundantly expressed in hemopoietic tissue and endothelium^36^). These findings suggest that *F2RL3* expression could be in part constrained by constitutive DNA methylation of CpG sites within an exon 2 enhancer, with methylation reducing CEBP-β occupancy and enhancer activity—smoking disturbs this regulation, reducing methylation and increasing CEBP-β occupancy and in turn *F2RL3* expression. Evidence in support of this hypothesis comes from a recent transcriptome-wide association study that revealed an association between *F2RL3* expression assessed in lymphoblastoid cell and smoking (n=92 current versus n=364 never smokers).^41^ However, no relationship between *F2RL3* expression and mortality was observed^41^ and the same association was not seen in a similar study based on whole blood gene expression (n=1421 current versus n=4860 never smokers).^42^

Combined evidence here not only identifies *F2RL3* DNA methylation as a possible contributory pathway from smoking to disease risk, but from any feature potentially influencing *F2RL3* regulation in a similar manner. It has been demonstrated that a relatively large proportion of the variation observed in DNA methylation across the genome arises from genetic variation,^43^ in particular *cis*-acting loci located close to the DNA methylation site they control.^44^ Results from a recent meta-genome-wide association study of methylation conducted by the Genetics of DNA Methylation Consortium^45^ (http://mqtldb.godmc.org.uk/index, accessed on 7 April, 2021) suggest over 100 genetic variants in and around *F2RL3* are associated with methylation at cg03636183 (our CpG_3), including rs773902. Building on this evidence, exploratory analyses conducted here suggest overlapping contributions to PAR4-stimulated platelet reactivity from methylation and rs773902 genotype. In a multivariable model, rs773902 genotype explained more of the variation in integrin and P-selectin than the methylation group. However, such comparisons across different measures are difficult to interpret and may reflect greater measurement error in methylation as compared with genotype. It is also possible that rs773902 is pleiotropic, having both a direct and an indirect (via methylation) effect on platelet function. There is currently incomplete evidence concerning whether or not the difference in platelet reactivity seen with rs773902 genotype can be attributed to changes in PAR4 expression levels.^10,12^ Publicly available eQTL (expression quantitative trait loci) data derived from selected tissues (GTEx,^46^ Table S12) and blood (eQTLGen Consortium,^47^ Table S13) provide some evidence for an association between rs773902 (and nearby variants) and *F2RL3* expression, but this is incomplete with one study of platelet-specific eQTLs not showing an association between rs773902 and *F2RL3* expression (N=290, *P*=0.27) and an analysis of megakaryocytes showing only weak evidence of association (N=185, *P*=0.05).^48^ Ascertainment of the relative contributions of DNA sequence variation versus methylation differences to PAR4 functionality is further complicated by the existence of an alternative *F2RL3* transcript generated by the premature splicing of exon 2 leading to truncation just short of rs773902, rendering that position a 3′UTR in the alternate isoform. It appears that there are overlapping though possibly additional independent contributions to platelet function here, but further evidence is needed to fully understand the regulatory machinery at *F2RL3*, including the potential for interactions between modifiable exposures, such as smoking, and genotype. Such work could usefully follow the approach of Rodriguez et al^49^ who were able to demonstrate that disruption of platelet *GRK5* expression by rs10886430-G caused enhanced platelet reactivity specifically via PAR4 receptor signaling.

The strength of this work comes from evidence alignment across multiple experiments, each with independent limitations and biases—such alignment is hard to rationalize in the absence of true biological effect. However, more needs to be done to fully understand the DNA methylation related risk differences potentially driven by this locus including, crucially, whether the associations we see here reflect causal mechanisms. Importantly, the development of PAR4 antagonists as novel antithrombotics do provide an attractive target intervention^50^ and such therapies appear to have the additional advantage of lower bleeding risk compared with antiplatelet drugs that target other receptors.^51–53^ However, while BMS-986120, an orally active, selective, and reversible PAR4 antagonist registered by Bristol-Myers Squibb has shown potential in preclinical and early phase trials,^53,54^ it does not appear to have progressed beyond these early phase trials. Meanwhile, French et al^55^ added a function-blocking PAR4 antibody to the list of potential PAR4-targeting antithrombotic therapies. The gravity of our findings are heightened by our growing understanding of the temporal nature of DNA methylation marks. It has been suggested that it can take many years for DNA methylation levels to recover in smokers after cessation^2,56,57^ and in our own collections, it is evident that smoking-induced DNA hypomethylation of the *F2RL3* CpG sites persists for decades (Figure 2B). In this context, even modest health implications of differential methylation at *F2RL3* (induced by any number of natural or exposure events) are arguably important in light of life course risk and should be explored further in efforts to reduce unnecessary burden of myocardial infarction morbidity and mortality.

In conclusion, epigenetic regulation at *F2RL3*, specifically DNA hypomethylation in response to smoking (and potentially other exposure events), may increase platelet reactivity through PAR4 function, providing a route to increased risk of myocardial infarction morbidity and mortality. If verified, these findings have implications for the utility of recently approved drugs designed to target PAR4.

## Article Information

### Acknowledgments

We would like to acknowledge the following people from University of Bristol for their contribution to this work: Elizabeth Aitken for her contribution to the laboratory work which is presented in the In vitro association between smoking and *F2RL3* DNA hypomethylation and expression section of the methods; Ana Goncalves Soares for advice regarding statistical analysis; David Hughes for his contribution to figure generation. We are grateful to the Bristol Proteomics Facility for assistance with this work, and the expertise provided by Dr Kate Heesom and Dr Phil Lewis. This study makes use of data generated by the Blueprint Consortium. A full list of the investigators who contributed to the generation of the data is available from www.blueprint-epigenome.eu. Funding for the project was provided by the European Union’s Seventh Framework Programme (FP7/2007- 2013) under grant agreement no. 282510 - BLUEPRINT. Avon Longitudinal Study of Parents and Children (ALSPAC): We are extremely grateful to all the families who took part in this study, the midwives for their help in recruiting them and the whole ALSPAC team, which includes interviewers, computer and laboratory technicians, clerical workers, research scientists, volunteers, managers, receptionists and nurses. The UK Medical Research Council and the Wellcome Trust (Grant ref: 217065/Z/19/Z) and the University of Bristol provide core support for ALSPAC. Copenhagen City Heart Study: We acknowledge participants and team of the Copenhagen City Heart Study. The Danish Heart Foundation and the Capital Region of Denmark supported the Copenhagen City Heart Study.

### Sources of Funding

This work was specifically supported by the Medical Research Council (MRC) Integrative Epidemiology Unit (IEU; MC_UU_12013/3). N.J. Timpson is a Wellcome Trust (WT) Investigator (202802/Z/16/Z), is the PI of the Avon Longitudinal Study of Parents and Children (MRC and WT 217065/Z/19/Z), is supported by the University of Bristol NIHR Biomedical Research Centre (BRC-1215-2001), the MRC Integrative Epidemiology Unit (MC_UU_00011/1) and works within the CRUK Integrative Cancer Epidemiology Programme (C18281/A29019). N.J. Timpson, L.J. Corbin, A.E. Taylor, C. Relton, and G. Davey Smith work in the MRC IEU at the University of Bristol which is supported by the MRC (MC_UU_00011/1, MC_UU_00011/5) and the University of Bristol. C. Relton is supported by the Cancer Research UK (CRUK) Integrative Cancer Epidemiology Programme (C18281/A29019). G. Davey Smith and A.W. Poole are funded by the British Heart Foundation (BHF; AA/18/1/34219). A.W. Poole is a Wellcome Trust Investigator (219472/Z/19/Z). K. Taylor is a BHF funded PhD student (FS/17/60/33474). This study was supported by the NIHR Biomedical Research Centre at the University Hospitals Bristol National Health Service (NHS) Foundation Trust and the University of Bristol. The views expressed in this publication are those of the author(s) and not necessarily those of the NHS, the NIHR or the Department of Health. The study was supported by the NIHR Bristol Biomedical Research Unit in Cardiovascular Medicine and by BHF, grants PG/11/44/28972, FS/12/77/29887 and CH95/001. Funding was also provided by programme and project support from the BHF to AWP (RG/15/16/31758, PG/15/96/31854, PG/13/14/30023). This research was funded in whole, or in part, by the Wellcome Trust [202802/Z/16/Z]. For the purpose of Open Access, the author has applied a CC BY public copyright licence to any Author Accepted Manuscript version arising from this submission.

### Disclosures

None.

### Supplemental Materials

Expanded Methods

Expanded Results

Figures S1–S10

Tables S1–S13

## Supplementary Material



## Novelty and Significance

### What Is Known?

- Cigarette smoking is reliably associated both with increased risk of atherosclerotic cardiovascular disease and with DNA hypomethylation at *F2RL3* (F2R like thrombin or trypsin receptor 3).
- DNA hypomethylation at *F2RL3* has been associated with cardiovascular/heart disease in observational studies.
- *F2RL3* codes for PAR4 (protease-activated receptor 4), a protein that activates platelets in response to thrombin generated at the site of tissue injury.
