# Supplementary figures and images for: Epigenetic Regulation of F2RL3 Associates With Myocardial Infarction and Platelet Function
Source: Circ Res. 2022 Jan 6;130(3):384–400. doi: 10.1161/CIRCRESAHA.121.318836 (PMC8812435; doi:10.1161/CIRCRESAHA.121.318836)

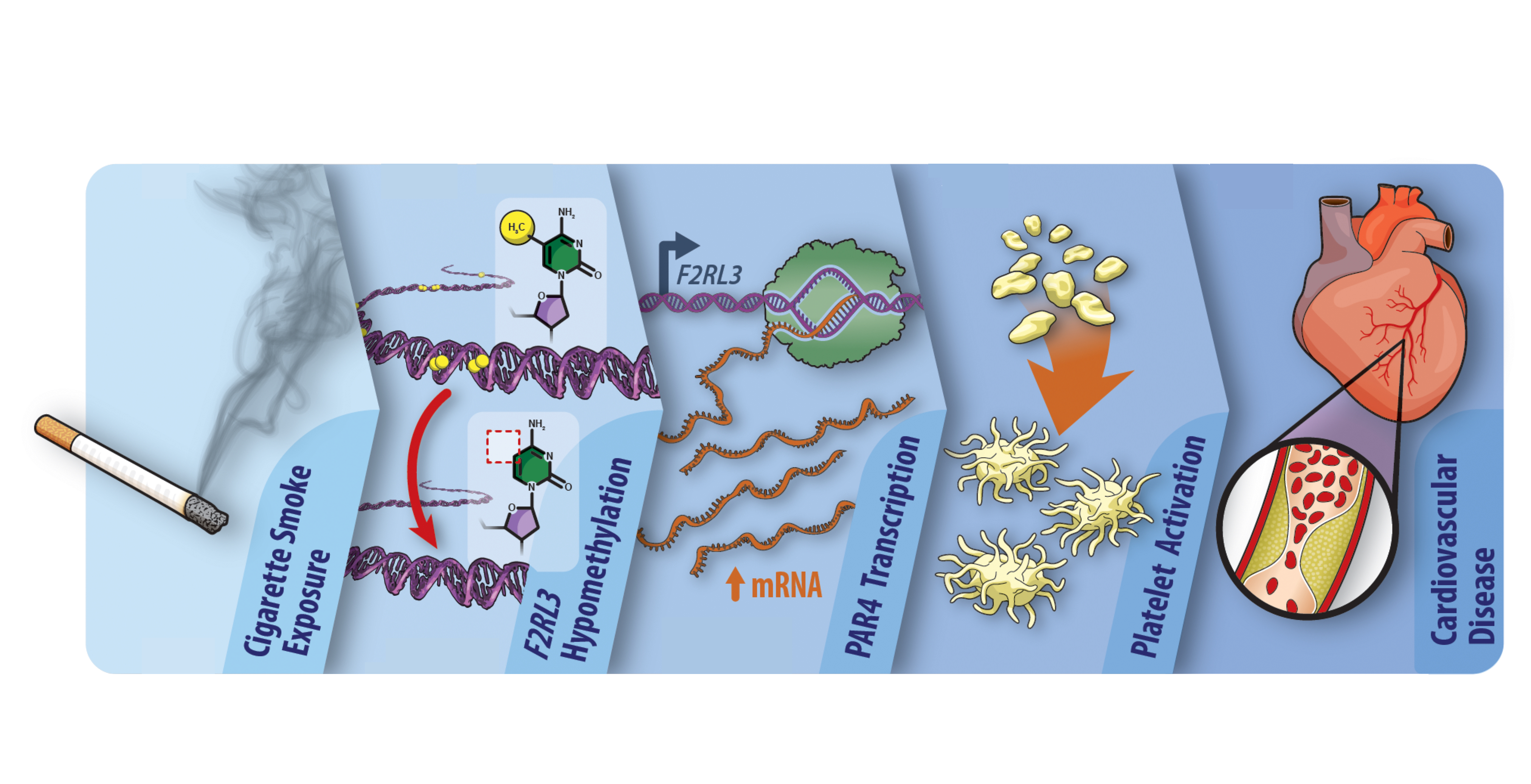

Supplement: Supplementary file 1 [file res-130-384-s001.jpg]
