# Supplementary material for: Epigenetic Regulation of F2RL3 Associates With Myocardial Infarction and Platelet Function
Source: Circ Res. 2022 Jan 6;130(3):384–400. doi: 10.1161/CIRCRESAHA.121.318836 (PMC8812435; doi:10.1161/CIRCRESAHA.121.318836)
Supplement: Supplementary file 2 [file res-130-384-s002.pdf]

Supplemental Material for:

Epigenetic regulation of *F2RL3* associates with myocardial infarction and platelet function

Table of Contents

Supplementary Methods ... ***5***

(i) Smoking, DNA hypomethylation and myocardial infarction ... **5**

Sample ... 5
F2RL3 DNA methylation ... 6
AHRR DNA methylation ... 8
Collider bias analysis ... 9
Extended statistics ... 9

(ii) Association between DNA hypomethylation and platelet function ... **10**

Study Design ... 10
Ethical considerations and informed consent ... 11
Participant recruitment ... 11
Sample Collection ... 12
Sample analysis ... 13
F2RL3 DNA methylation ... 14
Measured confounders ... 14
Extended statistics ... 16

(iii) *In vitro* association between smoking and *F2RL3* DNA methylation and expression ... **16**

Cell culture ... 16
F2RL3 DNA methylation ... 17
F2RL3 mRNA ... 18
Tandem mass tagging (TMT) protein quantification (CMK cells only) ... 18
Global DNA demethylation with 5-Azacytidine (HCAEC cells only) ... 20
Extended statistics ... 21

(iv) Functional regulation of *F2RL3* ... **21**

Reporter assay ... 21
Statistics ... 25
Chromatin immunoprecipitation (ChIP) in human coronary artery endothelial cells (HCAEC) ... 25

Cohort Information ... **27**

The Avon Longitudinal Study of Parents and Children (ALSPAC) ... 27

Supplementary Results ... ***31***

(i) Smoking, DNA hypomethylation and myocardial infarction ... **31**

Collider bias analysis ... 31

(ii) Association between DNA hypomethylation and platelet function ... **31**

Results of exploratory analyses relating to rs773902 ... 31

(iii) *In vitro* association between smoking and *F2RL3* DNA hypomethylation and expression ... **32**

Human coronary artery endothelial cell results ... 32

(iv) Functional regulation of *F2RL3* ... **32**

Results of reporter assay ... 32
Chromatin immunoprecipitation (ChIP) in human coronary artery endothelial cells (HCAEC) results ... 32

Supplementary Figures ... ***34***

Supplementary Figure 1. The Pearson correlation between the extent of DNA methylation at CpG_1 to CpG_4 (N=3,205) ... **34**

Supplementary Figure 2. Results from an assessment of collider bias and its potential impact on the case-only analysis ... **35**

Supplementary Figure 3. Difference in mean methylation extent (%) between low (blue) and high (red) methylation groups in the recall study relative to the distribution of methylation extent in smokers, former smokers and never smokers 37

Supplementary Figure 4. Within methylation group effect of rs773902 genotype on PAR1-stimulated platelet reactivity measures 38

Supplementary Figure 5. Effect of cigarette smoke exposure (CSE) on *F2RL3* DNA methylation and mRNA expression in human coronary artery endothelial cells (HCAEC) 39

Supplementary Figure 6. Volcano plot showing differential protein abundance in CSE-treated cells compared to untreated control cells. 40

Supplementary Figure 7. Enhancer activity of the *F2RL3* exon 2 region in HEK-293 cells. 41

Supplementary Figure 8. RNA-Seq data showing gene expression of *F2RL3* in megakaryocyte cells as compared to other cell types. 42

Supplementary Figure 9. RNA-Seq data showing transcript expression of *F2RL3* in megakaryocyte cells. 43

Supplementary Figure 10A. UCSC browser view of epigenetic annotations of *F2RL3* 44

Supplementary Figure 10B Chromatin accessibility (DNAseI-Seq) at *F2RL3* in megakaryocytes as compared to other cell lineages 45

Supplementary Tables *46*

Supplementary Table 1. Characteristics of study population in the Copenhagen City Heart Study 46

Supplementary Table 2A. Associations between smoking and *F2RL3* DNA methylation in the Copenhagen City Heart Study (N=3,205) 47

Supplementary Table 2B. Associations between time since cessation and *F2RL3* DNA methylation in former smokers in the Copenhagen City Heart Study (N=913) 48

Supplementary Table 3. Associations between *F2RL3* DNA methylation extent and myocardial infarction in the Copenhagen City Heart Study (N=2,998) 49

Supplementary Table 4A. Associations between *F2RL3* DNA methylation extent at CpG_1 and mortality in individuals experiencing a myocardial infarction in the Copenhagen City Heart Study (N=648) 50

Supplementary Table 4B. Associations between *F2RL3* DNA methylation extent (all CpGs) and mortality in individuals experiencing a myocardial infarction in the Copenhagen City Heart Study (N=648) 51

Supplementary Table 5. Mediation analysis in ever smokers from smoking to myocardial infarction (N=2,461) and death after a myocardial infarction (N=561) 52

Supplementary Table 6. Characteristics of ALSPAC participants selected for invite (n=200) 53

Supplementary Table 7. Comparison of recruited sample of ALSPAC participants (n=41) and all those selected for invite (n=200) 55

Supplementary Table 8. Correlation across CpG sites in *F2RL3* of contemporary DNA methylation assessed by targeted pyrosequencing 56

Supplementary Table 9. By-group comparison of contemporary DNA methylation and platelet reactivity measures as assessed by flow cytometry 57

Supplementary Table 10A. By-group comparison of potential confounders of the relationship between DNA methylation and platelet reactivity in the recall study conducted in ALSPAC (N=41) 58

Supplementary Table 10B. Linear regression to explore effect of covariates associated with methylation group on platelet reactivity measures (N=41) 59

Supplementary Table 11. Linear regression to explore association of rs773902 with red blood cell count (N=41)..60

Supplemental Table 12. GTEx portal results for *F2RL3* (last accessed: 04/07/2018)[46] 61

Supplemental Table 13. eQTLGen results for rs773902/*F2RL3* (last accessed: 22/04/2021)[47] 62

Supplementary Methods

(i) Smoking, DNA hypomethylation and myocardial infarction

Sample

We studied individuals from the third wave of data collection in the Copenhagen City Heart Study, which took place between 1991 and 1994[59] with this timepoint serving as our baseline. All individuals with DNA (N=9,252) and who had experienced a myocardial infarction (definition below) before April 2013 (N=1,125) were selected to be assayed for DNA methylation, along with 3,167 age, sex and smoking status matched controls (ratio of approximately 1:3). Of these, 3,272 individuals were successfully assayed for DNA methylation at at least one of the four F2RL3 CpG sites (**Fig. 1B**) and 3,205 had DNA methylation measured at all four positions (see below for details of missing data). The CpG_3 at position 16,889,774-5 corresponds to the CpG labelled cg03636183 on the Illumina Infinium BeadChip (27k and 450k).

Myocardial infarction: Participants in the Copenhagen City Heart Study were followed up prospectively from the date they were seen at the 1991-1994 data collection (our baseline). Myocardial infarction diagnoses were ascertained from the national Danish Patient Register, from the national Danish Register of Causes of Death, and from medical records of general practitioners and hospitals. Myocardial infarction was defined according to WHO International Classification of Diseases 8th edition (ICD-8) code 410 until 1993, or 10th edition (ICD-10) codes I21-I22 from 1994 onwards[60]. Case status for sample selection was based on events recorded up to April 2013. Case status for statistical analyses was based on events recorded up to 10th November 2014.

Mortality after a myocardial infarction: Information on vital status, date of death or emigration (n=2) was obtained from the national Danish Civil Registration System from baseline until 14th November 2014.

Smoking: Individuals were classified as never, former or current smokers according to their self-reported smoking status at baseline. Current and former smokers were asked about age of smoking initiation, age of smoking cessation and consumption of cigarettes, cheroots, cigars and pipe tobacco. From this information, pack years of smoking were calculated; one pack year corresponds to smoking 20 cigarettes or equivalent per day for one year. In statistical models, pack years is expressed as a doubling of pack years. We

also improve the adjustment for smoking exposure in these analyses by using a second smoking-related DNA methylation site, the aryl hydrocarbon receptor repressor (*AHRR*), as a more refined measure of long-term exposure to cigarette smoke.

Demographics & covariates: Ancestry and statin usage were self-reported. The model covariates of age, systolic blood pressure (mmHg), total cholesterol (mmol/L) and self-reported passive smoking (yes/no) were recorded at baseline during study visits (i.e., at the same time the samples were collected). Details of the measurement of systolic blood pressure and total cholesterol have been previously published[61]. Case status for diabetes was based on a clinical diagnosis as recorded in the national Danish Patient Register with events recorded up to 10th November 2014.

DNA collection: DNA was isolated from frozen whole blood samples using the Qiagen Blood Kit resulting in an eluate with 5-30ng/µl. Twenty µl of this was treated with bisulfite using the EZ-96 DNA methylation Gold kit (Zymo Research). Elution volume was 60µl.

F2RL3 DNA methylation

DNA was isolated using the Norgen DNA/RNA extraction kit, resulting in an eluate with 5-50ng/µL. DNA was concentrated by ethanol precipitation and resuspension into a smaller volume where necessary. One µg of DNA was treated with bisulfite using EZ DNA methylation kit (Zymo Research, Irvine, CA) and eluted in 15µl. PCR and pyrosequencing were performed using 96-plate format with DNA from experimental samples and EpiTect control DNA, 100% and 0% methylated (Qiagen, Manchester, UK).

Four µl of the bisulfite treated DNA was added to a 50µl PCR reaction containing 1x Hot star Taq mastermix (Qiagen, Manchester, UK), 0.2µmol /L of each oligonucleotide (one biotin-labeled) and dH2O. PCR was performed using a Senquest PCR machine (Genflow, UK). The thermal cycle was programmed for 95˚C for 15 minutes, followed by 50 cycles of 95˚C for 15 seconds, 52˚C for 30 seconds, and 72˚C for 15 seconds. There was a final extension step of 72˚C for 5 minutes before amplicons were cooled to 4˚C. PCR products were confirmed by electrophoresis at 80V for 45 min in a 1% (W/V) agarose gel, in 1 x TBE buffer with 10µl Safeview nucleic acid stain (NBS (Cambridgeshire, UK) per 100ml and examined in UV light.

Pyrosequencing assays and primers to cover an extended genomic region around the CpG in *F2RL3* captured on the Illumina Infinium Human Methylation450 BeadChip (450K) array (cg03636183) were designed using the PyroMark Assay Design Software 2.0 (Qiagen Manchester, UK). Pyrosequencing was carried out using a PyroMark 96 ID pyrosequencer (Qiagen Manchester, UK) according to the manufacturer's recommendations. Forty µl of amplicons were used for downstream single strand preparation and hybridisation of 0.2µmol/L sequencing primer using a Qiagen vacuum prep tool and workstation according to manufacturer's instructions.

Oligo was purchased from IDT (Leuven, Belgium) and details were as follows:

Name F2LR3	Sequence 5'->3'
Forward oligo	GGGTTGGGTGTTTATTAGGT
Reverse oligo	/5BiosG/ACCAACAACAACACTAAACCATACATATA
Sequencing oligo	GTTTTGGTGGTGGGGTT

CpG captures: 4; Genomic location: chr19:16,889,739-16,889,790; amplicon length: 288; CpG sites: 742, 757, 775, 786

A total of 48 96-well plates were processed, each containing 90 study samples (plus control samples). Following quality control checks carried out by laboratory technicians, results from 11 of these plates (n=990 study samples) were deemed unreliable and therefore excluded from any further analysis. A further 30 samples did not return reliable methylation measures for any of the four CpG sites. Finally, 3,272 individuals had methylation data returned from the laboratory for at least one of the four CpG sites. In a final QC step implemented prior to statistical analysis, methylation values further than 5 standard deviations from the mean (taking each CpG site in turn) were set to missing. This resulted in seven samples having data set to missing for CpG_2. Following this final QC step, 3,205 individuals had DNA methylation values recorded at all four positions.

AHRR DNA methylation

Full details of the methods for measurement of *AHRR* DNA methylation, including assay validation, have been published previously[62]. DNA methylation of cg05575921 was assessed using a Taqman assay developed in house. The bisulfite treated DNA was amplified using forward and reverse PCR primers, which were designed to bind to DNA around the cg05575921 site on sequences without genetic or possible CpG DNA methylation variation. The probes detected either the unmethylated – and therefore converted T residue - or the methylated - and therefore conserved C residue.

Name	Sequence 5'->3'
Forward primer	GGGATTGTTTATTTTTGAGAGGGTAGTTT
Reverse primer	CTACCAAAACCACTCCCAAAACC
Probe detecting unmethylated cg05575921	VIC-AACCCAACCAAATACA
Probe detecting methylated cg05575921	FAM-AACCCAACCGAATACA

The thermal cycling profile was a conventional Taqman profile: 10 minutes at 95°C followed by 50 cycles of 15 seconds at 94°C, 60 seconds at 60°C, followed by cooling at 4°C.

After the end of the PCR reaction, the plates were read in a ViiA 7 Real-Time PCR System (Life Technologies). Samples were failed if the DNA methylation percentages from the duplicates were more than 30% from each other. Failed samples were measured again. Therefore, valid measurements of DNA methylation were available for more than 99.8% of available DNA samples.

Each 384-well plate contained two identical internal control samples. Plates failed if the DNA methylation percentages from these were more than 30% discrepant. Imprecision was measured across plates with the use of the result of internal control samples. Mean,

standard deviation, and coefficient of variation were calculated. Coefficients of variation varied from 5.0 to 6.7% for different stocks of the internal control.

Collider bias analysis

We assessed the potential for collider bias (induced by conditioning on myocardial infarction case status) to affect our analysis of the association of *F2RL3* DNA methylation with mortality[63]. To do this, we investigated whether there was evidence for correlations between *F2RL3* DNA methylation and other risk factors for myocardial infarction in our case only sample, that were not present in the case control sample. We explored systolic blood pressure and total cholesterol (assessed at baseline) as measured risk factors in this analysis. Firstly, we calculated the association of each of the traits (converted into z-scores) with myocardial infarction using Cox regression with age and sex fitted as covariates. Secondly, we used linear regression to assess the association of these traits with *F2RL3* DNA methylation in the full myocardial infarction case control sample and in the sample consisting of incident myocardial infarction cases only, again with age and sex fitted as covariates.

Extended statistics

Associations between *F2RL3* DNA methylation (per standard deviation decrease) and incident myocardial infarction were assessed in a series of three Cox regression models in which the time variable was set to be the date of the myocardial infarction event or the census date (in controls) and participants entered the study at baseline. Each model was fitted with an increasing number of covariates, as follows: Model 1: age (accounted for by setting date of birth as the origin) and sex; Model 2: model 1 plus *AHRR* DNA methylation; Model 3: Model 2 plus diabetes status, systolic blood pressure, total cholesterol and passive smoking. The same set of models were used to estimate associations between *F2RL3* DNA methylation and death following incident myocardial infarction. Data were restricted to incident myocardial infarction cases and Cox regression models set up with years since the event as the time variable and age at the event as a covariate.

The extent to which DNA methylation at *F2RL3* mediates the association between smoking (as per doubling of pack years in smokers) and risk of myocardial infarction or death after myocardial infarction was estimated by the product of coefficients method[23] based on the fully-adjusted Cox regression model described above (Model 3) with the addition of smoking status (former/current) as a covariate and without adjustment for percentage DNA methylation at *AHRR*. Proportion mediated was estimated as:

$$\frac{coefficient\ a \times coefficient\ b}{(coefficient\ a \times coefficient\ b) + coefficient\ c'},$$

where coefficient ‘a’ represents the relation of exposure (smoking) to mediator (methylation), coefficient ‘b’ represents the relation of mediator (methylation) to outcome (myocardial infarction or death after myocardial infarction) adjusted for exposure (smoking) and coefficient ‘c′’ represents the relation of exposure (smoking) to outcome (myocardial infarction or death after myocardial infarction) adjusted for mediator (methylation). This analysis was repeated with methylation at *AHRR* as the mediator.

(ii) Association between DNA hypomethylation and platelet function

Study Design

A recall by phenotype design was implemented in the Avon Longitudinal Study of Parents and Children (ALSPAC). ALSPAC is a trans-generational prospective birth cohort that began with the recruitment of 14,541 pregnant women resident in Avon, UK with expected dates of delivery 1st April 1991 to 31st December 1992. Since then, the health and development of mothers and their children has been followed across the life-course[26]. Further details of the cohort are available below in “**Supplementary Methods: Cohort information**” and the study website contains details of all the data that is available through a fully searchable data dictionary (http://www.bristol.ac.uk/alspac/researchers/access/). Participants were recruited based on DNA methylation at *F2RL3* as measured by Illumina Infinium HumanDNA methylation 450 BeadChip (450 K) array[25].

Ethical considerations and informed consent

Details of the ethics approvals relevant to the ALSPAC cohort in general can be found on the study website (http://www.bristol.ac.uk/alspac/researchers/research-ethics/). Ethical approval for this study was obtained from the ALSPAC Ethics and Law Committee and the NHS South West - Frenchay Research Ethics Committee (REC reference 14/SW/1099). All participants received a participant information sheet prior to being recruited to the study and were given the opportunity to ask questions both at the telephone screening and at their appointment. All participants were asked to complete a written consent form. Participants were appropriately reimbursed for their time and effort (as judged against other contemporary study initiatives) and all travel expenses reimbursed.

Participant recruitment

Between May 2015 and January 2016, we recalled young people (aged 22-24) from the ALSPAC cohort who had had DNA methylation assessed as part of the ARIES project (total N=1,007)[25]. Genome-wide DNA methylation profiling was conducted in these individuals from tissue taken at birth (cord blood), childhood (average age 7) and adolescence (age 15 or 17 years) (peripheral blood) using the Illumina Infinium HumanDNA methylation 450K BeadChip assay[25] (see "**Supplementary Methods: Cohort information**" for further details). In this study, data from the childhood and adolescence datasets (release v2[44]) were used post-normalisation by a pipeline described by Touleimat and Tost[64]. In addition, we adjusted DNA methylation values for cell composition (using estimated fractions of CD8 T cells, CD4 T cells, NK cells, B cells and monocytes), sex, clinic attended in the case of the adolescent measures (whether age 15 or age 17) and the technical artefacts of chip row and plate; a mixed model was fitted in which all factors were fitted as fixed effects except plate which was treated as a random effect.

To be eligible for invitation to the study, individuals had to have DNA methylation data available at both time points (childhood and adolescence) and have genome-wide genotype data (details of the SNP genotyping, imputation, processing and quality control procedures carried out in ALSPAC are in "**Supplementary Methods: Cohort information**"). As we were interested in *F2RL3* DNA methylation independent of smoking, we excluded individuals who reported; (i) smoking daily or weekly when they were age 13.5; or (ii) having

smoked more than 100 cigarettes in their lifetime at age 15; or (iii) being daily smokers at age 15. The 731 individuals that remained eligible for invite were ranked firstly according to DNA methylation at age seven and then according to DNA methylation at age 17; the average of these two rankings was then used to prioritise individuals for invitation to the study, with those with the highest average ranking and those with the lowest average ranking selected for invite. Initially, the top 50 and bottom 50 were invited to participate; this was extended to the top and bottom 100 during the study to meet target recruitment numbers. Some restrictions were imposed by the cohort (related to participant's commitment to other studies running concurrently with ours) such that the final number of invitations issued was n=147. Researchers and fieldworkers remained blind to participant status (high versus low DNA methylation group) throughout the recruitment, data collection and laboratory analysis phases of the study.

Individuals responding positively to the invitation were screened in a telephone interview and invited to attend clinic if they: 1) had never been a regular smoker; 2) did not have any haemostatic or cardiovascular disorders; 3) were not anaemic; 4) were not on insulin treatment; 5) had not had a major illness in the past; 6) did not regularly take anticoagulants, antiplatelet drugs or NSAIDS; 7) were not dependent on any substance apart from caffeine. Individuals eligible to take part were asked not to take ibuprofen or aspirin for seven days prior to their scheduled clinic visit; clinic visits were rescheduled in cases where such drugs had been taken in the seven days preceding their clinic visit. At the clinic, individuals were screened again for the exclusion criteria. A total of 49 individuals were recruited to the study and had blood samples taken. Eight participants were excluded due to exclusion criteria or technical issues during laboratory analysis.

Sample Collection

Participants provided a non-fasting blood sample (4.5mL) collected according to standard procedures. This was stored at room temperature in a vacutainer containing 1:9 v/v 4% trisodium citrate (BD Biosciences, Oxford, UK) and transported to the laboratory for analysis within two hours of being taken (range 21 – 79 minutes).

Sample analysis

P*reparation of platelet-rich plasma (PRP):* Haematologic parameters were tested using a Horiba Pentra ES60 Plus haematology analyser (Horiba UK Ltd, Northampton, UK), prior to centrifugation at 180 x g for 17 minutes. The PRP layer was removed and a platelet count was performed on a Z1 Coulter Particle Counter (Beckman Coulter, High Wycombe, UK). PRP was diluted in a modified HEPES-Tyrode's buffer (145 mM NaCl, 2.9 mM KCl, 10 mM HEPES, 1 mM MgCl2, 5 mM glucose, pH 7.3) to 3x107 platelets/mL. The buffy layer containing white blood cells was removed from the centrifuged whole blood and snap frozen in liquid nitrogen and stored at -80°C for subsequent DNA extraction and pyrosequencing (see '***F2RL3*** **DNA methylation**' below).

Flow Cytometry: For assessing $\alpha_{IIb}\beta_3$ integrin activation and P-selectin exposure, diluted PRP at a platelet count of 3x10^7 plts/mL was stimulated for 10 minutes at room temperature with either the PAR1-specific activating peptide, SFLLRN (0.25, 0.5, 1.0, 2.0, 3.0, 5.0 and 10.0 μM), or the PAR4-specific activating peptide, AYPGKF (20, 30, 50, 75, 100, 200 and 400 μM), in 50 μl reactions in the presence of 5 μl FITC conjugated mouse anti-human PAC-1 and 2.5 μl PE-conjugated mouse anti-human CD62P per reaction (Catalog no.'s 340507 and 555524, respectively, BD Biosciences, Oxford, UK). Unstimulated basal samples in the presence or absence of antibodies were used as controls. Unstimulated basal samples in the presence of 5 mM EDTA were included to give a measure of basal integrin activation. Samples were then fixed with an equal volume of 2% paraformaldehyde prior to analysis on a FACSCanto flow cytometer (BD Biosciences, Oxford, UK).

For assessing platelet surface receptor expression, diluted PRP at a platelet count of 3x10^7 plts/mL was incubated in 50 μl reactions in the presence of 1/10 dilution of PE-conjugated mouse anti-human CD41 (Catalog no. 12-0419-42, Thermo Fisher (Invitrogen), Paisley, UK), CD61 or IgG isotype control (Catalog no.'s 555754 and 555749, respectively, both from BD Biosciences, Oxford, UK) for 10 minutes at room temperature. Samples were then fixed with an equal volume of 2% paraformaldehyde prior to analysis on a FACSCanto flow cytometer (BD Biosciences, Oxford, UK).

F2RL3 DNA methylation

To provide a measure of contemporary DNA methylation at the locus, previously separated (and frozen) buffy layer was thawed, volume made up to 12 ml with lysis buffer (320 mM Sucrose, 1% Triton X-100, 5 mM MgCl2, 1 mM Tris HCl pH 7.5), mixed and centrifuged at 3000 x g for 15 min at room temperature. Supernatants were discarded and pellets placed on ice. Pellets were resuspended in 5 ml of lysis buffer and centrifuged at 3000 x g for 15 min, supernatants removed, and pellets returned to ice. Pellets were incubated in a water bath at 55°C for two hours with 5.22M GuHCl, 408 mM NH4Ac, 1.09% Na Sarcosyl and 0.217 mg/ml Proteinase K and vortexed every hour before transfer to 37°C for overnight incubation. Pellets were then left to reach room temperature before addition of 2ml chloroform, samples were then centrifuged at 3000 x g for 15 min. The upper aqueous layer was collected, added to 10 ml 100% ethanol without mixing and left overnight at -20°C. Tubes were gently inverted 50—60 times before centrifugation at 1900 x g for 15 min. Resultant pellets were washed twice by rinsing with 2 ml 70% ethanol followed by vortexing and centrifugation at 1900 x g for 5 min. DNA pellets were air dried for no longer than 10 mins before resuspension in 2mM Tris. 1µg of DNA was bisulfite converted using EZ DNA methylation Kit (Zymo Research, Irvine, CA) according to manufacturer's instructions. PCR and Pyrosequencing were performed as described in detail above (in section (i)) using 96-plate format with samples from participants and EpiTect control DNA (100% and 0% methylated, Qiagen, Manchester, UK). Samples were run in duplicates and results omitted if the DNA methylation percentages from duplicates differed by more than 5%.

Measured confounders

Pre-existing data were extracted for several traits relevant to cardiovascular health that may have acted as confounders in the relationship between *F2RL3* DNA methylation and measured platelet traits. Participant age at the first clinic appointment for the current study was estimated from their age at previously attended clinics. Maternal education level was taken from self-reported questionnaire data collected during pregnancy. Mothers reported their highest educational qualification as CSE/none, vocational, O level, A level, Degree or 'not known'. It was felt, however, that a lot of mothers with no educational qualifications merely left this whole question blank. The variable was therefore recoded such that all women who left everything blank were allocated to the 'CSE/none' category, leaving only those who ticked 'not known' as missing. For analyses presented herein, we recoded this variable to make a binary phenotype such that women from the categories CSE/none,

vocational and O level were coded 0, and those from the categories A level and Degree were coded 1.

Several variables recorded during the age 17-year clinic were included in analyses; the mean age of participants attending this clinic was 17.8 years. Body mass index (BMI, kg/m^2) was calculated as weight (kg)/height$(cm)^2$. Height was measured using a Harpenden stadiometer to the last complete mm. Participants were positioned with their feet flat and heels together so that their heels, calves, buttocks and shoulders came into contact with the vertical backboard. The headboard was lowered until it touched the participant's head and a 1kg weight was placed on the headboard to ensure head contact and to minimise hair thickness. Weight was measured using the Tanita Body Fat Analyser (Model TBF 401A). Participants were asked to undress to their underclothes and weight was measured to the nearest 50g. Resting blood pressure was measured using a DINAMAP 9301 machine. Blood pressure (mmHg) was taken twice on each arm and the average of the two measures calculated.

EDTA plasma derived from fasting blood samples collected during the age 17-year clinic and subsequently stored at −80 °C were analysed by a range of biological assays. A high-sensitivity CRP (hs-CRP) immunoturbidimetric assay was carried out by the staff of the Routine Lipids Section of the Biochemistry Department of Glasgow Royal Infirmary using a Hitachi Modular P Analyser and Roche Diagnostics Gmbh (D-68298 Mannhein) kit (Cat. no 1972855). Plasma lipids, including total cholesterol, were measured by enzymatic colorimetic assay with modification of the standard Lipid Research Clinics Protocol Assays were performed by the staff of the Routine Lipids Section of the Biochemistry Department of Glasgow Royal Infirmary using a Hitachi Modular P Analyser and Roche Diagnostics Gmbh (D-68298 Mannhein) kit (Cat. no 1491458). Cotinine was measured using the Cozart Cotinine Enzyme Immunoassay (Concateno, (now Alere Toxicology) UK, Abingdon) serum kit (M155B1). All samples, calibrators, and controls were brought to room temperature before use and were run in duplicate. Where required, samples were diluted using cotinine-free serum (fetal calf serum). Absorbance was measured spectrophotometrically at a wavelength of 450nm. The lowest calibrator used was 0.5ng/ml serum, and values below this were treated as undetectable/null (0ng/ml serum).

Extended statistics

Confounder checks: We tested for by-group differences in potential confounders of the relationship between methylation and platelet reactivity using a two-sample, two-sided t-test (assuming equal variances) unless the trait distribution did not approximate a normal distribution (based on a Shapiro-Wilk W-statistics <0.90) in which case, a two-sample Wilcoxon rank-sum (Mann-Whitney) test was conducted. By-group comparisons of categorical traits were based on a Pearson's chi-squared test. Where there was evidence for a between-group difference in a confounding factor, a linear model was fitted to check for an effect of that confounder on platelet traits found to be associated with methylation group (with platelet traits natural log transformed prior to analysis).

Exploratory analysis relating to rs773902: We used linear regression to estimate the relationship between rs773902 (A/G) genotype and DNA methylation at CpG_3 in ARIES. Finally, we used linear regression followed by Type II ANOVA (using the R 'car' package[65]) to explore the relationship between rs773902 (A/G) genotype, methylation group and platelet traits (after removal of outliers as described above and subsequent natural log transformation). Analyses were conducted in STATA v16.1[24] unless stated otherwise.

(iii) *In vitro* association between smoking and *F2RL3* DNA methylation and expression

Two cell types pertinent to CVD were used to evaluate the effect of cigarette smoke on *F2RL3*. Following exposure to cigarette smoke extract (CSE), *F2RL3* DNA methylation, mRNA expression and PAR4 protein expression were measured. In the main text, we present the data for the human megakaryocytic cell lineage (the acute megakaryocytic leukaemia cell line, CMK)[31]. Here we also present data for human coronary artery endothelial cells (HCAEC). The model was also applied to HCAEC because of their involvement in the development of CVD[34] and because this cell line was used in the previous development of then model protocol[29,30].

Cell culture

Acute megakaryocytic leukemia cells

Acute megakaryocytic leukemia (CMK) cells were kindly donated by Professor Ingeborg Hers, University of Bristol (of unknown passage). Cells were cultured in Iscove's Modified Dulbecco's Medium (IMDM) plus GlutaMAX, supplemented with 10% fetal bovine serum (FBS) and penicillin/streptomycin (Thermofisher, Catalog no.: 31980030, 10500064, 15140122). Cells were plated 2 hours before treatment with CSE, 4 doses 24 hours apart, analysing cell response 24 hours after final treatment (total 96 hours). CSE dosing very approximately equates to an average person smoking 25 cigarettes every 24 hours.

Human coronary artery endothelial cells

Human coronary artery endothelial cells (HCAEC) were purchased from PromoCell (Catalog no. C-12221) and cultured in Endothelial media MV2 (PromoCell, Catalog no. C-22121). Cells were plated 24 hours before treatment with CSE[29], 3 doses 16 hours apart, analysing cell response 16 hours after final treatment (total 48 hours)[30]. This was performed in triplicate using HCAEC from three different donors. The CSE is administered to cells as a bolus every 16 hours, because of the experimental complexity of administering smaller doses at shorter time intervals. This very approximately equates to an average person smoking 25 cigarettes[29].

F2RL3 DNA methylation

PCR and pyrosequencing were performed as described in detail above (in section (i)) using EpiTect control DNA, 100% and 0% methylated (Qiagen, Manchester, UK). Samples were excluded from downstream analyses if the DNA methylation percentages from duplicates failed or differences were more than 5%. It was possible for the methylation values of untreated samples to be lower than those treated, however runs where untreated samples were lower than the 0% negative controls were also failed and excluded.

For the CMK experiment, we performed 3 independent experiments comprised of pairs of untreated and CSE treated cells (1: n=2, 2: n=3, 3: n=4, total n=9). Data quality was assessed using the criteria described above. We included both sets of samples from experiment 1, were forced to exclude all 3 sets of samples from experiment 2 due to the controls exhibiting lower methylation recordings than the 0% negative controls and 2 of the 4 sets of samples from experiment 3 as the duplicate runs failed. This generated n=4

sets of samples which had data of sufficient quality to be included in our main analyses. These conditions were designed to only allow data of sufficient quality into analyses, however all data have been made publicly available at data.bris (see 'Data Access Statement' in main manuscript for details).

F2RL3 mRNA

Changes in CMK *F2RL3* mRNA expression were analysed by qPCR performed using the ThermoFisher Scientific TaqMan PCR mastermix on cDNA prepared using Superscript IV VILO master mix (ThermoFisher Scientific). qPCR for F2RL3 was performed using TaqMan Gene Expression Assay Hs01006385_g1 (FAM). Eukaryotic 18S rRNA endogenous control (Hs99999901_s1 (VIC)) was used in all samples. Ribosomal protein lateral stalk subunit P0 (RPLP0; Hs99999902_m1 (FAM)) was used as an additional control and was unchanged.

Changes in HCAEC *F2RL3* mRNA expression were analysed by Quantitative PCR (qPCR). qPCR was performed using the Roche SYBR green PCR mastermix on cDNA prepared using the QuantiTect Reverse Transcription Kit (Qiagen). qPCR for *F2RL3* was performed using primers 5' GGCAACCTCTATGGTGCCTA and 5' TTCGACCCAGTACAGCCTTC with denature – 94°C 15 seconds, anneal at 62°C for 20 seconds, extend at 72°C for 30 seconds.

Tandem mass tagging (TMT) protein quantification (CMK cells only)

Sample Preparation: CMKs cells cultured as per above were lysed at 1x107 cells/mL in 20mM HEPES, 150mM NaCl (pH7.4), 0.5% Triton X-100 and a Complete Mini protease inhibitor tablet (Roche Life Sciences, UK) at 4°C for 2 hours. Samples were clarified by centrifugation at 12000 x g for 30 minutes, before being snap frozen in liquid nitrogen and stored at -80 °C. Sample protein concentration was determined by Pierce BCA (bicinchoninic acid) protein assay (ThermoFisher Scientific, Altrincham, UK).

TMT Labelling and High pH reversed-phase chromatography: Aliquots of 100μg of each sample were digested with trypsin (2.5μg trypsin per 100μg protein; 37°C, overnight), labelled with Tandem Mass Tag (TMT) ten plex reagents according to the manufacturer's protocol (Thermo Fisher Scientific, Loughborough, LE11 5RG, UK) and the labelled

samples pooled. A 100ug aliquot of the pooled sample was evaporated to dryness, resuspended in 5% formic acid and then desalted using a SepPak cartridge according to the manufacturer's instructions (Waters, Milford, Massachusetts, USA). Eluate from the SepPak cartridge was again evaporated to dryness and resuspended in buffer A (20 mM ammonium hydroxide, pH 10) prior to fractionation by high pH reversed-phase chromatography using an Ultimate 3000 liquid chromatography system (Thermo Scientific). In brief, the sample was loaded onto an XBridge BEH C18 Column (130Å, 3.5 μm, 2.1 mm X 150 mm, Waters, UK) in buffer A and peptides eluted with an increasing gradient of buffer B (20 mM Ammonium Hydroxide in acetonitrile, pH 10) from 0-95% over 60 minutes. The resulting fractions were evaporated to dryness and resuspended in 1% formic acid prior to analysis by nano-LC MSMS using an Orbitrap Fusion Tribrid mass spectrometer (Thermo Scientific).

Nano-LC Mass Spectrometry: High pH RP fractions were further fractionated using an Ultimate 3000 nano-LC system in line with an Orbitrap Fusion Tribrid mass spectrometer (Thermo Scientific). In brief, peptides in 1% (vol/vol) formic acid were injected onto an Acclaim PepMap C18 nano-trap column (Thermo Scientific). After washing with 0.5% (vol/vol) acetonitrile 0.1% (vol/vol) formic acid peptides were resolved on a 250 mm × 75 μm Acclaim PepMap C18 reverse phase analytical column (Thermo Scientific) over a 150 min organic gradient, using 7 gradient segments (1-6% solvent B over 1min., 6-15% B over 58min., 15-32%B over 58min., 32-40%B over 5min., 40-90%B over 1min., held at 90%B for 6min and then reduced to 1%B over 1min.) with a flow rate of 300 nl min^{-1}. Solvent A was 0.1% formic acid and Solvent B was aqueous 80% acetonitrile in 0.1% formic acid. Peptides were ionized by nano-electrospray ionization at 2.0kV using a stainless steel emitter with an internal diameter of 30 μm (Thermo Scientific) and a capillary temperature of 275°C.

All spectra were acquired using an Orbitrap Fusion Tribrid mass spectrometer controlled by Xcalibur 3.0 software (Thermo Scientific) and operated in data-dependent acquisition mode using an SPS-MS3 workflow. FTMS1 spectra were collected at a resolution of 120 000, with an automatic gain control (AGC) target of 200 000 and a max injection time of 50ms. Precursors were filtered with an intensity threshold of 5000, according to charge state (to include charge states 2-7) and with monoisotopic peak determination set to peptide. Previously interrogated precursors were excluded using a dynamic window (60s +/-10ppm).

The MS2 precursors were isolated with a quadrupole isolation window of 1.2m/z. ITMS2 spectra were collected with an AGC target of 10 000, max injection time of 70ms and CID collision energy of 35%.

For FTMS3 analysis, the Orbitrap was operated at 50 000 resolution with an AGC target of 50 000 and a max injection time of 105ms. Precursors were fragmented by high energy collision dissociation (HCD) at a normalised collision energy of 60% to ensure maximal TMT reporter ion yield. Synchronous Precursor Selection (SPS) was enabled to include up to 5 MS2 fragment ions in the FTMS3 scan.

Data analysis: The raw data files were processed and quantified using Proteome Discoverer software v2.1 (Thermo Scientific) and searched against the UniProt Human database (downloaded October 2019; 150786 sequences) using the SEQUEST algorithm. Peptide precursor mass tolerance was set at 10ppm, and MS/MS tolerance was set at 0.6Da. Search criteria included oxidation of methionine (+15.995Da), acetylation of the protein N-terminus (+42.011Da) and Methionine loss plus acetylation of the protein N-terminus (-89.03Da) as variable modifications and carbamidomethylation of cysteine (+57.021Da) and the addition of the TMT mass tag (+229.163Da) to peptide N-termini and lysine as fixed modifications. Searches were performed with full tryptic digestion and a maximum of 2 missed cleavages were allowed. The reverse database search option was enabled, and all data was filtered to satisfy false discovery rate (FDR) of 5%.

Global DNA demethylation with 5-Azacytidine (HCAEC cells only)

In HCAEC not exposed to CSE, we also investigated the impact of global DNA demethylation on *F2RL3* mRNA expression by culture with 5-Azacytidine[66]. HCAEC were seeded into a 6-well plate at 1.4 x 10^5 cells per well. 2.5 hours after seeding 5-azacytidine (5-AZA at a final concentration of 2.5μM, Sigma, UK) or DMSO control (1/4,000 final) was added to the cells. Media was replaced on day 2, 3 and 4, with fresh 5-AZA or DMSO control. Cells were analysed for gene expression (as described above) on day 5. This was performed four times using HCAEC from four different donors.

Extended statistics

Statistical analysis (for HCAEC experiments): Analyses of methylation and mRNA expression data were conducted in Stata (version 16.1)[24]. Average *F2RL3* DNA methylation values at each of the four sites were compared in CSE-exposed cells (n=3) were compared to levels in unexposed controls (n=3) using two sample t-tests assuming equal variances. Ratios of *F2RL3* mRNA levels in CSE-exposed cells (n=6) compared to unexposed controls (n=4) were natural log transformed and an average taken. The average log ratio for CSE-exposed cells was compared to the value in unexposed controls (i.e., baseline) using a two-sample t-test assuming equal variances. The average log ratio was then exponentiated along with its 95% confidence intervals (CIs) to derive an estimate of mean fold change. For the 5-Aza experiment, ratios of DMSO control (n=4) and HCAEC cells treated with 5-AZA (n=4) were log transformed and averaged. These were compared to the baseline value in untreated controls, 1 (0 when log transformed), using one sample t-tests. The average log ratios were exponentiated along with their 95% CIs to estimate mean fold change.

TMT-derived protein abundance data: Analysis of TMT-derived protein abundance data across all proteins measured was conducted in Excel. First, abundance measures from the three independent experimental runs were log2 transformed. A comparison of mean abundances was conducted on the transformed abundances using a two-sample t-test assuming unequal variance. A log2 fold change was then calculated by subtracting the mean log2 abundance in control cell samples from the mean log2 abundance in the CSE-exposed cell samples.

(iv) Functional regulation of *F2RL3*

Reporter assay

To investigate the potential mechanism by which differential methylation could impact on *F2RL3* gene expression, a series of reporter constructs containing different fragments of *F2RL3* to drive expression of luciferase were used.

We used a pGL3 reporter vector to test for the presence of an enhancer within a fragment of *F2RL3* exon 2 containing CpG_1 to CpG_4. We used a vector in which luciferase expression is driven by a heterologous SV40 promoter to test for the presence of an

enhancer within a 184 bp fragment of *F2RL3* exon 2 containing CpG_1 to CpG_4. The pGL3 was a kind gift from Dr G Sala-Newby, University of Bristol. The 184 bp *F2RL3* exon 2 sequence (see below) was generated by PCR using the primers GATCTTAAGCTTACAGTGACACCCTGGAGC and AAGCTTAAGATCAGGTTCATCAGCAGCATG, which included HindIII restriction sites to enable cloning into the multiple cloning sites of the vectors.

Subsequently, the potential mechanisms of effects on *F2RL3* expression were explored by transfecting HEK-293 cells (a cell line derived from human embryonic kidney cells grown in tissue culture, grown in Eagle's Minimum Essential Medium with 10% FCS, kindly donated by Dr Graciela Newby, University of Bristol and of unknown passage) with reporter constructs containing different fragments of *F2RL3* to drive expression of luciferase. The pCpGL vector used was a generous gift from Prof M Rehli, University of Regensberg[67]. The *F2RL3* putative promoter (F2RL3pro) sequence corresponding to the 2 kB sequence immediately upstream of the *F2RL3* transcription initiation site (see sequence below) flanked by BamH1 and HindIII restriction sites was synthesised (Eurofins). Inspection of the *F2RL3* exon 2 region with MatInspector[68] to identify candidate DNA-binding protein interaction sites[69] revealed a recognition site for CCAAT/enhancer binding protein (CEBP) at 2 bp 3' of the CpG_1 DNA methylation site (main manuscript, Fig. 1B)[1]. The exon 2 ΔCCAAT fragment was generated from the intact exon 2 fragment by site directed deletion of the CCAAT sequence using the primers GGGCTGGCGCTGTGGGTG and CCGGCAGCCCCACCACCA (Phusion site directed mutagenesis kit, New England BioLabs).

F2RL3 2 kB promoter sequence:

CTGGCTTTTTTTTTTTTTTTTTTTTTTTTTTGAGTCGGAGTCTCGCTCCG

TTGCCCAGGCTGGAGTGCAGTGGTGTGATCTCAGCTCACTGTAACCCCTG

CGTCCTGGGTTCAAGAGATTTTCCTGCCTCAGCCTCCTGAGGAGCTAGGA

[1] Of note, CEBP is a transcriptional regulator in multiple tissues including haemopoietic cells and vascular endothelium[75] in which *F2RL3* is also expressed[76]. CEBP binding to an identical CCAAT recognition site at a different locus (*MLH1*), is known to be reduced by DNA methylation of a CpG residue in an identical relative position to the CCCAT recognition sequence to that observed with CpG_1[40].

CTACAGACGTGCGCCACCATGTCTGGCTAATTTTTGTATTTTTAGTAGAG

ACGGGTTTTCACCATGTTGGCCAGGACGGTCTCGATCTCTTGACCTCGTG

ATCCACCCACCTGGCCTCCCAAAGTGCTGGGATTACAGGCGTGAGCCATT

GCGCCCGGTCGTGCATCACTATTTTGATGCATCATCTTCAAACCACCCTG

CCCCAGCATCACTGGACTGCCGGTGTGCCCAGCCTCCCCTGCACAGCTTC

CCACTCTGCTCACAGAGAAGACGGTGGAGGGGGGAGCCCAGGCGTGGGTT

TTCCGTGTGCTGAGGGTGTCCTCAGCCACCTTTTTCCCCAAGTCATTGAC

GTGAAGCTGATTTTCTCATTGGTGGCCATGGAAACTGCCCAGAAATGGGA

GCAGGGCCTCTGAGACCCTGCCCCAGCTAGACTCTCTCTCCAGGCTTCAG

TGTCTCCTCCTGGAGCTGGGTGTTGACTCTGTCCAAGACTCTGCTCAGTC

ACTTCCTGGCTGATCCCCGAGTGCTCAAGTGTGTGATGAGATTAGGGCTC

CCCCAGGCAGGAAACGGCCACATTGGACCCCTCCCTCCCCCATCCCTAGT

TCATTTTATTTTCCCCAATCCTCATGGCTCCCAACTAGGCCCTCCTGCTC

CCAGACCCCCCCAAGCCCTGTCCCCCATCCCCTGTAGCCCAGCGCTGAGC

ACACTGGGGCCAGAACCACCCATACCAGACTCCCCCAGGGCACGACGGGT

TGAGGCCTCTCTGAGGCAGGGAGGGACAACACTGAGGGGCCTTGTGCAGA

AGAGGGAAGGGGTACCCCCCCGAGCTTCAGTTTCTTCATCTGCCCGATGA

GCCCCCATCCAATGGTACCCAGGAGTGGGGATGTGCTGGGGCTAGAGGAG

GGAGGCCCCCTGGAGGCTGGCTTGGGGACCCTTCAACCTGGCCACCTCAC

TCAGGGTCAGAGGTCAGAGCAGGCGAGCCACACACCCCGAAGTCCCCCCG

GTCCTCGGCAGGTGCTGTCCCCAGCATCACTGTCTCCCCTACAGATGCCC

GTGTGTCCCCTGGGCAAGAGTGAAGTGAGGCACTCATACAGGGGTGCCTT

GTGAGTATGGGGTCAGCCGAGGATGCTGAGGGGCTCTCGAGGTTCAGCCA

GAGTCCCTGACGCTGGGCTCGTGCTGCTCTGGGGGTCTGAAGGGACCCTC

GCTATTACCCTCTGGGAGGCGCCCATCCTTGGTTTTTTTTTTTTTTTTTG

CCGAGTCTCGCTCTGTTGCCCAGGCTGGAGTGCAGTGGCAAGATCTCGGC

TCACTGCAACCTCCACCTCCTGGGTTCAAGCAATTCTCCCACCTCAGCCT

CCTGAGTAGCTGGGACCACAGGCATCTGCCACCAAGCCCAGCTAATTTTT

GTATTTTTATGAGAGATGGGGTTTCTCCATGTTGGTCAGGCCGGTCTCAA

ACTCCCAACCTCAGGTGATTTGCCTGCCTTGGCCTCCCAAAATGTTGGGA

TTACAGCCGTGAGCCACCGTGCCTGGCCTAGCAGGGCTCTTCTTATCCTG

CTCACCAGAGATAGGAAGTCGGGGGGTGACTTCAGGAAAGGCCCAGAGCC

CGTGATGGGCGTGGGGATTGGGGTGAGGACAGGGCTGTCGTGAGAACAGT

GGCTGCAGATGTGAGCGGCTGGCAGGAAGTGGCCACTTGAACCGCAGATG

CTTCCTGGGCTGGTCAGGGACCGGGGGTGCTGGCTGCAGCTGGGACCCCC

CCCCCTGCATCTTGCTGGCCTGGCACCTGGGTCCCTGGGAGGCGCCACAC

TGGATATAGCCACGTGGGGCAGCCCGGTCTCCATAACCCACACTCCAGTC

Expression cassette analysis was performed in HEK-293 cells. $5x10^4$ cells were seeded into each well of a 24-well plate and transfected with 425ng test plasmid expressing firefly luciferase with 75ng of Renilla-luciferase control using 2µl Viafect (Promega, UK). Analysis of gene expression was performed after 48 hours using Dual-Luciferase Reporter Assay System (Promega, UK) and expressed as a ratio of firefly luciferase/Renilla luciferase.

We began with an expression model with a promoter-less pCpGL reporter vector and to set a baseline comparator, the *F2RL3* putative promotor sequence was inserted immediately upstream of the transcription start site adjacent to the luciferase cDNA in pCpGL

(pCpGL_*F2RL3*pro). Subsequently, the *F2RL3* exon 2 fragment only was inserted into the pCPGL vector (pCpGL_exon2) and then both the *F2RL3* promoter region and the exon 2 fragment (pCpGL_*F2RL3*pro_exon2). To investigate whether the CCAAT/enhancer binding protein (CEBP) recognition sequence in *F2RL3* exon 2 was functional and involved in DNA methylation-dependent regulation of *F2RL3,* we assessed luciferase activity again having deleted the CEBP recognition sequence (pCpGL_*F2RL3*pro_exon2 CCAAT deletion). A supplementary analysis was conducted to examine the impact of global DNA demethylation on DNA-protein interactions at the CEBP locus using chromatin immunoprecipitation (ChIP) (see below).

Statistics

Statistical analyses were performed in Stata version 16.1[24]. Ratios of luciferase expression in HEK-293 cells (n=3) transfected with a pGL3 vector containing *F2RL3* exon 2 with CpG_1 to CpG_4 compared to pGL3 vector alone were log transformed and averaged. The average value was then exponentiated along with its 95% CI. Ratios of luciferase activity in HEK-293 cells transfected with different exon2 fragments relative to the promotor only construct (n=6) were log transformed and averaged. These were tested against the baseline value, 1 (0 when log transformed), using one sample t-tests. Luciferase activity in the vector containing the *F2RL3* exon 2 fragment was tested against the same fragment following deletion of the CCAAT binding site using a two-sample two-sided t-test assuming equal variance. Average log ratios were exponentiated along with their 95% CIs.

Chromatin immunoprecipitation (ChIP) in human coronary artery endothelial cells (HCAEC)

Sample preparation: Following 5-Aza treatment of HCAEC as described above in section (iii) (under the subheading '**Global DNA demethylation with 5-Azacytidine (HCAEC cells only)**'), cells were washed with cold PBS and fixed with 1% formaldehyde at room temperature for 10 min, before being quenched using 1/10 volume of 2.5 M glycine for 5 mins at room temp. Cells were washed twice with 20 ml of ice-cold phosphate-buffered saline. Cell Lysis was performed in 0.75 ml of SDS ChIP lysis buffer [1% SDS, 10 mM EDTA, 50 mM Tris-HCl, pH 8.1, 1 mM PMSF, 1 ug/ml leupeptin, 1 ug/ml aprotinin]. Samples were aliquoted in 300µl portions and stored at -80°C. DNA was sheared using 2.5

cycles (10x 30sec on/30sec off) in a Diagenode-Bioruptor sonicator (high power setting at 4°C) and centrifuged at 15k rpm at 4°C for 10 minutes. Protein concentration was measured using the Pierce Micro BCA Protein Assay.

ChIP: 150µg of protein was used for ChIP, all samples were made up to the same volume using ChIP lysis buffer to ensure the SDS concentration were equivalent. Sample volume was increased with ChIP dilution buffer [1% Triton X-100, 2 mM EDTA, 150 mM NaCl, 20 mM, Tris-HCl, pH 8] with 1/100 Protease inhibitor (Sigma), to ensure that cell lysates were diluted at least 10 times. 2.5µg Rabbit anti-CEBP Beta antibody [E299] (ab32358, Abcam) or IgG control (Vector) were added and incubated overnight at 4°C on a rotary mixer.

Magnetic bead preparation: At same time as ChIP incubation, 25µl of Dynabeads® Protein A (30 mg/mL, #10002D, Life Technologies) were blocked at 4°C overnight with 75µl of blocking buffer [50 µg/mL Deoxyribonucleic acid from salmon sperm (#31149, Sigma), 0.5% BSA (w/v), in ChIP Dilution buffer]. Beads were then washed once with sterile PBS and twice with ChIP dilution buffer, using the magnet to pull the beads down in between each wash.

Binding of immunoprecipitate (IP) onto beads: The IP and non-immune control were added to the blocked and washed beads and incubated at 4°C for 3 hours on rota. The magnetic beads were then sequentially washed at 4°C for 5 minutes with 500µl of wash buffer 1 [0.1% SDS, 1% Triton X-100, 2 mM EDTA, 20 mM Tris-HCl, pH 8.1, 150 mM NaCl]; 500µl of wash buffer 2 [0.1% SDS, 1% Triton X-100, 2 mM EDTA, 20 mM Tris-HCl, pH 8.1, 500 mM NaCl]; 500µl of wash buffer 3 [0.25 M LiCl, 1% Nonidet P-40, 1% deoxycholate, 1 mM EDTA, 10 mM Tris-HCl, pH 8.1]; and twice with 500µl of TE [10mM Tris, 1mM EDTA, pH 8.0]. The TE was removed and the beads were incubated with two sequential 15 minute elution steps (2 x 75µl of elution buffer [1% SDS, 100mM NaHCO3]) at 65°C, vortexing briefly every 5 minutes. A 'total input' sample was also prepared containing 1/16th of original input in 150µl elution buffer.

Reverse cross link and sample extraction: 6µl of 5M NaCl (200mM final) was added to each tube (IP, non-immune and total input), mixed and incubated overnight at 65°C. Samples were vortexed vigorously (2 minutes for each tube) and 150µl of 10mM Tris pH 8 added to each tube. Samples were phenol-chloroform extracted, DNA precipitated and dissolved in 10mM Tris pH8. 1 µl of sample was used in each PCR reaction.

Quantitative PCR: Quantitative PCR was performed using Roche SYBRgreen 2x mastermix with an anneal temperature of 67°C using primers SW855F (5' – GACAGTGACACCCTGGAGCTCCC) and SW858R (5'- CCAGCACCCACAGCGCCAGC). The relative occupancy of the F2RL3 exon 2 CEBP recognition site was estimated as the ratio of the intensity of the specific IP band to that of the mock IP band in gel electrophoresis.

Statistical analysis: Ratios of the % occupancy of the *F2RL3* exon 2 CEBP recognition site with CEBP-β in 5-AZA treated cells compared to controls were calculated for each batch of cells (n=5). Ratios were natural log transformed and the mean taken. The mean following 5-Aza treatment (n=5) was compared to the control mean (n=5) by paired two-sided t-test. Mean fold change in treated cells was calculated by exponentiating the mean difference in log transformed values.

Cohort Information

The Avon Longitudinal Study of Parents and Children (ALSPAC)

ALSPAC: Description of study numbers

Pregnant women resident in Avon, UK with expected dates of delivery 1st April 1991 to 31st December 1992 were invited to take part in the study. The initial number of pregnancies enrolled is 14,541 (for these at least one questionnaire has been returned or a "Children in Focus" clinic had been attended by 19/07/99). Of these initial pregnancies, there was a total of 14,676 foetuses, resulting in 14,062 live births and 13,988 children who were alive at 1 year of age.

When the oldest children were approximately 7 years of age, an attempt was made to bolster the initial sample with eligible cases who had failed to join the study originally. As a result, when considering variables collected from the age of seven onwards (and potentially abstracted from obstetric notes) there are data available for more than the 14,541 pregnancies mentioned above. The number of new pregnancies not in the initial sample (known as Phase I enrolment) that are currently represented on the built files and reflecting enrolment status at the age of 24 is 913 (456, 262 and 195 recruited during Phases II, III and IV respectively), resulting in an additional 913 children being enrolled. The total sample

size for analyses using any data collected after the age of seven is therefore 15,454 pregnancies, resulting in 15,589 foetuses. Of these 14,901 were alive at 1 year of age.

A 10% sample of the ALSPAC cohort, known as the Children in Focus (CiF) group, attended clinics at the University of Bristol at various time intervals between 4 to 61 months of age. The CiF group were chosen at random from the last 6 months of ALSPAC births (1432 families attended at least one clinic). Excluded were those mothers who had moved out of the area or were lost to follow-up, and those partaking in another study of infant development in Avon.

ALSPAC: Genotyping description

ALSPAC children were genotyped using the Illumina HumanHap550 quad chip genotyping platforms by 23andme subcontracting the Wellcome Trust Sanger Institute, Cambridge, UK and the Laboratory Corporation of America, Burlington, NC, US. The resulting raw genome-wide data were subjected to standard quality control methods. Individuals were excluded on the basis of sex mismatches; minimal or excessive heterozygosity; disproportionate levels of individual missingness (>3%) and insufficient sample replication (IBD < 0.8). Population stratification was assessed by multidimensional scaling analysis and compared with Hapmap II (release 22) European descent (CEU), Han Chinese, Japanese and Yoruba reference populations; all individuals with non-European ancestry were removed. SNPs with a minor allele frequency of < 1%, a call rate of < 95% or evidence for violations of Hardy-Weinberg equilibrium (P < 5E-7) were removed. Cryptic relatedness was measured as proportion of identity by descent (IBD > 0.1). Related subjects that passed all other quality control thresholds were retained during subsequent phasing and imputation. 9,115 subjects and 500,527 SNPs passed these quality control filters.

ALSPAC mothers were genotyped using the Illumina human660W-quad array at Centre National de Génotypage (CNG) and genotypes were called with Illumina GenomeStudio. PLINK (v1.07)[70] was used to carry out quality control measures on an initial set of 10,015 subjects and 557,124 directly genotyped SNPs. SNPs were removed if they displayed more than 5% missingness or a Hardy-Weinberg equilibrium P-value of < 1E-6. Additionally, SNPs with a minor allele frequency of less than 1% were removed. Samples were excluded if they displayed more than 5% missingness, had indeterminate X chromosome

heterozygosity or extreme autosomal heterozygosity. Samples showing evidence of population stratification were identified by multidimensional scaling of genome-wide identity by state pairwise distances using the four HapMap populations as a reference, and then excluded. Cryptic relatedness was assessed using an IBD estimate of more than 0.125 which is expected to correspond to roughly 12.5% alleles shared IBD or a relatedness at the first cousin level. Related subjects that passed all other quality control thresholds were retained during subsequent phasing and imputation. 9,048 subjects and 526,688 SNPs passed these quality control filters.

ALSPAC: Imputation description

477,482 SNP genotypes in common between the sample of mothers and sample of children were combined. SNPs with genotype missingness above 1% due to poor quality were removed (11,396 SNPs removed). 321 subjects were removed due to potential ID mismatches. This resulted in a dataset of 17,842 subjects containing 6,305 duos and 465,740 SNPs (112 were removed during liftover and 234 were out of HWE after combination). Haplotypes were estimated using ShapeIT (v2.r644) which utilises relatedness during phasing. A phased version of the 1000 genomes reference panel (Phase 1, Version 3) was obtained from the Impute2 reference data repository (phased using ShapeIt v2.r644, haplotype release date Dec 2013). Imputation of the target data was performed using IMPUTE V2.2.2[71,72] against the reference panel (all polymorphic SNPs excluding singletons), using all 2,186 reference haplotypes (including non-Europeans). This gave 17,842 mothers and children eligible for study with available genotype data. Subsequent consent withdrawals have left 17,825 individuals for study.

ALSPAC: ARIES description

Samples were drawn from the Avon Longitudinal Study of Parents and Children[26,73]. Blood from 1018 mother–child pairs (children at three time points and their mothers at two time points) were selected for analysis as part of the Accessible Resource for Integrative Epigenomic Studies (ARIES, http://www.ariesepigenomics.org.uk/) (Relton 2015). Following DNA extraction, samples were bisulphite converted using the Zymo EZ DNA Methylation™

kit (Zymo, Irvine, CA, USA). Following conversion, genome-wide methylation was measured using the Illumina Infinium HumanMethylation450 (HM450) BeadChip. The arrays were scanned using an Illumina iScan, with initial quality review using GenomeStudio. ARIES consists of 5469 DNA methylation profiles obtained from 1022 mother-child pairs measured at five time points (three time points for children: birth, childhood and adolescence; and two for mothers: during pregnancy and at middle age). Full details of the pre-processing and normalization of ARIES v2 has been described previously[25,44].

ARIES was funded by the BBSRC (BBI025751/1 and BB/I025263/1). ARIES is maintained under the auspices of the MRC Integrative Epidemiology Unit at the University of Bristol (MC_UU_12013/2 and MC_UU_12013/8).

Supplementary Results

(i) Smoking, DNA hypomethylation and myocardial infarction

Collider bias analysis

We undertook an analysis of risk factors for myocardial infarction incidence to evaluate the possibility of the mortality analysis (where only cases are selected) being affected by collider bias. By comparing the association between myocardial infarction incidence risk factors in unselected (Full) and selected (myocardial infarction cases) samples, we can identify potential issues. Myocardial infarction incidence risk factors which are not associated with each other in a random population sample may become associated in a selected sample and act as a source of confounding in the association with an outcome such as mortality.

Supplementary Figure 2A shows the association of two risk factors with myocardial infarction incidence, indicating that there is potential for these factors to be influencing collider bias in the analysis of methylation and mortality in myocardial infarction cases. **Supplementary Figure 2B** shows that the associations between these risk factors and *F2RL3* methylation did not vary between the two groups (Full and myocardial infarction cases) and no associations were seen in the selected sample, suggesting there is unlikely to be collider bias of any appreciable magnitude affecting our results.

(ii) Association between DNA hypomethylation and platelet function

Results of exploratory analyses relating to rs773902

Fitting methylation group and rs773902 genotype together as independent variables improves the fit (based on adjusted R^2) when compared to fitting either variable on their own, suggesting independent contributions to both integrin activation EC_{50} and P-selectin exposure EC_{50} (**Supplementary Table 10B**). There appeared to be no effect of rs773902 genotype (with or without additional adjustment for methylation group) on any of the other platelet or haematology parameters measured in the study including PAR1-stimulated platelet measures (as assessed by linear regression, data not shown) except for red blood cell count. Red blood cell count was itself weakly associated with both integrin activation EC_{50} and P-selectin exposure EC_{50} (after PAR4-specific stimulation) (**Supplementary Table 11**).

(iii) *In vitro* association between smoking and *F2RL3* DNA hypomethylation and expression

Human coronary artery endothelial cell results

Exposure of cells to cigarette smoke extract (CSE) reduced *F2RL3* DNA methylation in HCAEC at CpG_1 and CpG_2 (**Supplementary Figure 5A**). This reduction in DNA methylation was accompanied by a 5.4 (95% CI: 3.9, 7.6, p<0.0001) fold increase in *F2RL3* mRNA levels relative to untreated controls (**Supplementary Figure 5B**).

The culture of HCAEC with 5-Azacytidine to induce global DNA hypomethylation resulted in a mean 14.8 (95% CI: 4.5, 48.3, p=0.005) fold increase in *F2RL3* expression over untreated controls while no change in expression was seen in DMSO-treated cells (fold change of 1.04, 95% CI: 0.67, 1.61, p=0.80).

(iv) Functional regulation of *F2RL3*

Results of reporter assay

Insertion of a fragment of *F2RL3* exon 2 containing CpG_1 to CpG_4 into a pGL3 reporter vector resulted in a 5.8-fold (95% CI: 3.0, 11.2, p=0.007) increase in luciferase activity compared to a pGL3 vector alone. Combining both the *F2RL3* promoter and the *F2RL3* exon 2 fragment in the pCpGL reporter vector (pCpGL_*F2RL3*pro_exon2) resulted in increased luciferase activity relative to the promotor only construct (**Supplementary Figure 7**), suggesting that the exon 2 region has enhancer activity that acts with the endogenous *F2RL3* promoter. Mutation of the CEBP recognition sequence in exon 2 (pCpGL_*F2RL3*pro_exon2 CCAAT deletion) attenuated luciferase reporter gene activity (**Supplementary Figure 7**), suggesting that whilst this regulatory element was not completely responsible for expression, presence or absence is important and that the CEBP recognition sequence is necessary for full enhancer activity.

Chromatin immunoprecipitation (ChIP) in human coronary artery endothelial cells (HCAEC) results

Culture of HCAEC with the DNA methyltransferase inhibitor, 5-Azacytidine resulted in a 4.9-fold (95% CI: 1.7, 14.0, p=0.01) increased occupancy of the *F2RL3* exon 2 CEBP

recognition site with CEBP-β (a prototypical isoform abundantly expressed in haemopoietic tissue and endothelium[36]), quantified by ChIP.

Supplementary Figures

Supplementary Figure 1. The Pearson correlation between the extent of DNA methylation at CpG_1 to CpG_4 (N=3,205)

Lower triangle shows correlations calculated in 548 never smokers; upper triangle shows correlations calculated in 1,589 current smokers (Copenhagen City Heart Study).

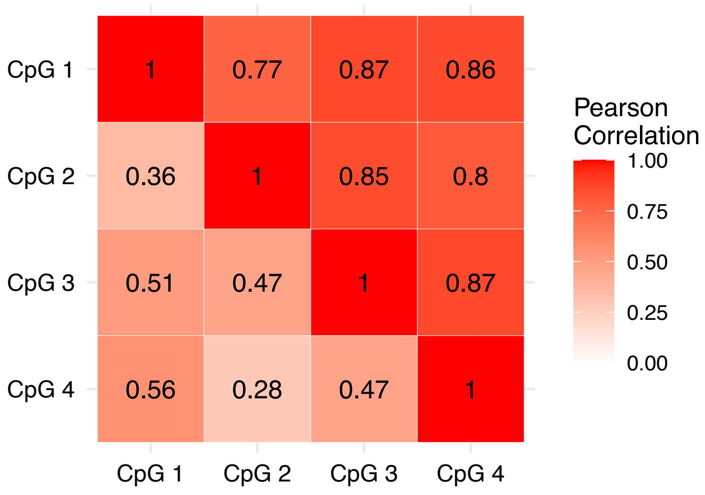

Supplementary Figure 2. Results from an assessment of collider bias and its potential impact on the case-only analysis

(A) Associations between systolic blood pressure (mmHg) and total cholesterol (mmol/L) and myocardial infarction in the Copenhagen City Heart Study (N=2,998)

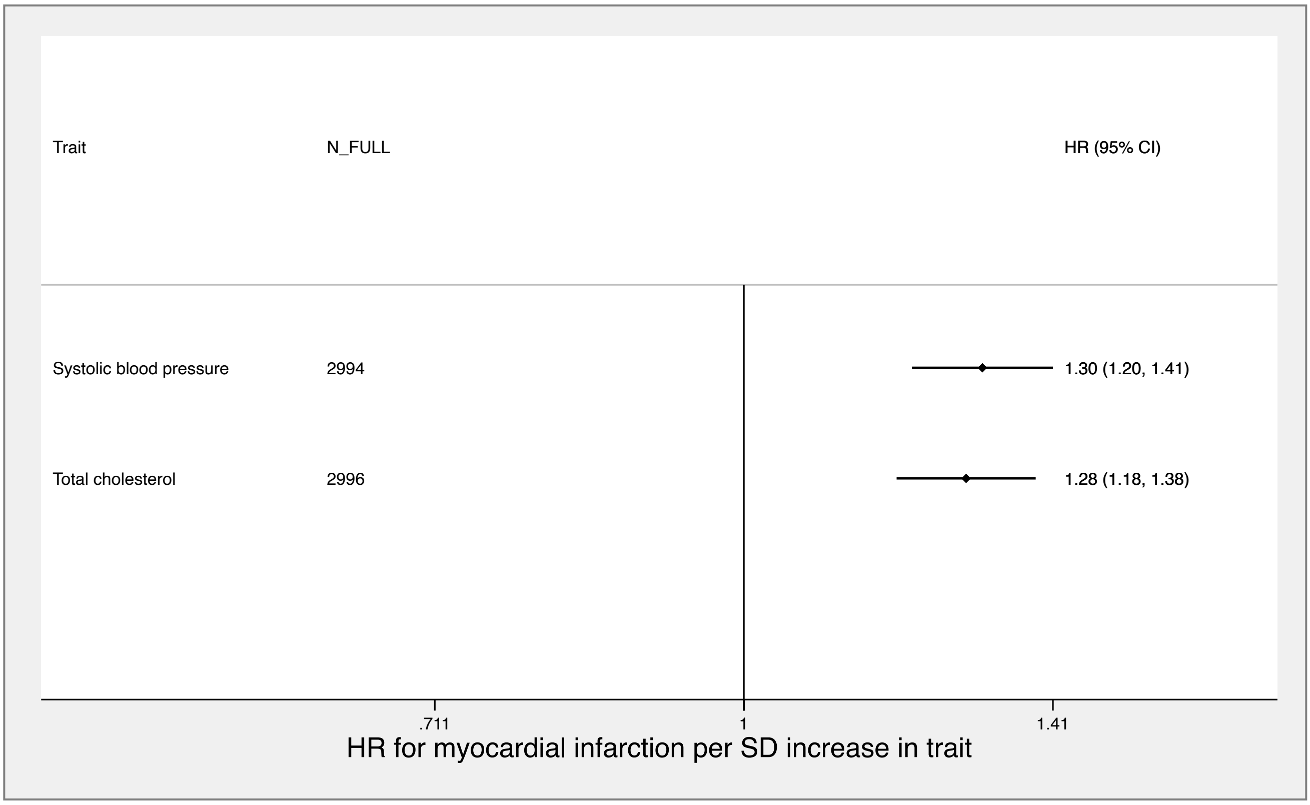

(B) Associations between systolic blood pressure (mmHg) and total cholesterol (mmol/L) and *F2RL3* DNA methylation (CpG_1) in the Copenhagen City Heart Study in the full sample as compared to the incident myocardial infarction (MI) cases only.

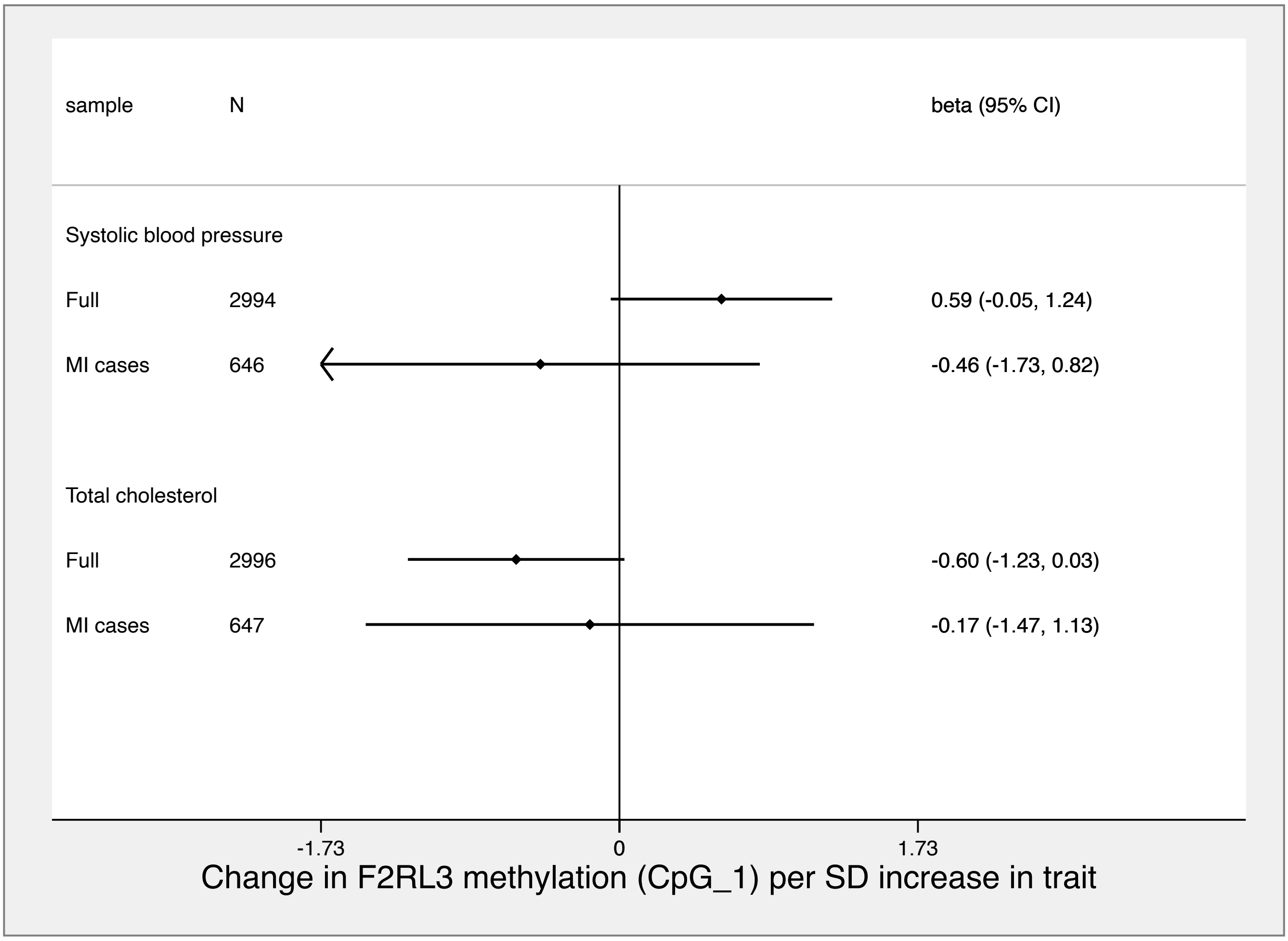

Supplementary Figure 3. Difference in mean methylation extent (%) between low (blue) and high (red) methylation groups in the recall study relative to the distribution of methylation extent in smokers, former smokers and never smokers

Data from the recall study are from the Avon Longitudinal Study of Parents and Children (ALSPAC). Data by smoking category are from the Copenhagen City Heart Study.

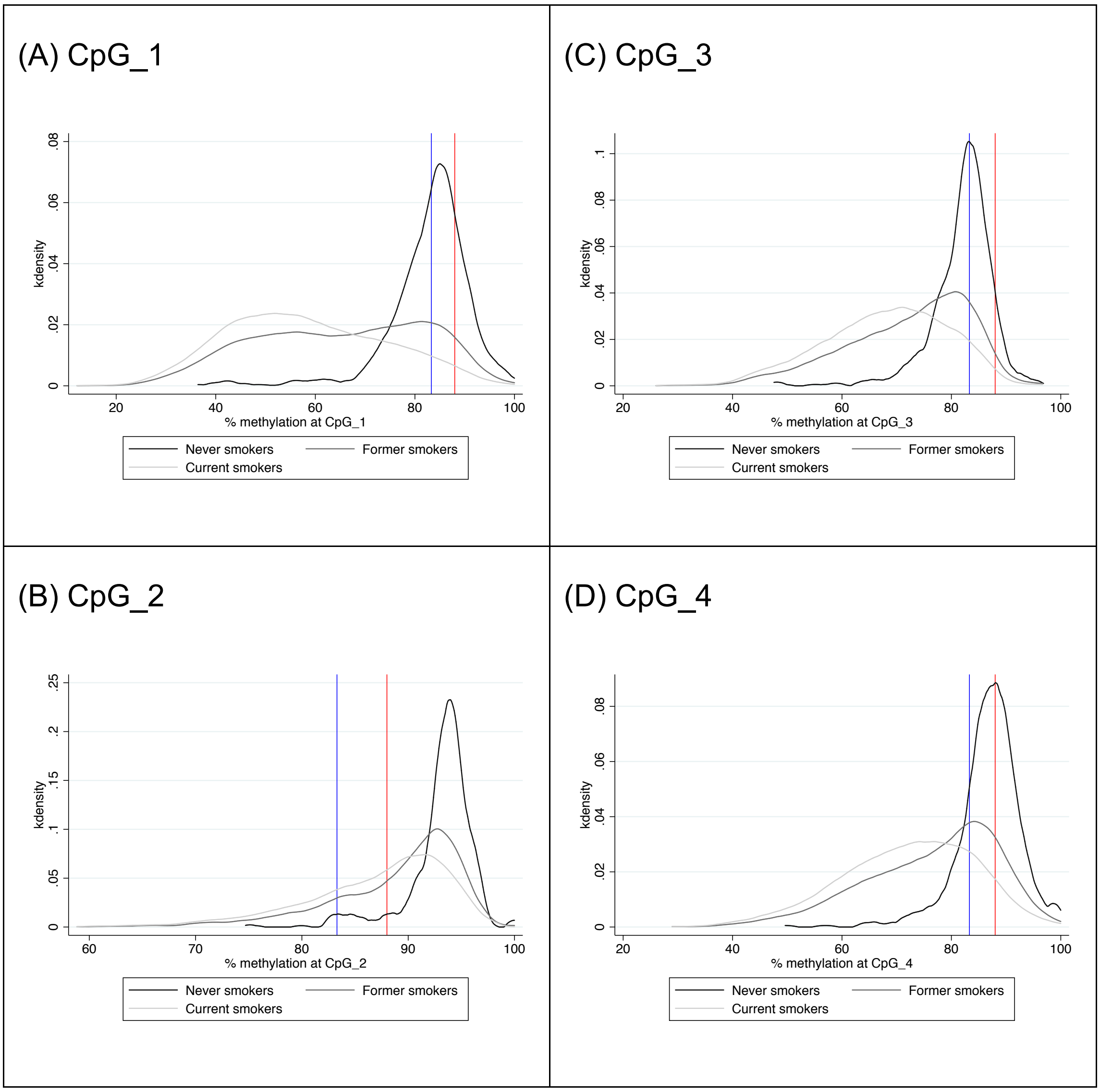

Supplementary Figure 4. Within methylation group effect of rs773902 genotype on PAR1-stimulated platelet reactivity measures

Boxplots showing the between group difference in $\alpha_{IIb}\beta_3$ integrin EC_{50} and α-granule P-selectin in response to PAR1-specific agonist peptide SFLLRN, by rs773902 genotype. In boxplots, upper and lower hinges of boxplots correspond to the first and third quartiles with the centre line indicating the median and whiskers extending from the hinge to the largest (smallest) value no further than 1.5 * IQR from the hinge.

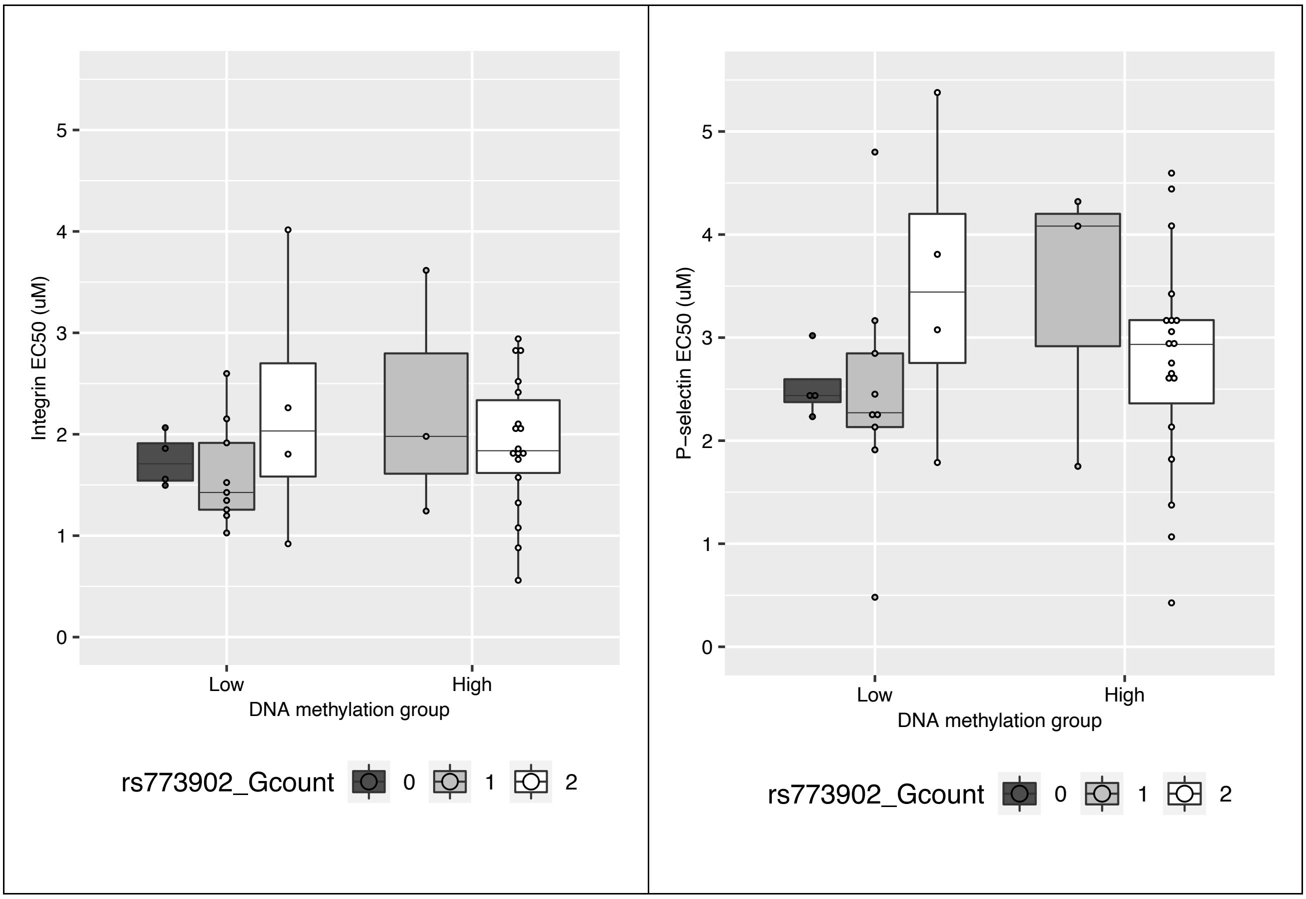

Supplementary Figure 5. Effect of cigarette smoke exposure (CSE) on *F2RL3* DNA methylation and mRNA expression in human coronary artery endothelial cells (HCAEC)

(A) Boxplot showing between treatment difference in methylation % (n=3). Control = no treatment; CSE = exposure of cells to cigarette smoke extract. Test results given for a two-sample two-sided *t*-test for a difference of means.

(B) Boxplot showing effect on *F2RL3* expression of exposure of HCAECs to 48 hours of CSE (n=6) compared to untreated control cells (n=4). Test results given for a two-sample *t*-test for a difference of means between CSE treated cells and untreated controls.

In boxplots, upper and lower hinges correspond to the first and third quartiles with the centre line indicating the median and whiskers extending from the hinge to the largest (smallest) value no further than 1.5 * IQR from the hinge. Source data are provided as a Source Data file.

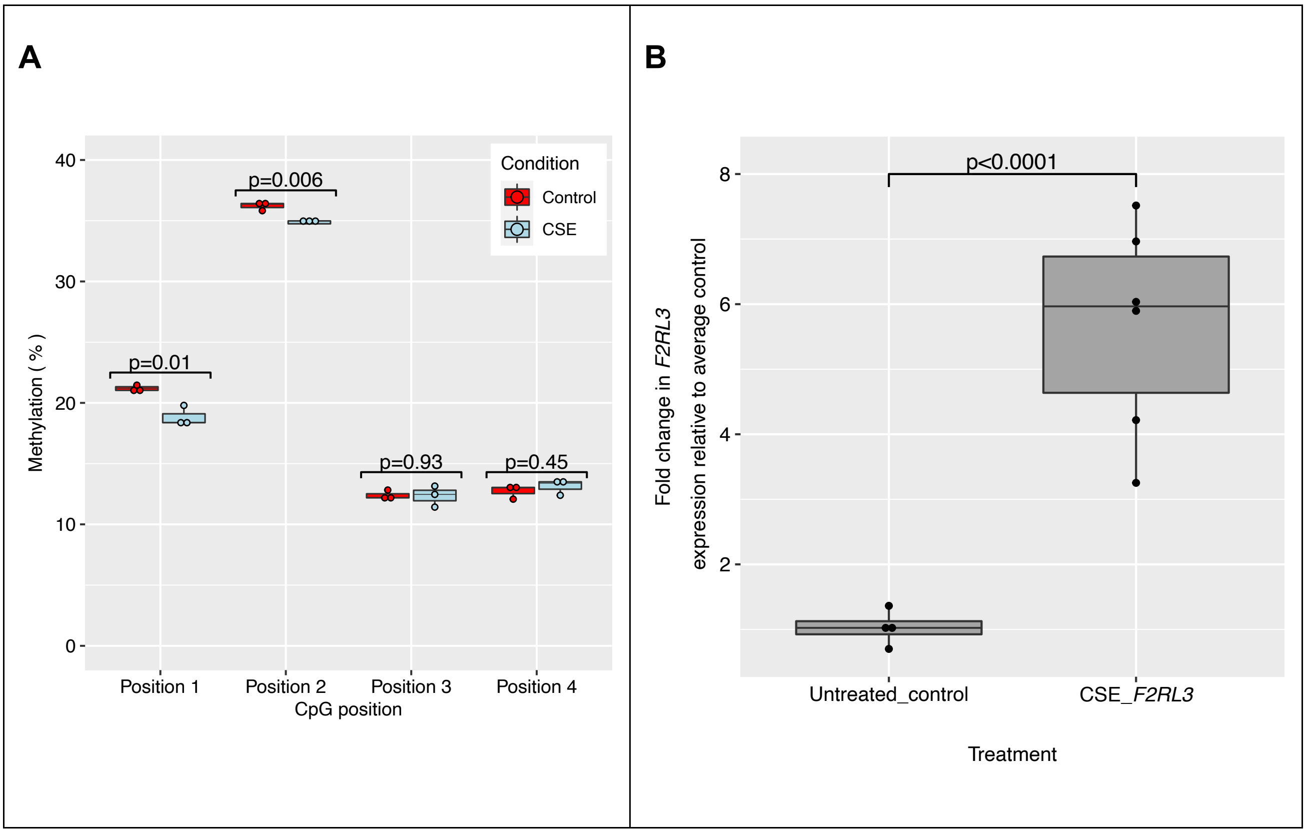

Supplementary Figure 6. Volcano plot showing differential protein abundance in CSE-treated cells compared to untreated control cells.

Based on the average of three independent experimental runs. Difference in protein abundance following CSE exposure as compared to control, expressed as log2 fold change (LogFC) (x-axis). P-value derived from a two-sample t-test (unequal variance) to compare mean abundance following CSE exposure as compared to control applied to log2-transformed data (-log10(p) on y-axis). Vertical dashed lines indicate a 1.5-fold change. Horizontal dashed line indicates p=0.05. Marked on the plot are two proteins: F2RL3 (PAR4) and RPLP0.

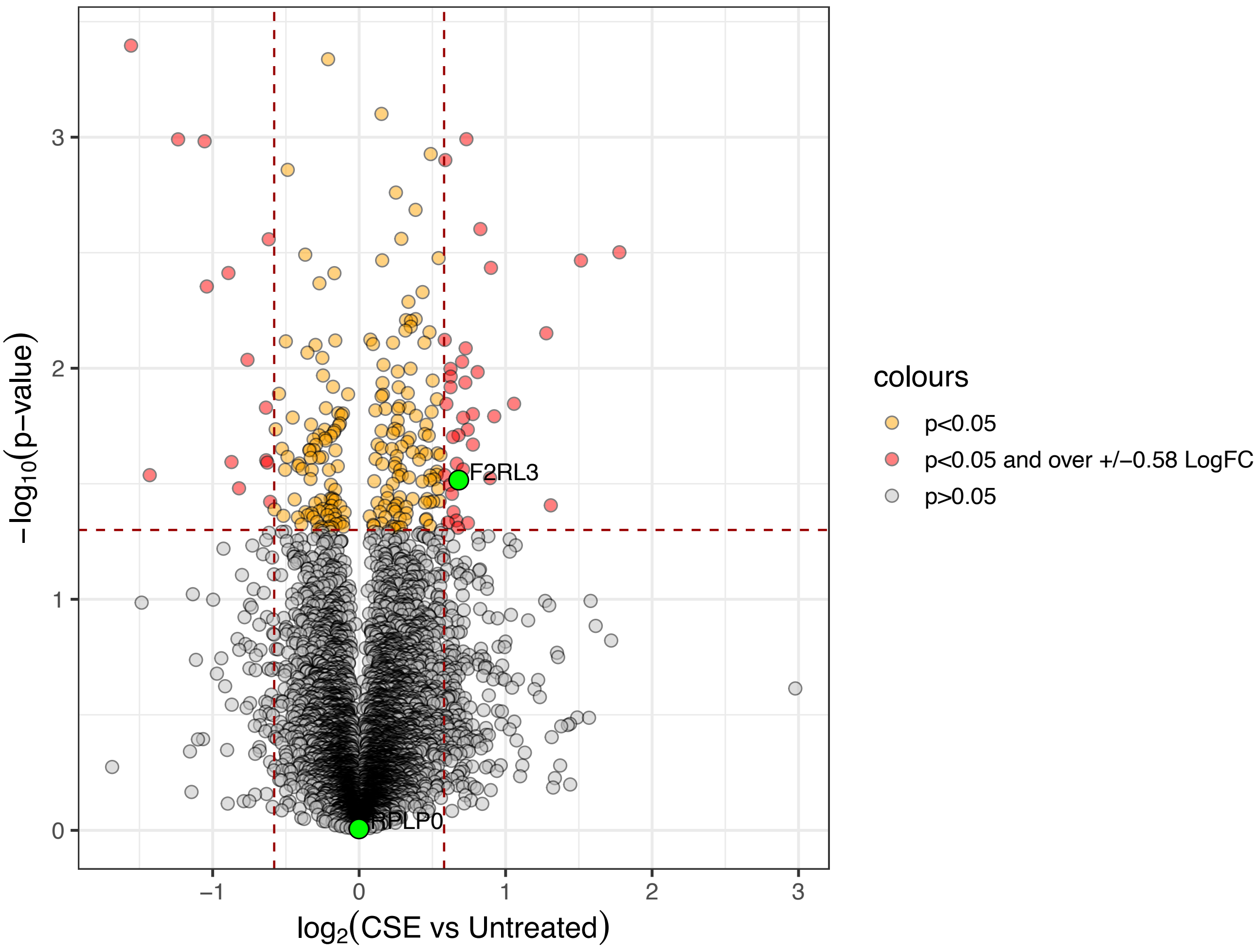

Supplementary Figure 7. Enhancer activity of the *F2RL3* exon 2 region in HEK-293 cells.

Boxplot showing *F2RL3* expression levels for different constructs. Basal activity of luciferase was observed with a pCpGL reporter vector containing the 2kB promoter region of *F2RL3* immediately upstream of the F2RL3 transcription start site (pCpGL_F2RL3pro; represented as the baseline level indicated by the dashed line at 1-fold). Insertion of the *F2RL3* exon 2 fragment alone into the pCpGL vector (pCpGL_exon2) resulted in low level (below baseline), but measurable luciferase activity. Luciferase activity was increased in a pCpGL reporter vector containing both the *F2RL3* promoter region and the exon 2 fragment (pCpGL_F2RL3pro_exon2). This enhancer effect of the *F2RL3* exon 2 fragment was abrogated by deletion of the CCAAT recognition site within the exon 2 fragment (pCpGL_F2RL3pro_exon2 CCAAT deletion). Upper and lower hinges of boxplots correspond to the first and third quartiles with the centre line indicating the median and whiskers extending from the hinge to the largest (smallest) value no further than 1.5 * IQR from the hinge. Test results are for one sample t-tests comparing expression to baseline where p-values appear directly above the bar and for a two-sample t-test comparing expression with and without the CCAAT deletion where p-value appears between bars. Source data are provided as a **Source Data** file.

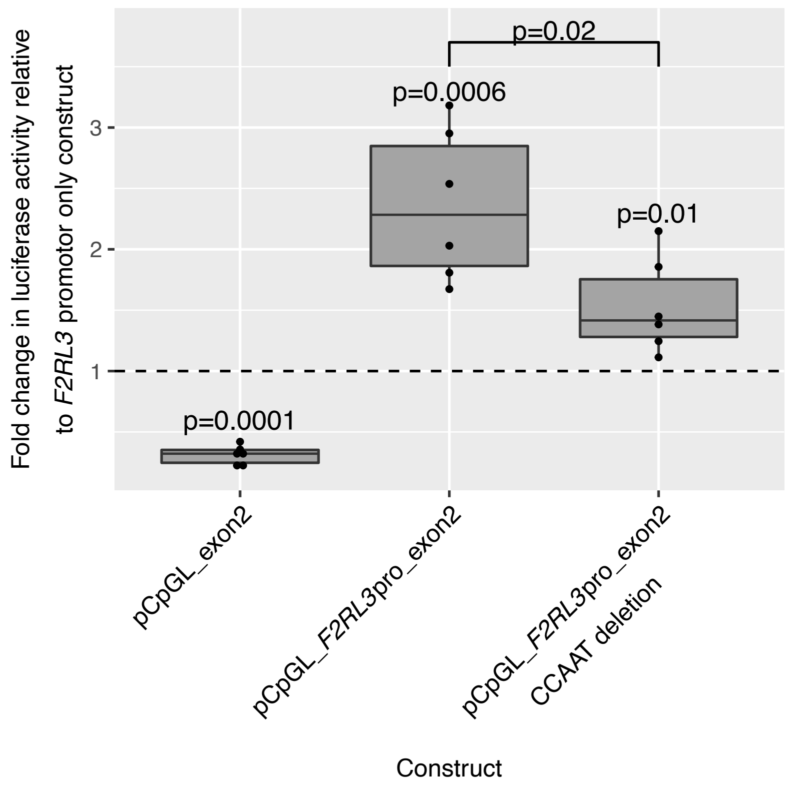

Supplementary Figure 8. RNA-Seq data showing gene expression of *F2RL3* in megakaryocyte cells as compared to other cell types.

Data extracted from the BLUEPRINT Data Analysis Portal[74] (data release 2016-08). FPKM = Fragments per kilobase million.

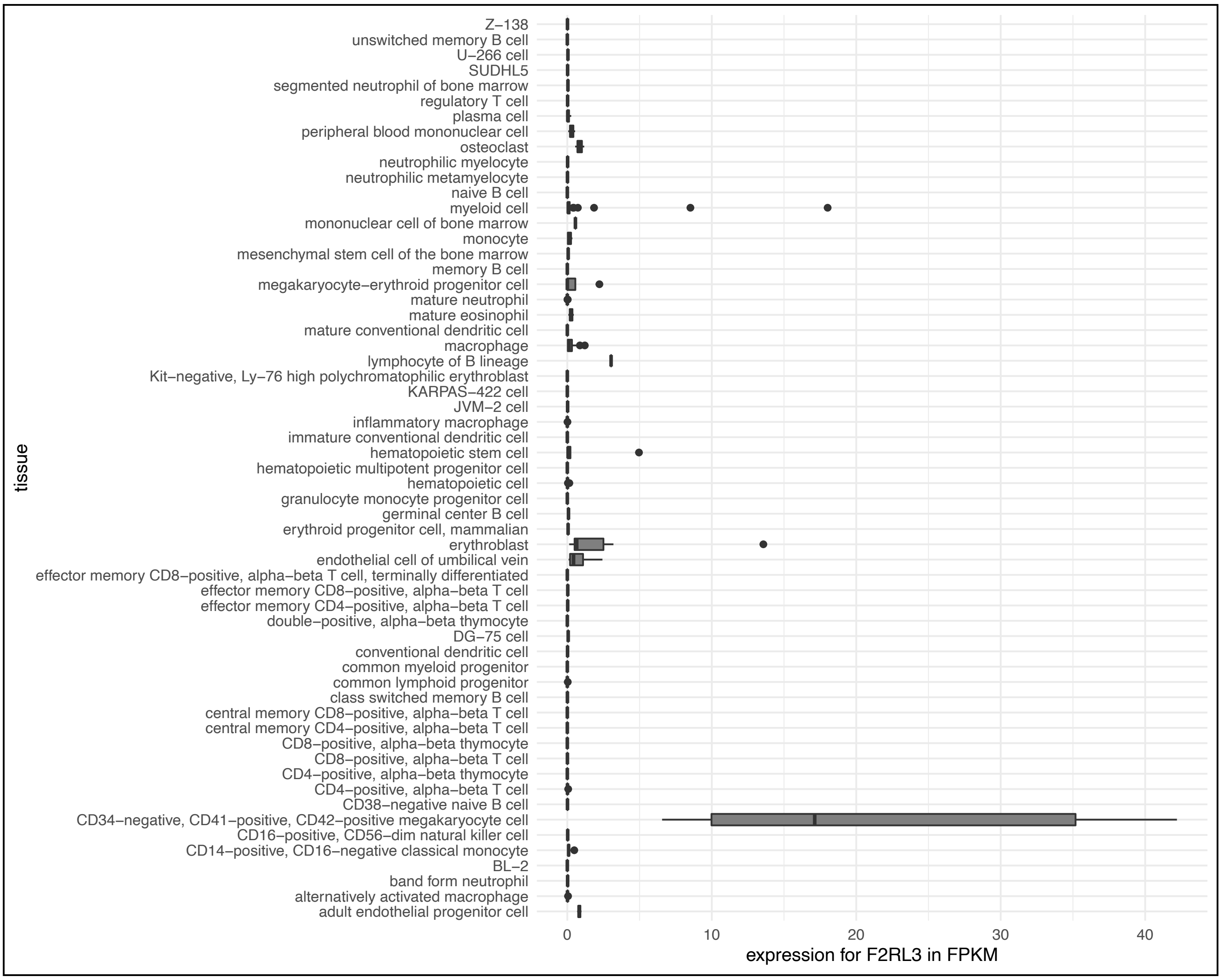

Supplementary Figure 9. RNA-Seq data showing transcript expression of *F2RL3* in megakaryocyte cells.

Data extracted from the BLUEPRINT Data Analysis Portal[74] (data release 2016-08). FPKM = Fragments per kilobase million.

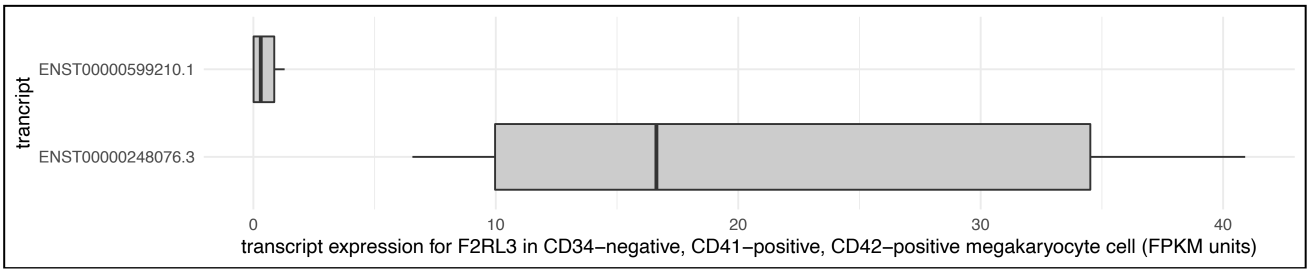

Supplementary Figure 10A. UCSC browser view of epigenetic annotations of *F2RL3*

UCSC browser view of *F2RL3* (chr19: 16,888,999-16,892,606 bp). Custom track 'study-specific features' (black) shows the four DNA methylation sites CpG_1 to CpG_4 that were assessed by pyrosequencing, rs773902 and a CCAAT binding factor recognition sequence. CpG_3 corresponds to the CpG labelled cg03636183 on the Illumina Infinium Human Methylation450 BeadChip (450K) array. Selected epigenetic annotations integrated from BLUEPRINT (data release 2016-08) based on data from a megakaryocyte cell lineage. Figure produced using UCSC Genome Browser[58] based on Genome Reference Consortium Human Build 38 (GRCh38/hg38).

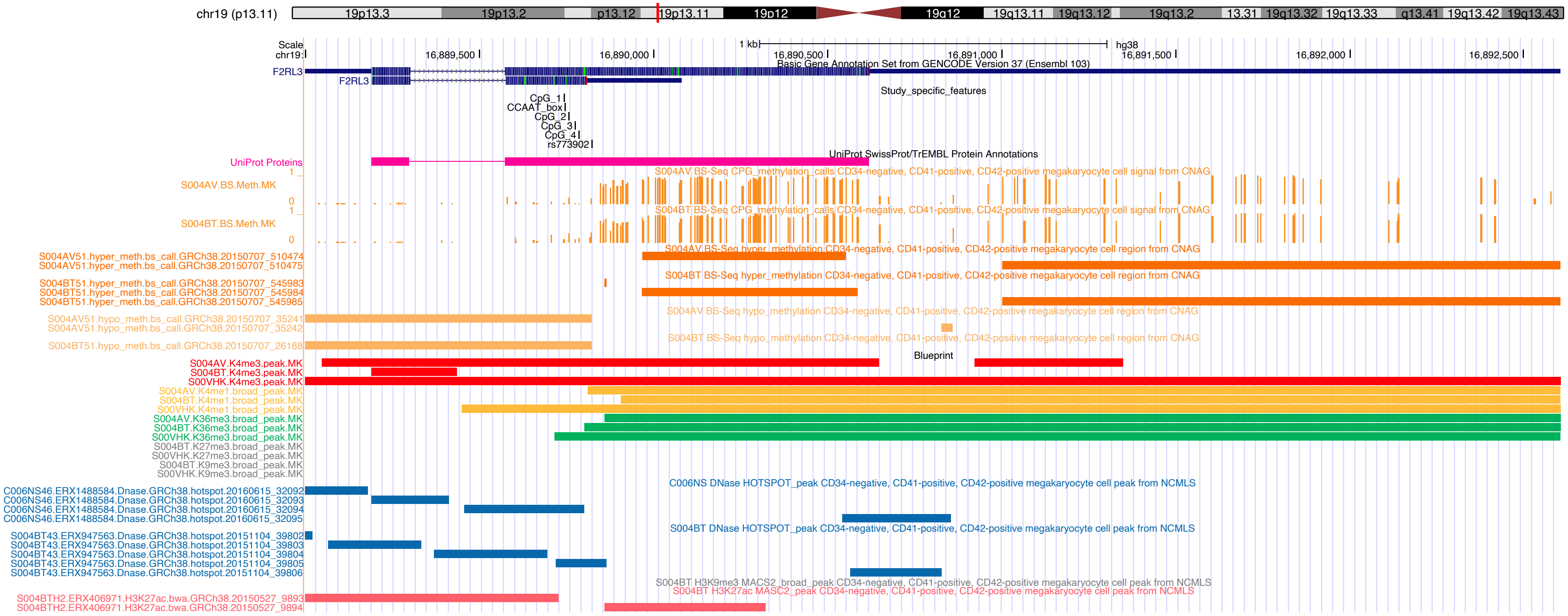

Supplementary Figure 10B Chromatin accessibility (DNAseI-Seq) at *F2RL3* in megakaryocytes as compared to other cell lineages

Data downloaded from the BLUEPRINT Data Analysis Portal (data release 2016-08)[35]. Z-scores presented were calculated using the Hotspot algorithm in which z-scores are calculated using a binomial distribution to find 'hotspot' regions. Further details of the DNAse-Seq analysis performed can be found at: http://dcc.blueprint-epigenome.eu/#/md/dnase_seq_grch38.

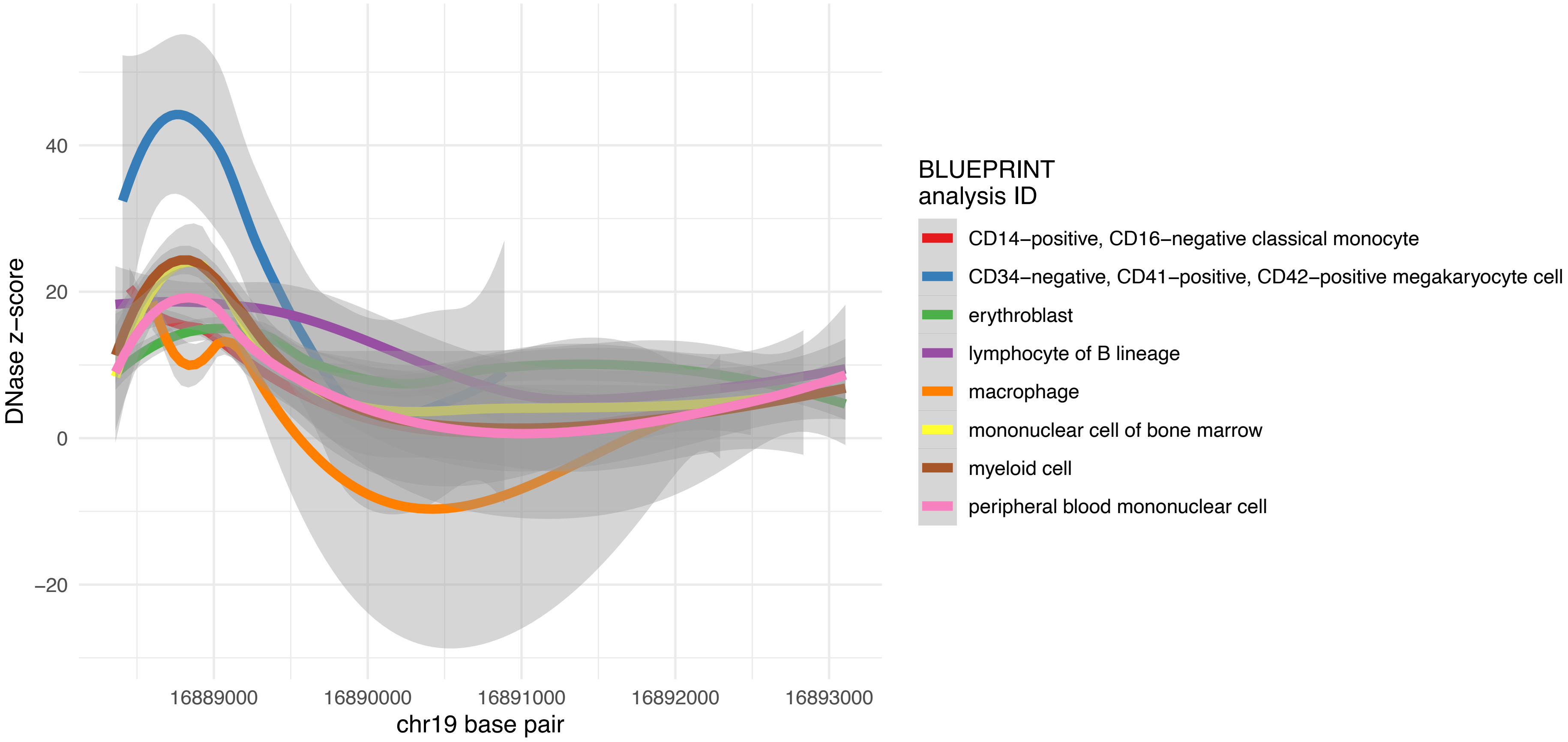

Supplementary Tables

Supplementary Table 1. Characteristics of study population in the Copenhagen City Heart Study

Data for all individuals with baseline data (N=9,234) and all individuals with samples sent for analysis as part of this study (N=4,292).

	All of wave 3 (N=9,234)		Selected for inclusion (N=4,292)	
	Sample size[a]	Mean (SD) / N (%)	Sample size[a]	Mean (SD) / N (%)
**Exposures**				
Smoking status	9,234		4,292	
Never		2,087 (22.6%)		789 (18.4%)
Former		2,660 (28.8%)		1,434 (33.4%)
Current		4,487 (48.6%)		2,069 (48.2%)
Smoking level				
Pack years (current/former smokers)	7,120	26 (13,40)[b]	3,493	31 (20, 45)[b]
Cigarettes per day (current smokers)	4,487	15 (10,20)[b]	2,069	15 (10, 20)[b]
AHRR DNA methylation intensity (%)	9,234	56.6 (9.2)	4,292	56.2 (9.1)
**Outcomes**				
Myocardial infarction (case count)	9,234	1,158 (12.5%)	4,292	1,125 (26.2%)
Incident myocardial infarction[c] (case count)	9,234	877 (9.5%)	4,292	844 (19.7%)
**Demographics/covariates**				
Age (years)	9,234	58.1 (15.2)	4,292	65.5 (11.1)
Sex (male)	9,234	5,114 (55.4%)	4,292	1,968 (45.9%)
Ancestry (Danish or other European count)	9,234	9,088 (98.4%)	4,292	4,256 (99.2%)
Statin use (case count)	9,234	84 (0.9%)	4,292	66 (1.5%)
Diabetes mellitus (case count)	9,234	167 (1.8%)	4,292	93 (2.2%)
Systolic blood pressure (mmHg)	9,225	138.6 (22.6)	4,288	144.8 (22.1)
Total cholesterol (mmol/L)	9,226	6.1 (1.3)	4,289	6.4 (1.2)
Exposed to passive smoking (exposed count)	9,234	3,321 (36.0%)	4,292	1,488 (34.7%)

N=sample size, SD=standard deviation. [a] Sample size is less than the total where there are missing data or summary statistics relate to a specific subset of individuals; [b] median (interquartile range) presented in place of mean (SD) in the case of skewed data distributions (Shapiro Wilk W-statistic < 0.9); [c] incident cases are those that occurred after the individual attended the baseline clinic (held between 1991-1994).

Supplementary Table 2A. Associations between smoking and *F2RL3* DNA methylation in the Copenhagen City Heart Study (N=3,205)

Results are from a linear regression model in which DNA methylation was the dependent variable, with age and sex fitted as covariates in all models. Measures of *F2RL3* DNA methylation were checked for normality using a Shapiro-Wilk test; W-statistics were: 0.95 for CpG_1, 0.87 for CpG_2, 0.94 for CpG_3 and 0.95 for CpG_4.

	% DNA methylation *F2RL3* CpG_1			% DNA methylation *F2RL3* CpG_2			% DNA methylation *F2RL3* CpG_3			% DNA methylation *F2RL3* CpG_4		
	beta (95% CI)	*p*	R2	beta (95% CI)	*p*	R2	beta (95% CI)	*p*	R2	beta (95% CI)	*p*	R2
Never (N=548)			0.36			0.20			0.29			0.28
Former (N=1,068)	-7.15 (-8.58, -5.71)	4.1×10^{-22}		-1.18 -1.75, -0.61)	5.2×10^{-5}		-3.23 (-4.21, -2.25)	1.1×10^{-10}		-3.50 (-4.56, -2.45)	9.1×10^{-11}	
Current (N=1,589)	-23.98 (-25.33, -22.62)	$<1.0 \times 10^{-100}$		-5.92 (-6.46, -5.38)	7.6×10^{-97}		-13.5 (-14.40, -12.55)	$<1.0 \times 10^{-100}$		-14.12 (-15.12, -13.13)	$<1.0 \times 10^{-100}$	
Pack years in ever smokers[a] (N=2,649)	-5.53 (-5.99, -5.07)	$<1.0 \times 10^{-100}$	0.19	-1.40 (-1.58, -1.22)	6.5×10^{-50}	0.09	-3.12 (-3.43, -2.81)	7.9×10^{-79}	0.14	-3.32 (-3.66, -2.98)	1.38×10^{-77}	0.14
Cigarettes per day in current smokers (N=1,589)	-0.28 (-0.36, -0.20)	5.2×10^{-12}	0.04	-0.10 (-0.13, -0.06)	6.1×10^{-8}	0.03	-0.17 (-0.23, -0.12)	3.3×10^{-9}	0.03	-0.20 (-0.26, -0.14)	2.6×10^{-10}	0.04
DNA methylation at *AHRR* (N=3,205)	1.24 (1.19, 1.29)	$<1.0 \times 10^{-100}$	0.44	0.33 (0.31, 0.35)	$<1.0 \times 10^{-100}$	0.25	0.72 (0.69, 0.76)	$<1.0 \times 10^{-100}$	0.35	0.76 (0.72, 0.80)	$<1.0 \times 10^{-100}$	0.34

N = sample size, R2 = overall model R-squared (includes age and sex contributions); [a] expressed as a doubling of pack years.

Supplementary Table 2B. Associations between time since cessation and *F2RL3* DNA methylation in former smokers in the Copenhagen City Heart Study (N=913)

All former smokers with information on time since cessation were included. Results are from a linear regression model in which DNA methylation was the dependent variable, with age and sex fitted as covariates.

CpG Site	Effect [a] (95% CI)	*p*	R2 (including age and sex)	R2 (excluding age and sex)
CpG_1	0.42 (0.35, 0.48)	$6.1x10^{-36}$	0.18	0.13
CpG_2	0.08 (0.06, 0.10)	$1.2x10^{-12}$	0.06	0.04
CpG_3	0.18 (0.14, 0.22)	$3.6x10^{-19}$	0.11	0.07
CpG_4	0.23 (0.19, 0.27)	$2.7x10^{-24}$	0.12	0.09

N = sample size, R2 = model R-squared; [a] Effect represents the change in *F2RL3* DNA methylation per additional year since smoking cessation, adjusting for age and sex.

Supplementary Table 3. Associations between *F2RL3* DNA methylation extent and myocardial infarction in the Copenhagen City Heart Study (N=2,998)

Results are from a stratified analysis within smoking status groups (current, former and never smokers). A Cox regression model was fitted in which myocardial infarction status was the dependent variable. Hazard ratios are per standard deviation (SD) decrease in DNA methylation. Individuals with myocardial infarction diagnosed prior to date of clinic at which blood for DNA methylation assay was taken were excluded from analysis (N=207). Results are shown for the fully adjusted model with age, sex, *AHRR* methylation, diabetes, systolic blood pressure, total cholesterol and passive smoking fitted as covariates.

Exposure: *F2RL3* DNA methylation (per SD decrease)			Hazard ratio (95% CI), *p*			
	N [a]	N events	CpG_1	CpG_2	CpG_3	CpG_4
Current smokers	1,490	379	1.27 (1.11,1.46), $p=5.8\times10^{-4}$	1.10 (1.00, 1.21), p=0.05	1.16 (1.04, 1.31), $p=8.9\times10^{-3}$	1.09 (0.98, 1.23), p=0.13
Previous smokers	977	184	1.31 (1.07, 1.61), $p=8.6\times10^{-3}$	0.94 (0.75, 1.19), p=0.60	1.02 (0.81, 1.27), p=0.90	1.09 (0.88, 1.33), p=0.43
Never smokers	525	82	0.99 (0.59, 1.69), p=0.98	0.98 (0.61, 1.58), p=0.93	0.82 (0.49, 1.37), p=0.44	1.31 (0.82, 2.10), p=0.26

[a] Sample sizes are slightly smaller than in Table 2 (in main manuscript) because a small number of individuals were not included in Model 3 due to missing covariate data.

Supplementary Table 4A. Associations between *F2RL3* DNA methylation extent at CpG_1 and mortality in individuals experiencing a myocardial infarction in the Copenhagen City Heart Study (N=648)

Results are from a stratified analysis within smoking status groups (current, former and never smokers). A Cox regression model was fitted in which myocardial infarction mortality status was the dependent variable. Hazard ratios are per standard deviation (SD) decrease in DNA methylation. Individuals with myocardial infarction diagnosed prior to date of clinic at which blood for DNA methylation assay was taken were excluded from analysis (N=207). See **Supplementary Table 4B** for results for CpG sites CpG_2, CpG_3 and CpG_4.

Exposure: *F2RL3* DNA methylation (per SD decrease)			Hazard ratio (95% CI), *p*		
	N[a]	N events	Model 1: Age, sex	Model 2: Model 1 plus *AHRR* DNA methylation	Model 3: Model 2 plus diabetes, SBP, total cholesterol and passive smoking
Current smokers	379	299	1.38 (1.20, 1.59), p=9.5x10^{-6}	1.31 (1.10, 1.56), p=2.6x10^{-3}	1.31 (1.10. 1.56), p=2.8x10^{-3}
Previous smokers	186	145	1.20 (0.98, 1.47), p=0.08	1.19 (0.94, 1.51), p=0.14	1.20 (0.94, 1.53), p=0.14
Never smokers	83	64	0.96 (0.60, 1.53), p=0.86	0.96 (0.60, 1.55), p=0.88	1.04 (0.63, 1.73), p=0.87

[a] Sample size based on Model 1. A small number of individuals (up to four) were not included in Model 3 due to missing covariate data (see **Supplementary Table 4B** for Model 3 sample sizes).

Supplementary Table 4B. Associations between *F2RL3* DNA methylation extent (all CpGs) and mortality in individuals experiencing a myocardial infarction in the Copenhagen City Heart Study (N=648)

Results are from a stratified analysis within smoking status groups (current, former and never smokers). A Cox regression model was fitted in which myocardial infarction mortality status was the dependent variable. Models adjusted for age, sex, *AHRR* DNA methylation, diabetes, SBP, total cholesterol and passive smoking (as Model 3 in **Supplementary Table 4A**).

Exposure: *F2RL3* DNA methylation (per SD decrease)			Hazard ratio (95% CI), *p*			
	N [a]	N events	CpG_1	CpG_2	CpG_3	CpG_4
Current smokers	379	299	1.31 (1.10. 1.56), p=2.8x10^{-3}	1.16 (1.03, 1.30), p=0.01	1.20 (1.04, 1.38), p=0.01	1.19 (1.03, 1.37), p=0.02
Previous smokers	184	143	1.20 (0.94, 1.53), p=0.14	1.27 (0.93, 1.74), p=0.14	1.24 (0.95, 1.63), p=0.12	1.15 (0.89, 1.47), p=0.28
Never smokers	82	64	1.04 (0.63, 1.73), p=0.87	1.50 (0.93, 2.43), p=0.10	1.21 (0.76, 1.93), p=0.43	1.70 (1.10, 2.64), p=0.02

[a] Sample sizes are slightly smaller than in **Supplementary Table 4A** because a small number of individuals were not included in Model 3 due to missing covariate data.

Supplementary Table 5. Mediation analysis in ever smokers from smoking to myocardial infarction (N=2,461) and death after a myocardial infarction (N=561)

Results are from a mediation analysis in which the proportion mediated was estimated by the product of coefficients method[23], using:

$$\frac{coefficient\ a \times coefficient\ b}{(coefficient\ a \times coefficient\ b) + coefficient\ c'},$$

where coefficient 'a' represents the relation of exposure (smoking) to mediator (methylation), coefficient 'b' represents the relation of mediator (methylation) to outcome (myocardial infarction or death after myocardial infarction) adjusted for exposure (smoking) and coefficient 'c′' represents the relation of exposure (smoking) to outcome (myocardial infarction or death after myocardial infarction) adjusted for mediator (methylation). All analyses were adjusted for age, sex, diabetes status, systolic blood pressure, total cholesterol, passive smoking and smoking status (former/current). Smoking is expressed as doubling of pack years and those missing these data excluded (N=6).

**Analysis**	**N (cases/ events)**	**Linear regression**	**Cox regression of methylation marker, adjusted for smoking**		**Cox regression of smoking, adjusted for methylation marker**		**Estimated proportion mediated (%)**
		Coefficient a (95%CI)	Hazard ratio (95%CI)	Coefficient b (95%CI)	Hazard ratio (95%CI)	Coefficient c′ (95%CI)	
Myocardial infarction							
DNA methylation at *F2RL3* CpG_1 (per SD decrease)	2461 (561)	0.22 (0.19, 0.25)	1.26 (1.14, 1.39)	0.23 (0.13, 0.33)	1.11 (1.02, 1.20)	0.10 (0.02, 0.19)	34%
DNA methylation at *AHRR* (per SD decrease)		0.18 (0.16, 0.21)	1.15 (1.03,1.30)	0.14 (0.03, 0.26)	1.14 (1.05, 1.24)	0.13 (0.05, 0.21)	16%
Death after myocardial infarction							
DNA methylation at *F2RL3* CpG_1 (per SD decrease)	561 (441)	0.24 (0.17, 0.31)	1.27 (1.13, 1.43)	0.24 (0.12, 0.36)	1.09 (0.98, 1.21)	0.09 (-0.02, 0.19)	39%
DNA methylation at *AHRR* (per SD decrease)		0.15 (0.09, 0.20)	1.18 (1.03, 1.35)	0.17 (0.03, 0.30)	1.13 (1.02, 1.25)	0.12 (0.02, 0.22)	18%

CI = confidence interval.

Supplementary Table 6. Characteristics of ALSPAC participants selected for invite (n=200)

	Low DNA methylation (N=100)		High DNA methylation (N=100)		W-statistic[a]	Variance ratio test, *p* [b]	Between group difference, *p* [c]
	N	Mean (SD)	N	Mean (SD)			
**Selection criteria**							
F2RL3 DNA methylation [d] (%), age 7 clinic	100	65.3 (3.9) %	100	74.2 (3.7) %	0.99	0.68	8.2×10^{-40}
F2RL3 DNA methylation [d] (%), age 15/17 clinic	100	62.9 (4.1) %	100	71.5 (4.3) %	0.99	0.69	8.7×10^{-33}
**Potential confounders relevant to cardiovascular health**							
Sex (% male)	100	54%	100	49%	n/a	n/a	0.48 [e]
Age (years)	99	23.0 (0.5)	100	23.2 (0.5)	0.95	0.68	0.05
Maternal education (% with A-level/Degree)	99	46.5%	99	52.5%	n/a	n/a	0.39 [e]
Body mass index (kg/m^2), age 17 clinic	88	22.3 (3.9)	87	22.9 (3.4)	0.92	0.23	0.33
Systolic blood pressure (mmHg), age 17 clinic	86	115.7 (9.0)	86	114.2 (9.2)	0.99	0.81	0.29
Diastolic blood pressure (mmHg), age 17 clinic	86	64.1 (6.0)	86	64.6 (5.5)	0.99	0.41	0.60
C-reactive protein (mg/L), age 17 clinic	81	0.46 (0.22, 1.66) [f]	78	0.54 (0.27, 1.25) [f]	0.56	1.0×10^{4}	0.58 [g]
Cholesterol (mmol/L), age 17 clinic (fasting)	81	3.73 (0.63)	78	3.74 (0.78)	0.95	0.06	0.92
**Biomarkers for exposure to cigarette smoke**							
Cotinine (ng/mL), age 15 clinic	74	0.87 (0.31, 1.27) [f]	84	0.76 (0.16,1.12) [f]	0.14	$< 1.0 \times 10^{4}$	0.68 [g]
AHRR DNA methylation [e] (%), age 7 clinic	100	82.8 (3.3) %	100	83.3 (3.6) %	0.98	0.43	0.37
AHRR DNA methylation [e] (%), age 15/17 clinic	100	81.7 (3.9) %	100	81.9 (3.8) %	0.98	0.72	0.70
**Genetic**							
Minor allele (A) frequency at rs773902 (%)	100	33.5%	100	8.0%	n/a	n/a	3.21×10^{-10} [e]

[a] W-statistic from a Shapiro-Wilk test of normality performed on data from all invited participants (n=200); [b] *p*-value from a two-sided test on the equality of standard deviations (variances); [c] *p*-value from two-sample two-sided t-test assuming equal variances (unless stated otherwise); [d] DNA methylation at cg03636183 assessed by Illumina Infinium Human Methylation 450K BeadChip assay and presented as raw betas (proportion methylated) x 100 to give % DNA methylation; [e] *p*-value from a Pearson's chi-squared test; [f] summary presented as 'median (interquartile range)' because distribution does not approximate a normal distribution (W-statistic <0.90); [e] DNA methylation at cg05575921

assessed by Illumina Infinium Human Methylation 450K BeadChip assay and presented as raw betas (proportion methylated) x 100 to give % DNA methylation; [g] *p*-value from a Wilcoxon rank-sum (Mann-Whitney) test because distribution does not approximate a normal distribution (W-statistic <0.90).

Supplementary Table 7. Comparison of recruited sample of ALSPAC participants (n=41) and all those selected for invite (n=200)

	Not recruited (N=159)		Recruited (N=41)		Between group difference, *p* [a]
	N	Mean (SD)	N	Mean (SD)	
**Selection criteria**					
F2RL3 DNA methylation [b] (%), age 7 clinic	159	69.8 (5.8) %	41	69.7 (6.3) %	0.95
F2RL3 DNA methylation [b] (%), age 15/17 clinic	159	66.8 (5.9) %	41	68.8 (6.6) %	0.06
**Potential confounders relevant to cardiovascular health**					
Sex (% male)	159	54%	41	41%	0.15 [c]
Age (years)	158	23.1 (0.5)	41	23.1 (0.5)	0.44
Maternal education (% with A-level/Degree)	157	48.4%	41	53.7%	0.55 [c]
Body mass index (kg/m^2), age 17 clinic	137	22.7 (3.6)	38	22.3 (3.8)	0.56
Systolic blood pressure (mmHg), age 17 clinic	134	115.1 (9.0)	38	114.7 (9.5)	0.81
Diastolic blood pressure (mmHg), age 17 clinic	134	64.1 (5.5)	38	65.1 (6.6)	0.35
C-reactive protein (mg/L), age 17 clinic	123	0.58 (0.24,1.46) [d]	36	0.38 (0.25, 0.82) [d]	0.44 [f]
Cholesterol (mmol/L), age 17 clinic (fasting)	123	3.72 (0.74)	36	3.77 (0.59)	0.72
**Biomarkers for exposure to cigarette smoke**					
Cotinine (ng/mL), age 15 clinic	125	0.88 (0.29, 1.19) [d]	33	0.71 (0.35, 0.93) [d]	0.20 [f]
AHRR DNA methylation [e] (%), age 7 clinic	159	83.2 (3.5) %	41	82.7 (3.2) %	0.42
AHRR DNA methylation [e] (%), age 15/18 clinic	159	81.7 (3.9) %	41	82.3 (3.8) %	0.42
**Genetic**					
Minor allele (A) frequency at rs773902 (%)	159	19.5%	41	25.6%	0.22 [c]

[a] *p*-value from two-sample two-sided t-test assuming equal variances (unless stated otherwise); [b] DNA methylation at cg03636183 assessed by Illumina Infinium Human Methylation 450K BeadChip assay and presented as raw betas (proportion methylated) x 100 to give % DNA methylation; [c] *p*-value from a Pearson's chi-squared test; [d] summary presented as 'median (interquartile range)' because distribution does not approximate a normal distribution (W-statistic <0.90); [e] DNA methylation at cg05575921 assessed by Illumina Infinium Human Methylation 450K BeadChip assay and presented as raw betas (proportion methylated) x 100 to give % DNA methylation; [f] *p*-value from a Wilcoxon rank-sum (Mann-Whitney) test because distribution does not approximate a normal distribution (W-statistic <0.90).

Supplementary Table 8. Correlation across CpG sites in *F2RL3* of contemporary DNA methylation assessed by targeted pyrosequencing

	CpG_1	CpG_2	CpG_3	CpG_4
CpG_1				
CpG_2	0.50			
CpG_3	0.80	0.41		
CpG_4	0.69	0.61	0.72	

Supplementary Table 9. By-group comparison of contemporary DNA methylation and platelet reactivity measures as assessed by flow cytometry

Assay	Measure	Low DNA methylation (N=19) [a]		High DNA methylation (N=22) [a]		W-statistic [b]	Variance ratio test, p [c]	Between group difference, p [d]
		N	Mean (SD)	N	Mean (SD)			
DNA methylation (by pyrosequencing)	CpG_1 (%)	17	83.2 (3.3)	16	88.0 (3.9)	0.97	0.57	5.6×10^{-4}
	CpG_2 (%)	18	91.4 (0.6)	17	91.9 (0.8)	0.95	0.20	0.04
	CpG_3 (%)	18	82.1 (1.8)	17	84.9 (1.9)	0.97	0.98	9.9×10^{-5}
	CpG_4 (%)	17	83.4 (2.1)	13	85.1 (2.3)	0.98	0.80	0.04
Stimulation by PAR4 agonist (AYPGKF) [e]	Integrin activation, EC_{50} (μM)	19	60.8 (12.0)	22	80.1 (20.5)	0.92	0.03	8.9×10^{-4}
	Integrin activation (Max. response) (MFI)	19	4,480 (1,005)	22	4,634 (1,128)	0.97	0.63	0.65
	P-selectin exposure, EC_{50} (μM)	18	96.7 (25.1)	21	119.4 (38.6)	0.91	0.08	0.04
	P-selectin exposure, Max. response (MFI)	19	3,711 (1390)	22	3,847 (1,191)	0.98	0.49	0.74
Surface receptor expression [e]	CD41 (MFI)	19	23,720 (8,195)	22	23,216 (9,482)	0.96	0.54	0.86
	CD61 (MFI)	19	14,511 (2,662)	22	14,312 (3,793)	0.97	0.13	0.85
Stimulation by PAR1 agonist (SFLLRN) [e]	Integrin activation, EC_{50} (μM)	17	1.79 (0.73)	21	1.95 (0.74)	0.95	0.99	0.50
	Integrin activation (Max. response) (MFI)	19	3,888 (1,192)	22	3,906 (962)	0.97	0.34	0.96
	P-selectin exposure, EC_{50} (μM)	17	2.73 (1.14)	22	2.84 (1.11)	0.98	0.88	0.76
	P-selectin exposure, Max. response (MFI)	19	2,900 (1,194)	22	3,523 (1,211)	0.98	0.96	0.11

EC_{50} = half maximum concentration values (the concentration of agonist required to give half the maximal response); MFI = (geometric) mean fluorescence intensity; [a] sample size, *N*, for each measure may be less than the total *N* due to assay failures (in the case of DNA methylation) and data points being excluded during quality control (in the case of platelet measures); [b] W-statistic from a Shapiro-Wilk test of normality; [c] *p*-value from a two-sided test on the equality of standard deviations (variances); [d] *p*-value from two-sample two-sided t-test assuming equal variances; [e] Comparisons made after the removal of outliers identified by robust regression of outcome measure on DNA methylation group (high/low).

Supplementary Table 10A. By-group comparison of potential confounders of the relationship between DNA methylation and platelet reactivity in the recall study conducted in ALSPAC (N=41)

Category	Measure	Low DNA methylation (N=19) [a]		High DNA methylation (N=22) [a]		P-value [b]
		N	Mean (SD)	N	Mean (SD)	
Demographic	Sex (% male)	19	37%	22	45%	0.31 [c]
	Age (years)	19	23.2 (0.6)	22	23.1 (0.2)	0.81
	Maternal education (% with A-level/Degree)	19	38.8%	22	68.2%	0.05 [c]
Health-related traits	Body mass index (kg/m^2), age 17 clinic	18	22.8 (4.2)	20	21.8 (3.5)	0.46
	Systolic blood pressure (mmHg), age 17 clinic	19	116.1 (7.5)	19	113.2 (11.3)	0.36
	Diastolic blood pressure (mmHg), age 17 clinic	19	65.8 (7.6)	19	64.5 (5.7)	0.55
	C-reactive protein (mg/L), age 17 clinic	18	0.45 (0.29, 1.93) [d]	18	0.36 (0.24, 0.76) [d]	0.49
	Cholesterol (mmol/L), age 17 clinic (fasting)	18	3.89 (0.66)	18	3.65 (0.50)	0.24
Biomarkers for exposure to cigarette smoke	*AHRR* DNA methylation [f] (%), age 7 clinic	19	82.0 (3.0)	22	83.2 (3.3)	0.25
	AHRR DNA methylation [f] (%), age 15/17 clinic	19	82.2 (2.5)	22	82.3 (4.6)	0.90
	Cotinine (ng/mL), age 15 clinic	17	0.71 (0.31, 1.03) [d]	16	0.60 (0.36, 0.89) [d]	0.70
Haematology [g]	WBC (10^3/mm^3)	18	5.06 (1.09)	22	5.04 (1.23)	0.97
	RBC (10^3/mm^3)	19	4.18 (0.29)	22	4.10 (0.29)	0.34
	PLT (10^3/mm^3)	19	194.3 (38.6)	22	210.8 (47.0)	0.23
	MPV (μm^3)	19	7.75 (0.74)	22	7.62 (0.64)	0.55
Genetic	Minor allele (A) frequency at rs773902 (%)	19	47.4%	22	6.8%	2.7×10^{-5} [c]
Technical	Laboratory technician (% technician 1) [h]	19	57.9%	22	27.3%	0.05 [c]
	Time to assay (minutes) [i]	19	43 (28, 48) [d]	22	30 (25, 33) [d]	0.03

WBC = white blood cells; RBC = red blood cells; PLT = platelets; MPV = mean platelet volume; [a] sample size, *N*, for each measure may be less than the total *N* due to data points being excluded during quality control (in the case of haematology measures) or due to non-attendance of individuals at specific clinics; [b] *p*-value from two-sample two-sided t-test assuming equal variances (unless stated otherwise); [c] *p*-value from a Pearson's chi-squared test; [d] summary presented as 'median (interquartile range)' because distribution does not approximate a normal distribution (W-statistic <0.90); [e] *p*-value from a Wilcoxon rank-sum (Mann-Whitney) test because distribution does not approximate a normal distribution (W-statistic <0.90); [f] DNA methylation at cg05575921 assessed by Illumina Infinium Human Methylation 450K BeadChip assay and presented as raw betas (proportion methylated) x 100 to give % DNA methylation; [g] Comparisons made after the removal of outliers identified by robust regression of outcome measure on DNA methylation group (high/low); [h] Analyses were performed by two laboratory technicians; [i] Represents the time lag between blood draw and the start of the laboratory analysis (sample put into haematology analyser). In cases where the blood draw time was not recorded, draw time was predicted based on the mean time from appointment start time to blood draw (9 minutes).

Supplementary Table 10B. Linear regression to explore effect of covariates associated with methylation group on platelet reactivity measures (N=41)

Results provided for a univariate model (methylation group as the only independent variable) and a multivariable model (methylation group and potential confounder fitted).

Outcome [a]	Confounder(s)	Methylation group effect in univariable model			Methylation group effect in multivariable model			Confounder effect in multivariable model			Adjusted R^2 of multivariable model (%)
		Beta (SE)	p	% VE[b]	Beta (SE)	p	% VE[b]	Beta (SE)	p	% VE[b]	
Integrin activation, EC_{50} (μM)	Maternal education [c]	0.27 (0.07)	5.2×10^{-04}	26.9%	0.35 (0.06)	2.5×10^{-06}	35.9%	-0.26 (0.06)	2.2×10^{-4}	19.5%	46.5%
	Technician [d]				0.27 (0.07)	8.4×10^{-04}	25.7%	-0.02 (0.08)	0.813	0.11%	23.1%
	Time to assay (mins)				0.24 (0.08)	0.003	20.9%	-2.2×10^{-03} (2.9×10^{-03})	0.452	1.2%	24.2%
	rs773902 [e]				0.12 (0.08)	0.150	4.4%	-0.18 (0.06)	0.004	18.7%	38.1%
	All above				0.23 (0.09)	0.012	12.4%	n/a	n/a	n/a	48.4%
P-selectin exposure, EC_{50} (μM)	Maternal education [c]	0.20 (0.09)	0.035	11.5%	0.26 (0.09)	0.005	17.2%	-0.23 (0.09)	0.015	12.7%	20.9%
	Technician [d]				0.23 (0.10)	0.020	13.7%	-0.10 (0.10)	0.291	2.7%	9.5%
	Time to assay (mins)				0.14 (0.10)	0.154	5.3%	-5.0×10^{-03} (3.6×10^{-03})	0.177	4.7%	11.3%
	rs773902 [e]				0.06 (0.11)	0.614	0.6%	-0.17 (0.08)	0.056	9.7%	15.8%
	All above				0.14 (0.13)	0.30	2.8%	n/a	n/a	n/a	21.3%

[a] outliers identified by robust regression of outcome measure on DNA methylation group (high/low) then removed and remaining values natural log transformed; [b] variance explained (VE) calculated as the sums of squares allocated to the model component as a proportion of the total sums of squares (using output from a Type II ANOVA applied to the model output[65]); [c] binary factor coded such that women from the categories CSE/none, vocational and O level were coded 0, and those from the categories A level and Degree were coded 1; [d] binary factor coded to indicate technician 1 or 2; [e] genotype coded as minor (A) allele carriage (0,1,2), assuming an additive genetic model.

Supplementary Table 11. Linear regression to explore association of rs773902 with red blood cell count (N=41)

**Outcome** [a]	**Independent variable**	**Beta (SE)**	**p**	**Model R^2**
Red blood cell count	rs773902 [b]	0.04 (0.02)	0.009	17.8%
Integrin activation, EC_{50} (μM)	Red blood cell count	-0.30 (0.13)	0.030	11.6%
P-selectin exposure, EC_{50} (μM)	Red blood cell count	-0.31 (0.16)	0.058	9.4%

[a] outliers identified by robust regression of outcome measure on DNA methylation group (high/low) then removed and remaining values natural log transformed; [b] genotype coded as minor (A) allele carriage (0,1,2), assuming an additive genetic model.

Supplemental Table 12. GTEx portal results for *F2RL3* (last accessed: 04/07/2018)[46]

Gencode Id	Gene	Variant Id	SNP Id	P-Value	NES[a]	Tissue
ENSG00000127533.3	*F2RL3*	19_16856864_C_T_b37	rs773916	0.0000014	0.82	Minor Salivary Gland
ENSG00000127533.3	*F2RL3*	19_16950763_C_T_b37	rs3848656	0.000033	-0.3	Esophagus - Mucosa
ENSG00000127533.3	*F2RL3*	19_16966594_C_T_b37	rs62128035	0.000026	-0.3	Esophagus - Mucosa
ENSG00000127533.3	*F2RL3*	19_16969365_C_T_b37	rs62128036	0.0000029	-0.32	Esophagus - Mucosa
ENSG00000127533.3	*F2RL3*	19_16969855_T_C_b37	rs2313234	0.0000067	-0.31	Esophagus - Mucosa
ENSG00000127533.3	*F2RL3*	19_16976289_T_C_b37	rs2303091	0.000012	-0.3	Esophagus - Mucosa
ENSG00000127533.3	*F2RL3*	19_16997607_C_A_b37	rs4346313	0.0000012	-0.33	Esophagus - Mucosa
**ENSG00000127533.3**	***F2RL3***	**19_17000632_G_A_b37**	**rs773902**	**0.000011**	**-0.25**	**Esophagus - Mucosa**
**ENSG00000127533.3**	***F2RL3***	**19_17000632_G_A_b37**	**rs773902**	**0.000018**	**-0.19**	**Adipose - Subcutaneous**
ENSG00000127533.3	*F2RL3*	19_17003553_G_A_b37	rs2227357	0.0000079	-0.24	Adipose - Subcutaneous
ENSG00000127533.3	*F2RL3*	19_17005029_C_T_b37	rs2227359	0.0000028	2	Small Intestine - Terminal Ileum
ENSG00000127533.3	*F2RL3*	19_17006066_C_CA_b37	rs2227364	0.0000028	2	Small Intestine - Terminal Ileum
ENSG00000127533.3	*F2RL3*	19_17006110_G_A_b37	rs2227365	0.0000072	0.56	Brain - Nucleus accumbens (basal ganglia)
ENSG00000127533.3	*F2RL3*	19_17006700_G_T_b37	rs2981473	0.0000047	0.58	Brain - Nucleus accumbens (basal ganglia)
ENSG00000127533.3	*F2RL3*	19_17006787_G_A_b37	rs2227367	0.0000072	0.56	Brain - Nucleus accumbens (basal ganglia)
ENSG00000127533.3	*F2RL3*	19_17009157_A_AT_b37	rs143120093	0.0000028	2	Small Intestine - Terminal Ileum
ENSG00000127533.3	*F2RL3*	19_17009372_A_G_b37	rs2227371	0.000025	-0.19	Adipose - Subcutaneous
ENSG00000127533.3	*F2RL3*	19_17011131_C_G_b37	rs7254010	0.0000072	0.56	Brain - Nucleus accumbens (basal ganglia)
ENSG00000127533.3	*F2RL3*	19_17011247_C_A_b37	rs7257262	0.0000024	0.58	Brain - Nucleus accumbens (basal ganglia)
ENSG00000127533.3	*F2RL3*	19_17224526_C_T_b37	rs78352768	0.0000061	-0.9	Cells - EBV-transformed lymphocytes

[a]NES = normalized effect size. The Genotype-Tissue Expression (GTEx) Project was supported by the Common Fund of the Office of the Director of the National Institutes of Health, and by NCI, NHGRI, NHLBI, NIDA, NIMH, and NINDS. The data presented in this manuscript were obtained from the GTEx Portal on 04/07/18.

Supplemental Table 13. eQTLGen results for rs773902/*F2RL3* (last accessed: 22/04/2021)[47]

P-value	ID	Chr	Pos (hg19)	ID	Symbol	Chr	Pos (hg19)	Z-score	Allele Assessed	Other Allele	Nr Cohorts	Nr Samples	FDR
7.89E-08	rs773902	19	17000632	ENSG00000127533	F2RL3	19	17001544	-5.3694	A	G	32	24247	0.00028384

FDR = false discovery rate
