# Supplementary material for: Epigenetic Regulation of F2RL3 Associates With Myocardial Infarction and Platelet Function
Source: Circ Res. 2022 Jan 6;130(3):384–400. doi: 10.1161/CIRCRESAHA.121.318836 (PMC8812435; doi:10.1161/CIRCRESAHA.121.318836)
Supplement: Supplementary file 3 [file res-130-384-s003.pdf]

Major Resources Table

In order to allow validation and replication of experiments, all essential research materials listed in the Methods should be included in the Major Resources Table below. Authors are encouraged to use public repositories for protocols, data, code, and other materials and provide persistent identifiers and/or links to repositories when available. Authors may add or delete rows as needed.

Animals (in vivo studies)

Species	Vendor or Source	Background Strain	Sex	Persistent ID / URL

Genetically Modified Animals

	Species	Vendor or Source	Background Strain	Other Information	Persistent ID / URL
**Parent - Male**					
**Parent - Female**					

Antibodies

Target antigen	Vendor or Source	Catalog #	Working concentration	Lot # (preferred but not required)	Persistent ID / URL
Active form CD41/61	BD Biosciences	340507	1/10		https://www.bdbiosciences.com/eu/reagents/research/clinical-research---ruo-gmp/single-color-antibodies/fitc-mouse-anti-human-pac-1-pac-1/p/340507
P-selectin	BD Biosciences	555524	1/20		https://www.bdbiosciences.com/eu/applications/research/t-cell-immunology/regulatory-t-cells/surface-markers/human/pe-mouse-anti-human-cd62p-ak-4-also-known-as-ak4-or-ak-4/p/555524
CD41a	Thermo Fisher Scientific	12-0419-42	1/10		https://www.thermofisher.com/antibody/product/CD41a-Antibody-clone-HIP8-Monoclonal/12-0419-42
CD61	BD Biosciences	555754	1/10		https://www.bdbiosciences.com/eu/reagents/research/antibodies-buffers/immunology-reagents/anti-human-antibodies/cell-surface-antigens/pe-mouse-anti-human-cd61-vi-pl2-also-known-as-vipl2/p/555754
IgG1, kappa isotype control	BD Biosciences	555749	1/10		https://www.bdbiosciences.com/eu/reagents/research/antibodies-buffers/immunology-reagents/anti-human-antibodies/cell-surface-antigens/pe-mouse-igg1-isotype-control-mopc-21/p/555749
Rabbit anti-CEBP Beta	Abcam	Ab32358	2.5µg (total) (This is a total amount rather than a concentration that would normally be given as it was for ChIP)		https://www.abcam.com/cebp-beta-antibody-e299-c-terminal-ab32358.html

DOI [to be added]

DNA/cDNA Clones

Clone Name	Sequence	Source / Repository	Persistent ID / URL

Cultured Cells

Name	Vendor or Source	Sex (F, M, or unknown)	Persistent ID / URL
Human coronary artery endothelial cells (HCAEC)	Promocell	unknown	Catalogue number C-12221 https://www.promocell.com/product/human-coronary-artery-endothelial-cells-hcaec/
Acute megakaryocytic leukaemia cell line, CMK	Gift from Professor Ingeborg Hers, University of Bristol	M	

Data & Code Availability

Description	Source / Repository	Persistent ID / URL
Data from the Avon Longitudinal Study of Parents and Children (ALSPAC)	ALSPAC	http://www.bristol.ac.uk/alspac/researchers/data-access/
In vitro experimental data	data.bris (https://data.bris.ac.uk/data/)	See main manuscript for URL.

Other

Description	Source / Repository	Persistent ID / URL
Cell culture media for HCAEC: Endothelial Cell Growth Medium MV 2	Promocell MV2 C-22121	https://promocell.com/product/endothelial-cell-growth-medium-mv-2/
Cell culture media for CMKs: IMDM + GlutaMAX, supplemented with 10% FBS and penicillin/streptomycin	Thermofisher 31980030, Thermofisher 10500064, Thermofisher 15140122	https://www.thermofisher.com/order/catalog/product/31980030#/31980030 https://www.thermofisher.com/order/catalog/product/10500064?SID=srch-hj-10500064#/10500064?SID=srch-hj-10500064 https://www.thermofisher.com/order/catalog/product/15140122#/15140122
Cell culture media for HEK-293: Minimum Essential Medium Eagle, supplemented with 10% FBS and with 0.292 g/L L-glutamine.	e.g., Sigma M5650	https://www.sigmaaldrich.com/GB/en/product/sigma/m5650

DOI [to be added]
